# Intermolecular Carbon–Carbon Naphthalene Coupling Involves a Radical Mechanism Driven by Cytochrome P450 in *Alternaria alternata*

**DOI:** 10.1002/anie.2505284

**Published:** 2026-09-18

**Authors:** Jia Gao, Leyao Chen, Karin Steffen, Henrik Schweder, Birgit Schreckenberger, Shu‐Ming Li, Ben Auxier, Antonis Rokas, Reinhard Fischer

**Affiliations:** ^1^ Dept. of Microbiology, Institute for Applied Biosciences Karlsruhe Institute of Technology (KIT) ‐ South Campus Karlsruhe Germany; ^2^ Department of Pharmacy, Institute of Pharmaceutical Biology and Biotechnology Marburg University Marburg Germany; ^3^ Department of Biological Sciences Vanderbilt University Nashville Tennessee USA; ^4^ Evolutionary Studies Initiative Vanderbilt University Nashville Tennessee USA; ^5^ Laboratory of Genetics Wageningen the Netherlands

**Keywords:** alternaria, altertoxin, cercosporin, cytochrome P450, melanin, mycotoxin, palmarumycin, perylenequinone

## Abstract

Dihydroxynaphthalene (DHN) derivatives are precursors of biologically important molecules, including melanin and perylenequinones (PQs). Biosynthesis of DHN‐derived metabolites requires precise spatial control of radical reactions to form specific C─C or C─O─C bonds. Investigating fungal PQ biosynthesis in *Alternaria alternata*, we identified a cytochrome P450 enzyme that catalyzes a highly regio‐ and stereospecific C─C radical coupling reaction. Conversely, a DHN‐converting P450 from *Berkleasmium* sp. utilizes a diradical mechanism yielding a C─O─C bridge. Comparing these enzymes revealed five sequence motifs that, with structural modeling, allowed the identification of a novel *A. alternata* enzyme converting 1,8‐DHN to a 2‐2' DHN dimer. We demonstrate that P450‐dependent PQ biosynthesis in *A. alternata* is fundamentally distinct from the laccase‐fasciclin‐mediated pathway in *Cercospora beticola*, indicating that PQ biosynthesis evolved via distinct, lineage‐specific pathways through convergent evolution. Finally, PQs contribute to microbial niche shaping on tomato fruits. Understanding these pathways provides a foundation for producing PQs for medical applications.

## Graphical abstract

1,8‐DHN is an important intermediate to form complex aromatic ring systems. Phenol‐coupling reactions are important for the dimerization of two molecules. Here, we show that a fungal cytochrome P450 enzyme catalyzes specific C─C bonds.

## 1 Introduction

Secondary metabolites are important natural products from bacteria, plants, and fungi with a huge impact on human society [1, 2]. One large class is toxic compounds that contaminate food and feed and hence cause billion‐dollar losses in agriculture annually, whereas other classes are pharmacologically active and power a billion‐dollar market of pharmaceuticals [3]. A large proportion of drugs on the current market are natural products or are derived from natural products as lead structures [4, 5]. In both contexts, whether toxins or therapeutics, understanding the biosynthesis and genetic regulation is essential for developing strategies to prevent toxin production, modify drug molecules, or improve pharmaceutical production [6].

Several secondary metabolites are derived from two equivalents of dihydroxynaphthalene (DHN). Because carbon–carbon (C─C) bond formation between two aromatic rings is difficult, it often involves enzymatic radical mechanisms. For instance, laccases, which polymerize DHN to melanin introduce a free radical into the DHN molecule, where the free radical is regenerated during the coupling reaction, and hence several DHN molecules are crosslinked. This enables the formation of many different C─C bonds or carbon–oxygen–carbon (C─O─C) bridges and contributes to the stability of melanin against enzymatic degradation. However, radical mechanisms are also required if defined C─C or C─O─C bonds need to be formed. Enzymes catalyzing such radical‐induced specific coupling reactions are laccases, cytochrome P450 enzymes, or heme peroxidases [7, 8]. Why some enzymes generate C─C bonds and others C─O─C bridges has not been understood yet, although it appears logical that the substrate‐binding pocket should be crucial. Since many secondary metabolites are likely the products of phenol‐coupling reactions, one challenge is understanding the catalytic mechanisms of specific coupling reactions (Figure 1) [8].

**FIGURE 1:**
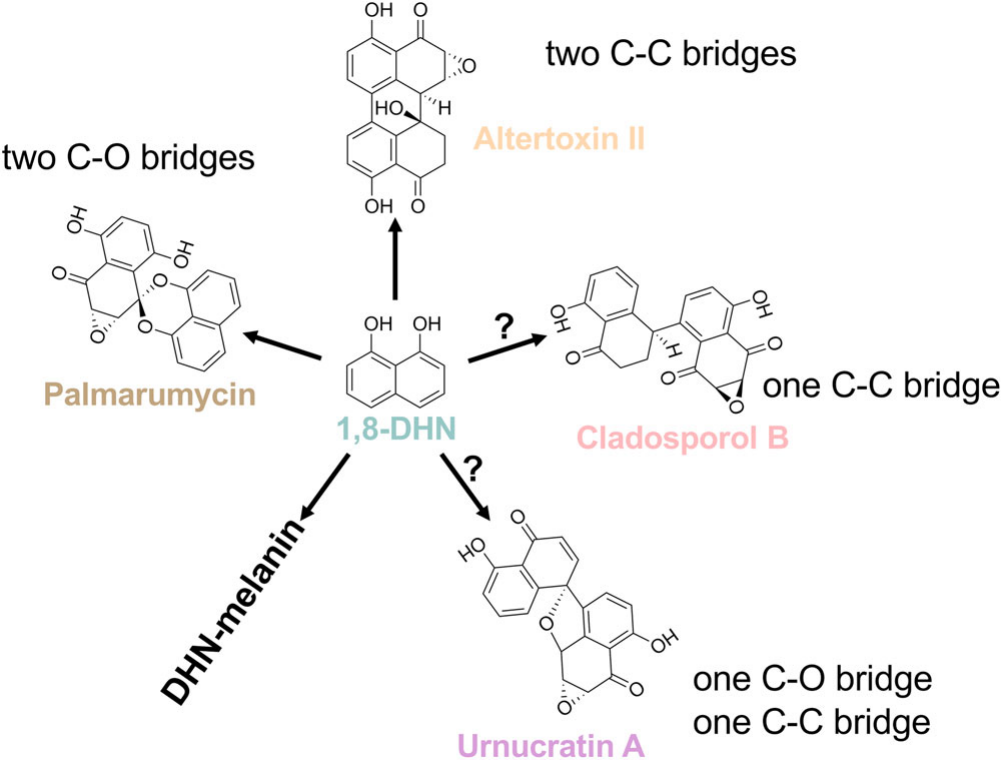
1,8‐DHN as building block of complex natural products. Two 1,8‐DHN‐derived moieties constitute a core structure linked with C─C and/or C─O─C bridges. Altertoxin II, a perylene skeleton with two C─C bridges; palmarumycin, a spirononadiene skeleton with two C─O─C bridges; urnucratin A, a bisnaphthospiroether skeleton with one C─C bond and one C─O─C bridge; cladosporol B, a cladosporol skeleton with one C─C bond; DHN‐melanin, an unsolved structure with numerous C─C bonds and C─O─C bridges.

Examples for DHN‐derived secondary metabolites are the *Alternaria alternata* altertoxins (ATXs) which belong to the class of perylenequinones (PQs) (Figure 1). PQs are very important, highly active compounds produced by fungi, plants, and a few animals [9, 10]. In plant‐pathogenic fungi like *Cercospora beticola* the PQ cercosporin is a virulence factor, and the PQ altertoxin II of *A. alternata* has cytotoxic, genotoxic, and mutagenic potential in vitro [11]. On the other hand, altertoxin II has potential as an anti‐cancer drug [12]. The toxicity is due to light‐induced generation of reactive oxygen species, which opens new avenues for the development of photodynamic therapies [13, 14]. The mutagenic effect is probably due to direct DNA interaction [15].

PQ biosynthesis in *A. alternata* initially shares the pathway with the melanin biosynthesis pathway up to the stage of the common intermediate 1,8‐DHN (1,8‐dihydroxynaphthalene) [16]. Cell‐biological analyses of melanin and PQ formation revealed spatial separation; melanin biosynthesis occurs in the aerial mycelium, mainly in spores, whereas PQs are formed in substrate hyphae. In *C. beticola*, the biosynthesis of cercosporin was studied to some extent and revealed a biosynthetic gene cluster (BGC) encoding enzymes that convert acetyl‐CoA and malonyl‐CoA to cercoquinone C, which then dimerizes to form cercosporin derivatives [17, 18, 19]. DHN is not an intermediate in this case. Homologs of enzymes encoded in the cercosporin gene cluster were not found in *A. alternata*. The long evolutionary distance between *C. beticola* (class Dothideomycetes; order Mycosphaerellales) and *A. alternata* (class Dothideomycetes; order Pleosporales) raises the question if PQ biosynthesis in fungi arose once during evolution or has convergently evolved independently [20].

Here, we used genome‐wide expression analyses in combination with gene deletions, overexpression approaches, and heterologous reconstitution of the pathway, combined with chemical analysis of the intermediates, to elucidate the entire biosynthesis route. Structural predictions in combination with site‐directed mutagenesis allowed us to define the active site of the central cytochrome P450 enzyme. Furthermore, we show that the *A. alternata* PQ pathway is not related to that of cercosporin in *C. beticola*. Evolutionary genomic analyses support the idea that Mycosphaerellales and Pleosporales use two distinct polyketide‐synthase‐based pathways for PQ biosynthesis. Finally, we show that PQs not only play a role as virulence factors for plant colonization but, in the case of *A. alternata*, are important for inter‐fungal competition, suggesting potential as photodynamic antifungal drugs. Photodynamic therapies with such molecules are already widely used by dermatologists to treat, for instance, skin cancer [21, 22]. Considering that the chemical synthesis has not been successful yet [23], the elucidation of additional biochemical pathways for PQ biosynthesis may foster their application.

## 2 Results

### 2.1 Perylenequinone (PQ) Biosynthesis Requires Two Gene Clusters

PQ biosynthesis shares many enzymes with the melanin biosynthetic pathway, but the enzymes converting 1,8‐DHN to PQs in substrate hyphae are still unknown (Figure 2A). To identify these PQ‐specific enzymes encoding genes, a genome‐wide expression analysis approach was used. We hypothesized that under conditions where high amounts of PQs are formed, the corresponding genes should be upregulated, whereas under conditions with low PQ production, the genes should be downregulated or repressed. To test this, we analyzed multiple strains with mutations in secondary metabolism regulators and assessed PQ production under varying growth conditions [24, 25, 26].

**FIGURE 2:**
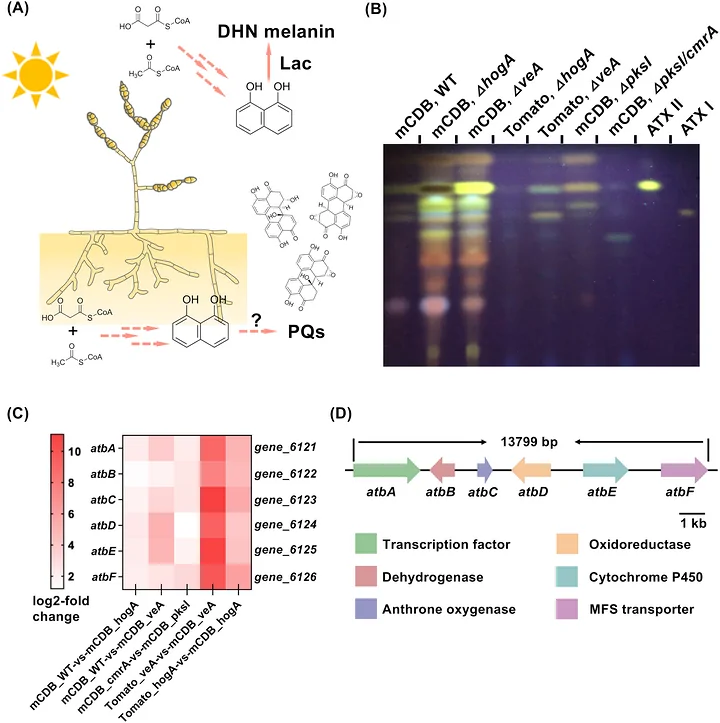
Identification of a new gene cluster involved in PQ formation by genome‐wide expression analyses. (A) In *A. alternata*, DHN‐melanin and PQs biosynthesis are spatially separated. 1,8‐DHN is polymerized by laccase to produce DHN‐melanin in spores and aerial hyphae. In substrate hyphae, 1,8‐DHN is converted to PQs through phenol coupling by unidentified enzymes. (B) Thin layer chromatography assay of extracts from *A. alternata* strains grown on liquid mCDB medium or mCDB plus tomato puree (400 g/liter) medium for 60 h at 28°C. (C) Heat map of log_2_‐fold changes for six genes with consecutive numbers from *gene_6121* to *gene_6126*. *gene_6121* to *gene_6126* were named as *atbA to atbF*, respectively. (D) Scheme of the *atb* gene cluster.

*A. alternata* strains lacking the high‐osmolarity glycerol MAPK protein HogA or the velvet‐complex protein VeA produced significantly more PQs in mCDB liquid medium in submerged culture than WT (Figure 2B). In contrast, the *pksI/cmrA* double mutant strain failed to produce PQs under the same conditions. The *pksI* gene is required for alternariol formation, and CmrA is the regulator of the DHN‐melanin gene cluster [27, 28]. Deletion of *pksI* reduces the amount of secondary metabolites and facilitates the analysis of other polyketide‐derived molecules such as PQs. In the *cmrA* mutant, the precursor for PQ formation, 1,8‐DHN, is missing. Supplementing mCDB medium with tomato puree (400 g/liter) highly reduced the production of PQs in WT [16] and also in the *hogA*‐ and *veA*‐deletion strains (Figure 2B). To account for potential growth‐related effects on PQ accumulation, colony diameters were measured when grown on mCDB medium or mCDB medium containing tomato puree (Figure S1A). On mCDB medium, the *hogA* and *veA* mutants grew more slowly than WT but still accumulated higher levels of PQs. Conversely, on tomato‐supplemented mCDB, the *veA* mutant grew faster than WT, while the *hogA* mutant showed no significant difference in growth. The absence of CmrA had no impact on the growth of the *pksI* mutant on mCDB. These results show that the accumulation of PQs is either negatively influenced by or independent of the growth rate.

After the qualitative analysis of PQs, PQ production was quantified using HPLC analysis with altertoxin I (purchased) and altertoxin II (purified) as standards (Figure S1B). Five peaks from the samples of WT and the *veA‐*deletion strain were identified as PQs (Figure S1C). Compared to WT, the *veA‐*deletion strain produced 81‐times more ATXI and 13‐times more ATXII (Figure S1D).

Following the evaluation of the PQ‐producing capacities of the different mutant strains and the strains grown under different growth conditions, corresponding transcriptome analyses were performed. Sixty‐three differentially expressed genes (DEGs) were identified as commonly upregulated among all compared groups where more PQs were produced (Figure S2A). Potential functions of these DEGs were assigned based on their InterPro classification (https://www.ebi.ac.uk/interpro/) (Figure S2B). Almost half of the identified genes code for enzymes involved in secondary metabolism in fungi, such as acyl‐CoA N‐acetyltransferase, alcohol dehydrogenase, alpha/beta hydrolase, short‐chain dehydrogenase, oxidases, oxidoreductases, peroxidases, P450 enzymes, transcription factors, and transporters. Furthermore, a scytalone dehydrogenase, which is required for the formation of 1,8‐DHN melanin and PQs, was also identified. 16 DEGs were identified as commonly downregulated under conditions where lower ATX amounts were produced (Figure S2C). Those were the genes presumably encoding O‐methyltransferase, lysophospholipase, NAD(P)‐binding domain protein, beta‐1,3‐glucanase, sugar/inositol transporter, transcription factor, phosphatidic acid phosphatase, and soluble quinoprotein glucose/sorbosone dehydrogenase (Figure S2D).

During the evaluation of candidate enzymes for PQ biosynthesis, we noted that six genes were found in a continuous block (*gene_6121* to *gene_6126*), suggesting a gene cluster in the genome (Figure 2C,D). All six genes were upregulated based on log2‐fold change values (>1, *p*<0.05). The six genes code for a putative transcription factor (TF), a dehydrogenase, an anthrone oxygenase, an oxidoreductase, a cytochrome P450, and a major facilitator superfamily (MFS) transporter (Figure 2D). The cluster resembles other fungal BGCs encoding a TF, biosynthetic enzymes, and a transporter. However, the cluster had not been bioinformatically predicted in any fungus previously, presumably because it lacks a backbone gene encoding e.g., a polyketide synthase or a non‐ribosomal peptide synthetase.

To test whether the gene cluster is required for PQ biosynthesis, we individually deleted each of the six genes using CRISPR/Cas9 (Table S1). Mutants were successfully generated for five genes, but not for the transporter‐encoding *gene_6126*. Deletion of four of the genes completely abolished PQ biosynthesis, while deletion of *gene_6122* resulted in increased PQ levels compared to WT (Figure S3A–D). All strains appeared black, like WT, suggesting that melanin biosynthesis was not affected (Figure S3A). These results demonstrate that the five genes are specifically involved in the biosynthesis of PQs. We named them *atbA‐E* (altertoxin biosynthesis) (*gene_6121* to *gene_6125*). Deletion of *gene_6126* was unsuccessful, despite screening of hundreds of transformants by PCR. Bioinformatic analysis predicted this gene encodes a transporter, leading to the hypothesis that its deletion is lethal due to intracellular accumulation of PQs. ATXII has been shown to generate reactive oxygen species (ROS) upon light exposure [29]. Supporting this hypothesis, *A. alternata* produces PQs indeed already during growth on the regeneration medium used for the generation of transgenic strains (Figure S3C). However, when we used the ∆*atbA* mutant (lacking PQ production) as the recipient for the deletion construct, *gene_6126* was successfully deleted. This deletion had no effect on 1,8‐DHN melanin production (Figure S3B). Hence, *gene_6126* encodes a transporter and is a part of the PQ biosynthetic gene cluster. We named this gene *atbF* (Figure 2D).

To confirm the involvement of AtbA‐E in PQ biosynthesis, all five corresponding deletion strains were re‐complemented with wild‐type copies of the respective genes. Re‐introduction of *atbA, atbC, atbD*, and *atbE* restored PQ production, while re‐complementation of the ∆*atbB* strain reduced PQ levels to those of the wild type. Specifically, the ∆*atbB* mutant had produced 46‐fold more ATX I and 16‐fold more ATX II than WT, whereas the re‐complemented strain returned to WT levels (Figure S3D). These results indicate that AtbC‐E are essential for the dimerization and further conversion of 1,8‐DHN or related naphthalene derivatives, while AtbA and AtbB serve regulatory functions. Whereas AtbA probably acts at the transcriptional level, the regulatory function of AtbB appears to be more complicated because it has some similarity to dehydrogenases. One possibility is that AtbB is involved in the biosynthesis of another secondary metabolite with shared intermediates with the PQ biosynthesis. Absence of AtbB would then allow the production of more PQs. The increased PQ production observed in the *atbA* re‐complemented strain may result from ectopic integration of *atbA* outside the native gene cluster. This strain represents a useful tool for studying the effects of PQ overexpression (see below).

### 2.2 The Cytochrome P450 Enzyme Modifies the Substrate and Couples Two Naphthalene Molecules

To characterize the biosynthetic pathway, we analyzed the accumulation of intermediates in ∆*atbC*, ∆*atbD*, and ∆*atbE‐*deletion strains. Because intermediates were initially undetectable (Figure S4A), we employed an AtbA‐overexpressing strain to boost production (Figure 3A). HPLC and LC‐MS/NMR analyses (Figure 3A,E) revealed that both *∆atbC* and *∆atbE* strains (with *atbA* overexpressed) accumulated 1,8‐DHN (**7**) and scytalone (**6**), indicating that AtbC and AtbE work together. The ∆*atbD* strain accumulated altertoxin IV (**8**), alterperyleneepoxide A (**9**), and altertoxin XIII (**10**). Based on these data, we propose that 1,8‐DHN (**7**) is coupled by AtbC and AtbE to yield **8‐10**, which are then oxidized to **1‐5** by the oxidoreductase AtbD. To confirm that indeed 1,8‐DHN is the main substrate of AtbC/E we performed a feeding experiment with a *pksA*‐deletion strain which cannot produce 1,8‐DHN [16] and overexpressed AtbC and E. After addition of 1,8‐DHN to the medium compounds **1, 2, 3** and **5** were detected. Compound **4** was not detected because it is further converted. If AtbC and E were not overexpressed, the amount of the compounds was lower (Figure 3B).

**FIGURE 3:**
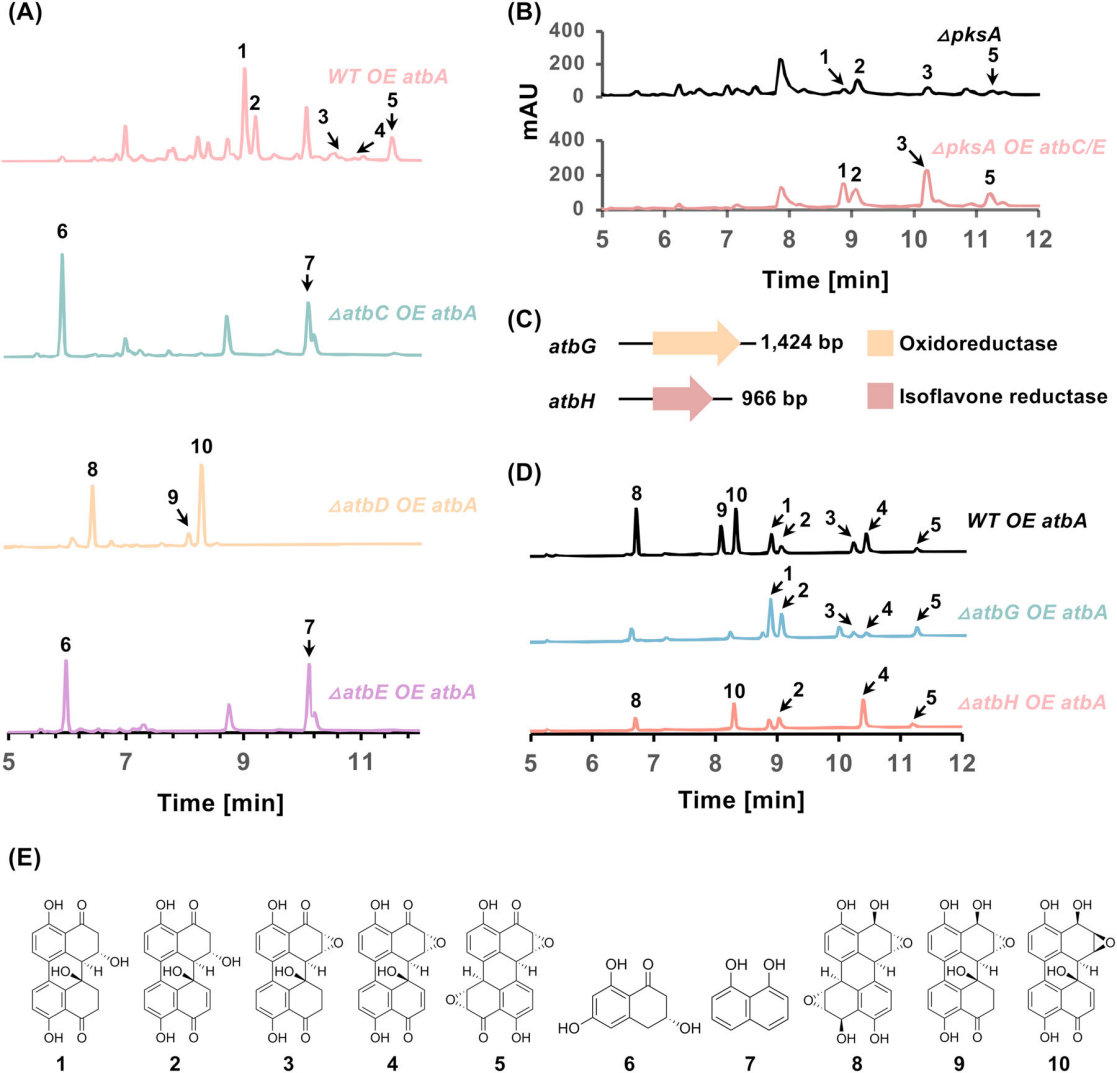
Analysis of intermediates for PQ biosynthesis. (A) HPLC chromatograms at 320 nm of *A. alternata* strains. *atbA* was overexpressed in WT, *ΔatbC*, *ΔatbD* and *ΔatbE* strains to accumulate PQs and intermediates. Extracts from *ΔatbC OE atbA, ΔatbD OE atbA and ΔatbE OE atbA* strains were analyzed by LC‐MS and NMR. Altertoxin I (**1**) alterperylenol (**2**), altertoxin II (**3**), stemphytotoxin (**4**) and altertoxin III (**5**) were detected in the extract from *WT OE atbA* strain. 1,8‐DHN (**7**) and scytalone (**6**) were detected in the extracts from *ΔatbC OE atbA and ΔatbE OE atbA* strains. Altertoxin IV (**8**), alterperylenepoxide A (**9**) and altertoxin XIII (**10**) in the extracts from *ΔatbD OE atbA* strain. (B) HPLC chromatograms at 254 nm of *A. alternata* strains fed with 0.03 mg/mL 1,8‐DHN. Compounds **1**‐**3** and **5** were detected in the extracts from *ΔpksA and ΔpksA OE atbC/E* strains. Compared to *ΔpksA* strain*, ΔpksA OE atbC/E* strain produced large amounts of compounds **1**, **3**, and **5**. (C) Scheme of *atbH* and *atbG. atbG* and *atbH* encode an oxidoreductase and an isoflavone reductase, respectively. (D) HPLC chromatograms at 320 nm of *A. alternata* strains. *atbA* was overexpressed in WT, *ΔatbG*, and *ΔatbH* strains. Compounds **1**‐**5** and **8**‐**10** were detected in the extract from the *WT OE atbA* strain. Compounds **1**‐**5** were detected in the extracts from *ΔatbG OE atbA* strain. Compounds **1**‐**5**, **8** and **10** were detected in the extract from the *ΔatbH OE atbA* strain. (E) Chemical structures of compounds **1**‐**10**.

Since the identified enzymes and intermediates (1,8‐DHN and PQ derivatives) did not fully account for all PQs produced by wild‐type *A. alternata*, we hypothesized that additional biosynthetic genes must exist and reasoned that these genes would likely be co‐regulated by the transcription factor AtbA. By comparing the transcriptomes of *∆atbA/pksI* and *∆pksI* strains, we identified 117 up‐regulated genes in the *∆pksI* background. Cross‐referencing the 117 genes with the 63 candidate genes from our previous transcriptomic analysis yielded 26 overlapping candidates (Figure S4B). From this pool, we identified *atbG* (encoding an oxidoreductase) and *atbH* (encoding an isoflavone reductase) as likely biosynthetic participants (Figure 3C). CRISPR/Cas9‐mediated deletion of these genes did not affect melanin biosynthesis, as both strains retained the black wild‐type phenotype (Figure S3E). However, PQ production was significantly altered: Compounds **1‐5** were absent in *∆atbG*, while *∆atbH* produced only **2**, **4**, and **5** (Figure S4C). To check for the accumulated intermediates, we overexpressed *atbA* in the mutant backgrounds. While the wild‐type control produced compounds **1‐5** and **8‐10**, the *∆atbG* strain still yielded **1‐5**, and the *∆atbH* strain produced **2**, **4**, **5**, **8**, and **10** (Figure 3D,E). Notably, no new biosynthetic intermediates were detected in either mutant.

To validate the identification of all genes required for PQ biosynthesis, we reconstituted the pathway in *A. brassicicola*, a species that produces only DHN melanin but no PQs. We first sequenced and assembled the genome of *A. brassicicola* DSM62008, which spans 34.08 Mb across 32 scaffolds. As expected, neither the *atb* gene cluster nor *atbH* was present in this strain (Figure S4D). However, an ortholog of *A. alternata* AtbG was identified, sharing 93.7% protein identity. Introduction of the complete *A. alternata atb* cluster (*atbA‐F*) into *A. brassicicola* resulted in the production of compounds **2**, **4**, and **5**. Similar results were obtained when *atbG* was co‐introduced with the cluster. When the cluster was introduced together with *atbH*, compounds **1‐3** and **5** were detected. Full reconstitution, yielding all five compounds **1‐5**, was achieved only when the *atb* cluster, *atbG*, and *atbH* were co‐expressed in *A. brassicicola* (Figure S4E). These results demonstrate that the *atb* gene cluster, along with *atbG* and *atbH*, is necessary and sufficient to convert 1,8‐DHN (**7**) into PQs.

Since both PQs and their precursor 1,8‐DHN are toxic to the producing cells, we next investigated whether and how the PQ biosynthetic pathway is compartmentalized. To address this, we analyzed the subcellular localization of several pathway enzymes using fluorescent translational fusion proteins. mCherry was fused to the N‐ or C‐terminus of the target proteins, with the fusion site selected based on predicted sequence motifs (Figure S5A). mCherry(N)‐AtbC, AtbD‐mCherry(C), AtbE‐mCherry(C), AtbG‐mCherry(C) and AtbH‐mCherry(C) rescued the production of PQs in *ΔatbC*, *ΔatbD*, *ΔatbE*, *ΔatbG* and *ΔatbH* strains, respectively (Figure S3F,G). The AtbB‐mCherry protein also re‐complemented the ∆*atbB* phenotype. Hence, all six fusion proteins were biologically active. AtbC, E, and F are localized to the cytoplasmic membrane, which is supported by the presence of predicted transmembrane domains. AtbD, whose protein sequence starts with a predicted signal peptide for secretion, labeled the membrane or cell wall, despite the lack of transmembrane domains. AtbB, AtbG, and AtbH are localized to the cytoplasm (Figure S5B). AlphaFold modeling revealed AtbC and AtbE could form a complex with interaction at transmembrane domains (Figure S5C).

Based on the above results, we propose the PQ biosynthetic pathway shown in Figure 4: 1,8‐DHN (**7**) is hydroxylated by AtbC and AtbE to form compound **11**. In the cytoplasm, **7** is dimerized by AtbE to produce **5**; **7** and **11** are oxidatively coupled by AtbE to produce **4**; compound **8** is formed through the reduction of two keto groups at C8 and C18 of **5** catalyzed by oxidoreductase AtbG; compound **12** is formed through the reduction of the keto group at C18 of compound **4** catalyzed by oxidoreductase AtbG; compounds **3** and **9** are formed through the reduction of the double bonds at C11 and C12 of **4** and **12** catalyzed by reductase AtbH. In the extracellular space, **3‐5** are formed through the oxidation of the hydroxyl group at C18 of **9**, **12**, and **8** by oxidoreductase AtbD; the epoxide groups of compounds **3**‐**5** are hydrolyzed to form compounds **1**, **2**, and **13**, respectively. The mechanism for the last reaction remains to be resolved. It is likely that PQs with a keto group at C18 are more toxic than PQs with a hydroxyl group at C18. That could explain why the keto group at C18 of **3**‐**5** needs to be reduced to the hydroxyl group by AtbH to form compounds **9**, **12**, and **8** in the cytoplasm. Subsequently, the transporter AtbF pumps them out of the cell, where they are oxidized by AtbD to produce again the toxic compounds **3**‐**5**.

**FIGURE 4:**
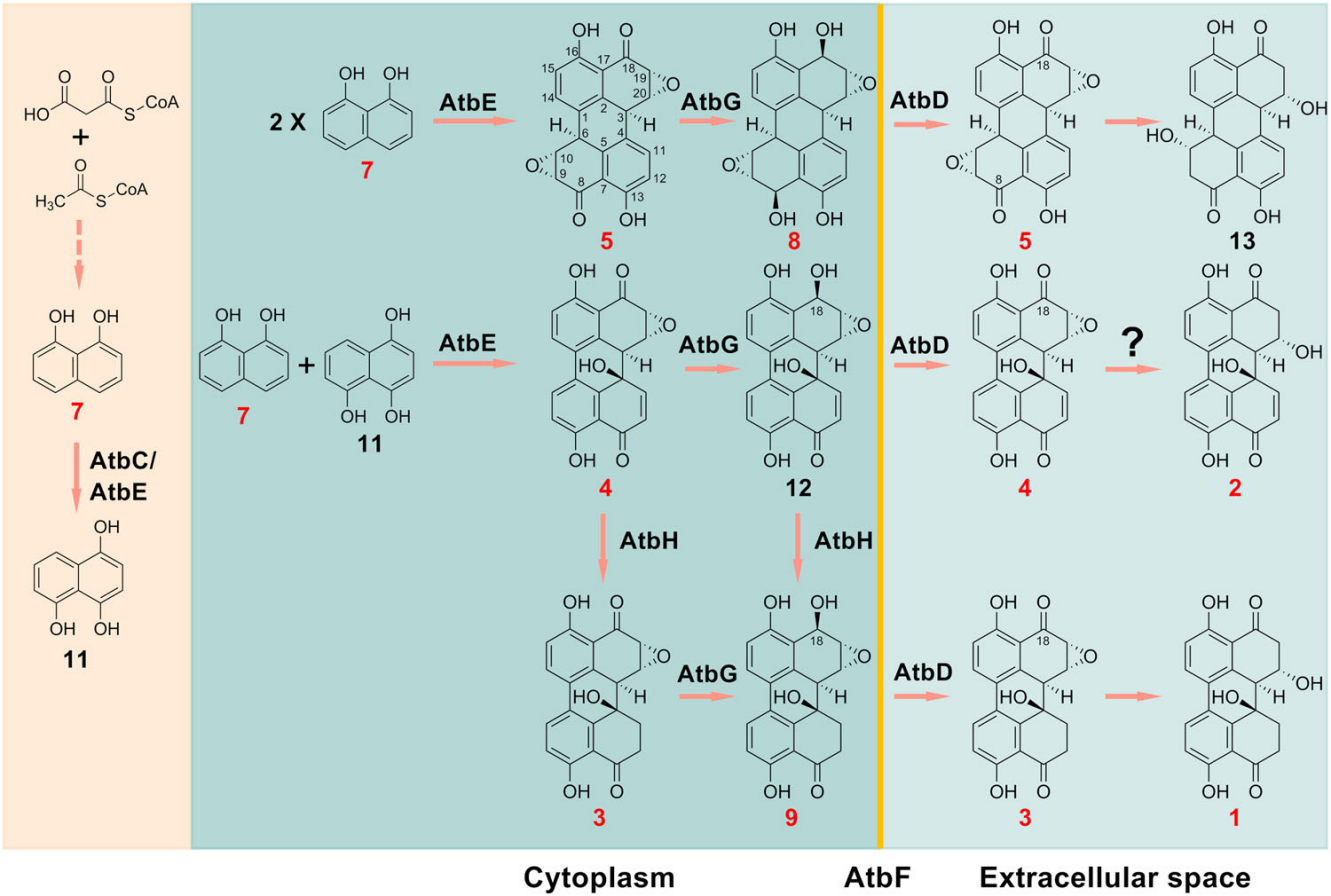
Proposed pathway for PQs biosynthesis in *A. alternata*. 1,8‐DHN is converted by AtbC and AtbE to **11**. In the cytoplasm, two molecules of compound **7** are dimerized by AtbE to **5**; compounds **7** and **11** are converted by AtbE to **4**; compound **8** is formed through the reduction of two keto groups at C8 and C18 of compound **5** converted by oxidoreductase AtbG; compound **12** is formed through the reduction of the keto group at C18 of **4** catalyzed by oxidoreductase AtbG; compounds **3** and **9** are formed through the reduction of the double bonds at C11 and C12 of **4** and **12** catalyzed by reductase AtbH, respectively. In the extracellular space, **3‐5** are formed through the oxidation of the hydroxyl group at C18 of **9**, **12**, and **8**, catalyzed also by oxidoreductase AtbD; the epoxide groups of **3**‐**5** are hydrolyzed to form **1**, **2,** and **13**, respectively. The mechanism remains to be resolved. All confirmed molecules are indicated with red numbers.

### 2.3 Molecular Docking and Mutagenesis of the Cytochrome P450 Enzyme AtbE

After the identification of all enzymes involved in PQ biosynthesis in *A. alternata*, we aimed at gaining deeper insights into the catalytic mechanism for the formation of compound **4** in the cytoplasm. Molecular docking of 1,8‐DHN (**7**) and the DHN‐derived compound **11** as ligands into P450 AtbE was performed using Boltz‐2 (AlphaFold3) (https://neurosnap.ai/service/Boltz‐2%20(AlphaFold3)). Thirteen amino acid residues within the range of 3.5 Å from the ligands (**7**) and (**11**) were detected in the substrate‐binding pocket of AtbE (Figure 5A). In this model, the hydroxyl groups of **11** could form hydrogen bonds (yellow dashed lines) with the heme. Interaction of **11** with the Fe═O in the heme group is crucial for the initial generation of the phenoxyl radical. Alanine scanning mutagenesis revealed that 12 residues were important for AtbE activity (Figure 5B). Amino acid 311 is an alanine itself and was therefore not tested for functionality. The enzyme activities were estimated according to the amounts of PQs produced by re‐complemented strains compared to PQ amounts produced by WT.

**FIGURE 5:**
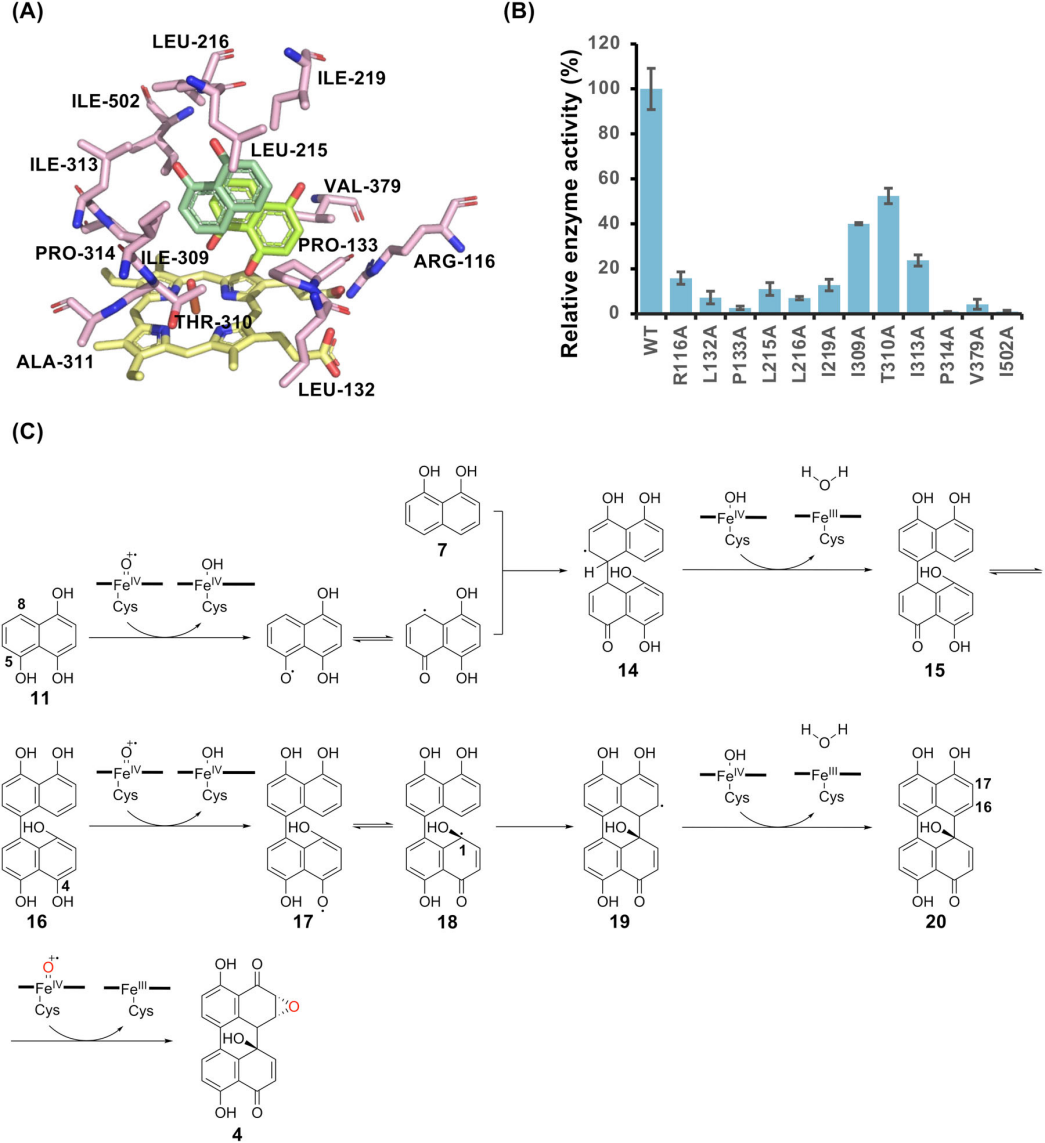
Characterization of the active site of AtbE and proposed enzyme mechanism. (A) Molecular docking using compounds **7** and **11** as ligands with AtbE. The predicted amino acid residues in the range of 3.5 Å of ligands **7** and **11** are shown in light pink; ligands **7** and **11** are shown in light green and lemon, respectively; heme is shown in light yellow. (B) Relative enzyme activities of site‐directed mutations of AtbE. *atbE* mutant alleles were re‐complemented in the *ΔatbE* strain. Enzyme activity was deduced according to the amounts of PQs produced by re‐complemented strains compared to that produced by WT. (C) Proposed mechanism for the formation of compound **4** catalyzed by AtbE.

AtbE could catalyze three rounds of oxidation starting from compounds **7** and **11** (Figure 5C). First, AtbE could abstract one hydrogen atom from the 5‐OH of **11** and generate one phenoxyl radical. The radical is delocalized to the *para* position for forming a radical at C‐8 of compound **11** through resonance. Next, compound **14** could be formed via attacking of radical **11** to **7**, where the radical is delocalized to the phenol ring of **7**. **15** is formed from **14** through hydrogen abstraction. Also, **16** is generated from **15** via spontaneous aromatization. In the second round of oxidation, AtbE could abstract one hydrogen atom from 4‐OH of **16** and form **17** with one phenoxyl radical. The electron would resonate to the *para* position to form a radical at C1‐OH of **17** through resonance. Compound **19** could be generated through a radical attack inside **18** and radical delocalization. **20** is formed from **19** via hydrogen abstraction. In the last round of oxidation, one epoxide group is introduced between C‐16 and C‐17 of **20** to produce compound **4**. The proposed sequence of oxidation events is different from the proposed P450 mechanism in *Berkleasmium sp*. where PalA couples two 1,8‐DHN to palmarumycin C_2_ (PC_2_) in a diradical mechanism to form two C─O─C bridges [30].

### 2.4 Convergent Evolution of PQ Biosynthetic Pathways

Next, we examined the distribution of PQ biosynthesis via the AtbE pathway across fungi. The biosynthetic pathway described in this work differs clearly from that described for cercosporin [18], suggesting convergent evolution of PQ biosynthesis (Figure 6A). Using sequence similarity searches, we detected the *atb* BGC in the genomes of 27 species in the Pleosporales order (class Dothideomycetes) and the *ctb* BGC in 22 species (some from class Dothideomycetes and others from Sordariomycetes). Sequence conservation against the *C. beticula* queries is low, but the overall clustering of genes in the BGC is conserved. Genes for DHN‐melanin biosynthesis were found across all genomes containing the *atb* BGC and some containing the *ctb* BGC (Figure S6). The *atb* BGC was first described in *A. alternata*. Based on the phylogenetic distribution, the *atb* BGC likely has a single origin in the ancestor of the Pleosporales, although some members of the lineage subsequently lost the BGC. The *ctb* BGC was first described in *Cercospora beticola* (order Mycosphaerellales; class Dothideomycetes) but has since been found in other genera/species in the Sordariomycetes, such as *Colletotrichum* [17, 18]. The presence of the DHN‐melanin genes in all genomes with the *atb* BGC supports the hypothesis that 1,8‐DHN is the precursor required for PQs biosynthesis via the *atb* BGC. Interestingly, two further proteins, AtbG and H, were required to achieve the full spectrum of PQs. Whereas AtbG appears to be specific for PQ producers, AtbH homologues were also identified in other fungi. However, sequence identities were low, and we cannot decide yet whether the detected proteins are orthologues or paralogues.

**FIGURE 6:**
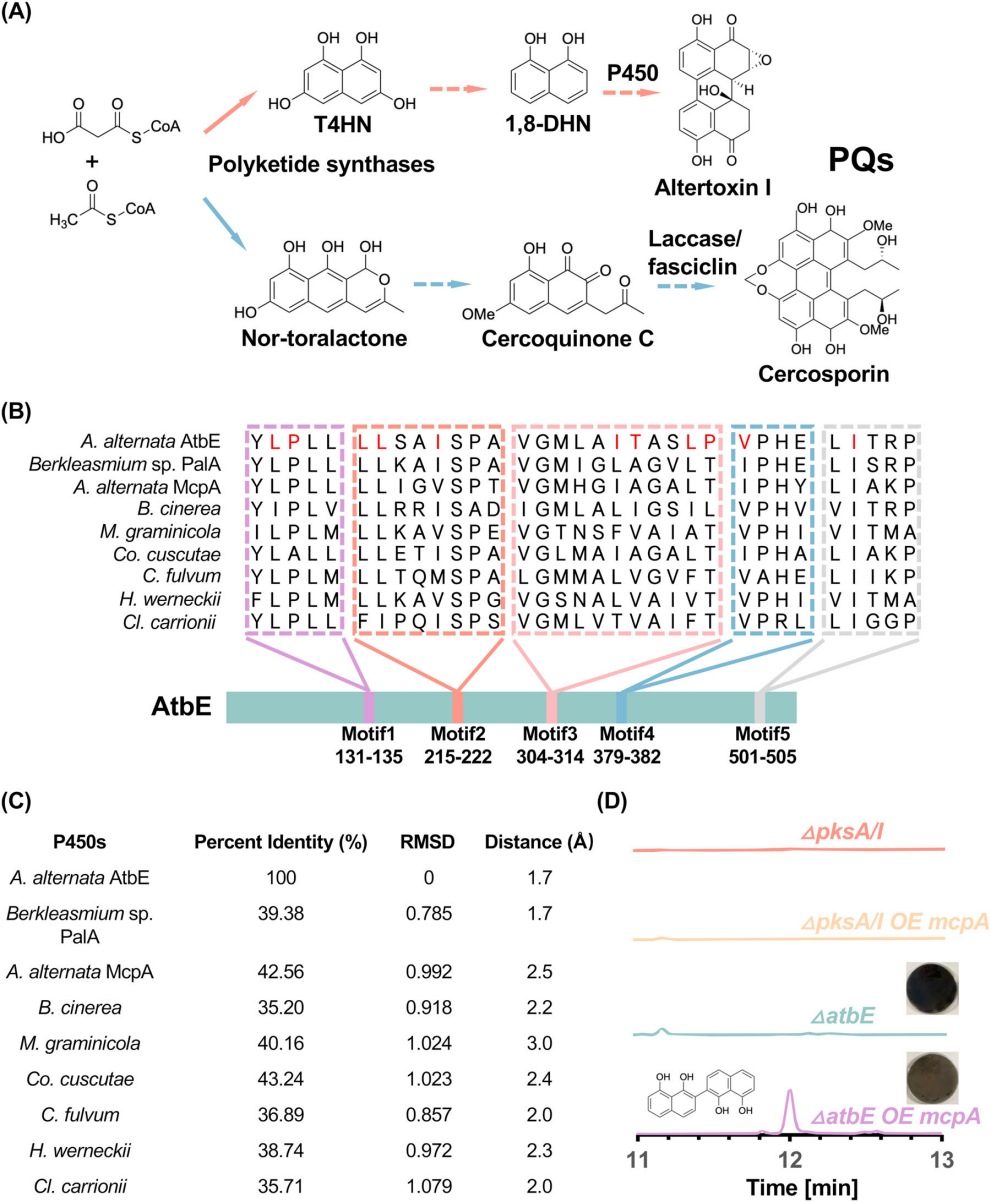
Comparison of different biosynthesis pathways involving C─C or C─O─C coupling reactions. (A) Convergent evolution of PQs biosynthesis in fungi. Malonyl‐CoA and acetyl‐CoA are converted by polyketide synthases to produce T4HN and Nor‐toralactone, respectively. T4HN is converted to 1,8‐DHN by a couple of enzymes, and then 1,8‐DHN and its derived moiety are coupled by P450 to produce PQ, for instance, altertoxin I; cercoquinone C is formed through reactions of diverse enzymes, followed by being dimerized by a laccase/fasciclin complex and other modifications to produce cercosporin. (B) Comparation of *A. alternata* AtbE and *Berklesmium sp*. PalA revealed five conserved sequence motifs. Protein sequence alignment of P450s from other fungi with AtbE. Amino acids important for activity of AtbE are written in red. (C) Potential ability of forming 1,8‐DHN‐derived secondary metabolites by P450s is evaluated through protein sequence similarity, structure similarity of AtbE and distance between hydroxyl group of 1,8‐DHN and Fe═O of heme, with 1,8‐DHN converting P450 PalA as a positive control. Around 35%–43% identity indicated middle confidence in functional conservation between AtbE and other P450s; RMSD values are less than or closed to 1 Å indicated high confidence in structure similarity between AtbE and other P450s; hydrogen bond distances between hydroxyl group of 1,8‐DHN and Fe═O of heme range from 1.7 to 3.0 Å, indicating high or middle confidence in converting 1,8‐DHN to other secondary metabolites by P450s. (D) *A. alternata* McpA dimerizes 1,8‐DHN. The overexpression of McpA in a melanin‐deficient strain *ΔpksA/I* did not change the HPLC profile at 254 nm; the overexpression of McpA in the *ΔatbE* strain led to the detection of 2‐2'‐DHN dimer. The *ΔatbE OE mcpA* strain was less melanized than the parental strain as indicated by the color of the agar plates.

To prove that other fungi harboring the *atb* BGC can synthesize PQs, we analyzed *Stemphylium lycopersici* and *Bipolaris maydis*, whose genomes contain the BGC (Figure S7A). Although many *Stemphylium* species have been shown to produce stemphyltoxin I‐III and stemphyperylenol, *S. lycopersici* did not produce PQs [31], suggesting that the genes were not active under the tested conditions. Likewise, we did not detect PQs with *B. maydis*. However, ATX I, ATX II and other PQs were produced when *B. maydis atbA* was overexpressed (Figure S7B). *B. maydis atbA* also rescued the *A. alternata* ∆*atbA* strain, suggesting conserved functions (Figure S7C).

### 2.5 Prediction of Cytochrome P450s With 1‐8 DHN Converting Ability to Produce Secondary Metabolites in Fungi

Next, we compared PalA from *Berkleasmium* sp. [30], which forms a C─O─C bridge, with *A. alternata* cytochrome P450 (AtbE), which forms C─C bonds. We aimed at defining some conserved sequence or structural features to be able to predict catalytic activities of candidate cytochrome P450 enzymes. First, we analyzed how common P450 cytochromes with the ability to convert 1,8‐DHN to toxic PQs were among fungal pathogens. Homologues of AtbE and PalA were found in the plant pathogens *Mycosphaerella graminicola*, *Colletotrichum cuscutae*, *Botrytis cinerea*, and *Cladosporium fulvum*, the human pathogenic fungus *Cladophialophora carrionii*, and the black yeast *Hortaea werneckii*. One candidate was also identified in *A. alternata* and named McpA (Melanin‐converting protein A) (Figure S8). Further related enzymes, which catalyze phenol coupling reactions, were also found in other ascomycetes [7]. This preliminary analysis suggests separation of two groups of enzymes during evolution (Figure S8). Because AtbE and PalA convert similar substrates we tested whether this would be reflected in conserved sequence stretches. Indeed, we found five conserved peptide motifs, which were also found in McpA of *A. alternata*, and also in the enzymes of several other fungi. Amino acids that affect the activity of AtbE are shown in red (Figure 6B). Besides sequence comparisons, structural similarities with AtbE and distances between the hydroxyl groups of 1,8‐DHN and the heme Fe═O were studied. Around 35%–43% identity indicated moderate confidence in functional conservation between AtbE and other P450s; root‐mean‐square‐deviation values (RMSD) are less than or close to 1 Å indicating high confidence in structure similarity between AtbE and other P450s; hydrogen bond distances between the hydroxyl groups of 1,8‐DHN and Fe═O of heme range from 1.7 to 3.0 Å, indicating high or middle confidence in converting 1,8‐DHN to other secondary metabolites (Figure 6C). Molecular docking of 1,8‐DHN into cytochrome P450s was performed using Boltz‐2 (AlphaFold3). Structural similarities between AtbE and other P450s were studied with Pymol. Hydrogen bond distances were measured using Pymol. To test whether the motif‐containing enzymes may indeed dimerize DHN, we overexpressed the *A. alternata* gene, *mcpA*, in an *A. alternata* strain lacking *pksA* and hence unable to produce DHN, and in a strain lacking AtbE and hence able to produce DHN but no altertoxins. Whereas in the first strain no new substance was observed in HPLC analyses, expression in the *∆atbE* strain resulted in a new compound with a retention time of about 12 min. LC‐MS/NMR analyses revealed that this compound was the 2‐2′ DHN dimer. The colony appeared to be less melanized than the recipient strain, suggesting the conversion of DHN (Figure 6D). This *proof‐of‐principle* experiment should foster further studies to systematically search for novel enzymes and novel PQs or other DHN‐derived secondary metabolites [8].

### 2.6 *A. alternata* Uses PQs to Compete With Other Fungi in Their Natural Habitat

Purified altertoxins (ATXs) exhibit broad‐spectrum inhibitory activity against fungi, bacteria, and plants. Furthermore, cercosporin—a related perylenequinone (PQ) produced by *Cercospora* species—is a known virulence factor during plant infection [32]. Previously, in vivo analysis of ATXs was hampered by the inability to generate ATX‐deficient mutants without simultaneously disrupting melanin biosynthesis, as both pathways share the same initial steps. Given that DHN‐melanin protects fungi against UV radiation and reactive oxygen species (ROS), identifying the PQ‐specific *atb* gene cluster was essential to decouple melanin and PQ functions.

We characterized the *∆atbA* strain (lacking PQs) and the *atbA*‐complemented strain (overproducing PQs) across mCDB, PDA, and MM media (Figure S9A,B), confirming that melanization remained unaffected. Infection assays on tomato and apple fruits using the wild‐type (WT), deletion, and complemented strains revealed no significant differences in lesion development.

Next, we hypothesized that PQs are utilized by *A. alternata* to compete with other microorganisms. To test this hypothesis, we isolated two fungal species from tomato fruits. They were characterized by ITS sequencing as *Penicillium crustosum* and *Penicillium olsonii*, respectively (Figure S10). In competition experiments, 1 × 10^4^ spores of *A. alternata* were mixed with the same number of conidia from *P. crustosum* or *P. olsonii* and spread evenly on mCDB medium (Figures 7A and S11A). Growth was evaluated after 5 days at room temperature. In addition to the isolates, we tested the model fungus *A. nidulans* in competition assays on agar plates and found that co‐cultivation induced secondary metabolite production in *A. alternata* (Figure S11B). The *Alternaria* strains outcompeted the two Penicillium strains if PQs can be produced (Figures S11B and S12). *A. nidulans* behaved similarly, but *A. alternata* was always dominant, likely also because *A. dolans* do not grow well at room temperature.

**FIGURE 7:**
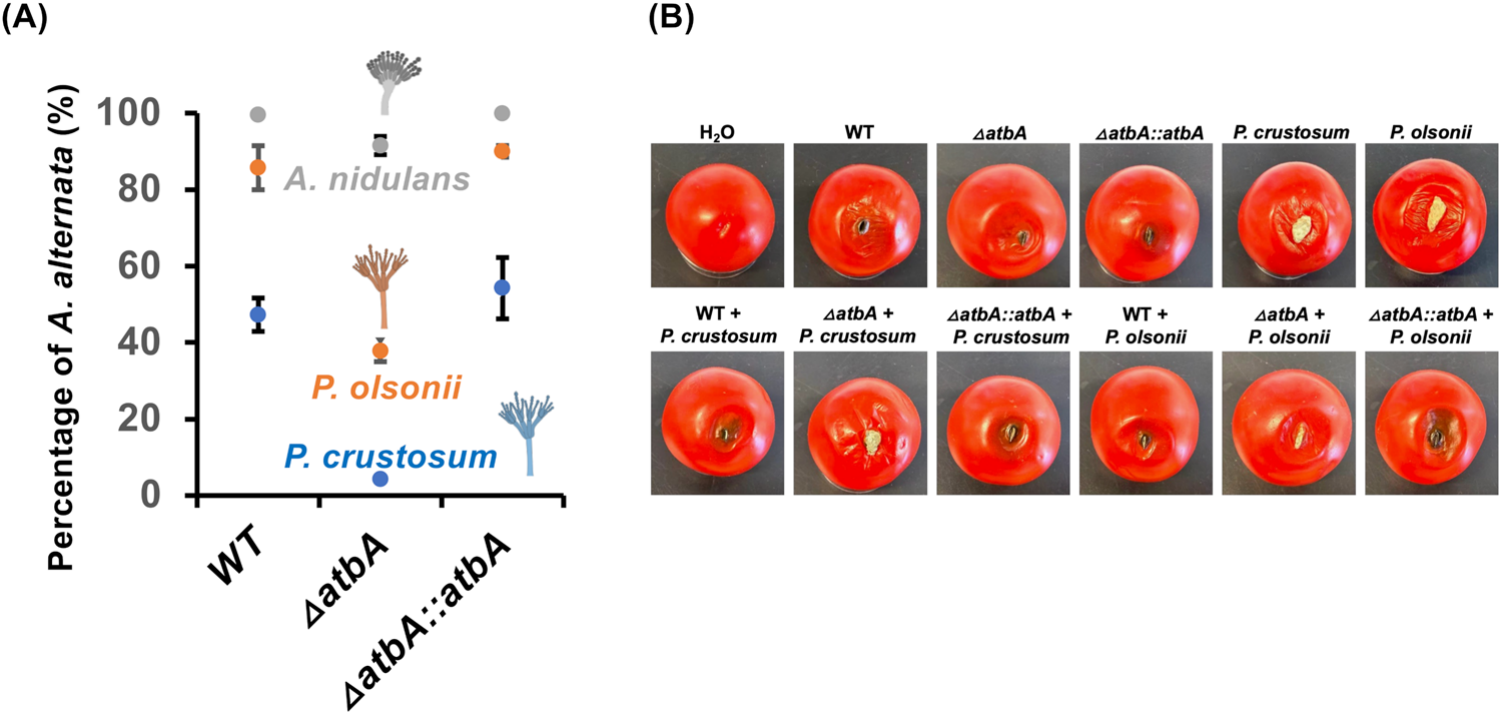
*A. alternata* uses PQs to compete with other fungi. (A) Quantification of ratios of *A. alternata* strains when competing with *P. crustosum*/*P. olsonii*/*A. nidulans* using qPCR values of *tefA* gene. (B) Growth of *A. alternata* strains (WT, *ΔatbA* and the re‐complemented strain *ΔatbA::atbA*), *P. crustosum*, *P. olsonii*, and *A. alternata* strains competing with *P. crustosum* / *P. olsonii* on tomato fruits for 7 days at room temperature.

To validate our findings in the natural habitat, we performed co‐inoculation experiments on tomatoes. *A. alternata* infection typically yields lesions with dark hyphae, while *Penicillium* species induce lesions with a whitish mycelial center. In co‐inoculation assays with *A. alternata* wild‐type (WT) and either *P. crustosum* or *P. olsonii*, *A. alternata* consistently dominated. The *∆atbA::atbA* overexpression strain, which produces more altertoxins, exhibited the same dominant phenotype. Conversely, when the *∆atbA*‐deletion strain (lacking altertoxin production) was used, the *Penicillium* strains outcompeted *A. alternata* (Figure 7B). Collectively, these results demonstrate that *A. alternata* PQs are critical for interspecies competition within natural environments.

## 3 Discussion

Bacteria, plants, and fungi are rich sources of secondary metabolites, many of which have important applications as drugs or act as mycotoxins contaminating food and feed [3]. One intriguing group of secondary metabolites are perylenequinones (PQ), which are formed by oxidative coupling of two naphthalene rings [6]. Perylenequinones (PQs) and other related metabolites are important secondary metabolites with cancer‐promoting or ‐suppressing effects, primarily produced by phytopathogenic fungi [6, 11] (Figure S13). Beyond fungi, some plants and animals also produce PQs, but they cannot be synthesized yet [8, 9, 10, 23]. The biosynthesis in fungi and plants has been studied in only a few species; a key step involves the coupling of two naphthalene derivatives to form the distinctive four‐ring structure [18, 33, 34]. By iteratively correlating PQ biosynthesis with gene expression data, we identified a second gene cluster and additional genes outside the cluster essential for PQ production in *A. alternata*. While laccases, flavin‐dependent monooxygenases, or peroxygenases such as those found in *Cortinarius odorifer* have been proposed as potential catalysts [35, 36], we demonstrate here that in *A. alternata*, this reaction is catalyzed by a cytochrome P450, AtbE. Structure predictions of the active site suggest space for two naphthalene rings, which are held in place by hydrogen bonds and probably also by pi–pi‐stacking to enable site‐specific C─C bonds (Figure 8A and Movie S1). This is fundamentally different from melanin formation by laccases, where only one DHN is the substrate for radical formation (Figure 8A and Movie S2) [7]. The DHN molecule is then released, and the very reactive radical induces different bonds between adjacent DHN molecules [37, 38]. This explains the large number of polymerized DHN units (up to 50) and the variety of different bonds in melanin. However, if defined end products need to be formed, radical formation and location need to be strictly controlled. Another example of such a controlled radical mechanism is PalA from *Berkleasmium* [30]. In that case, the predicted arrangement of the molecules in the active site leads to C─O─C bridges (Figure 8A and Movie S3). The predicted binding pockets of the two P450 enzymes also allow the channeling of the product through the protein, whereas in the case of the laccase, the space is restricted to bind DHN (Figure 8A). It is intriguing that AtbE forms a C─C and PalA a C─O─C bond during the coupling reaction. We propose that this is mainly due to the orientation of the precursors in the active center. The one‐radical mechanism proposed for AtbE depends on the orientation of compound **11** with the OH group pointing toward the Fe═O of the heme cofactor and the OH groups of 1,8‐DHN pointing away from the heme. This results in C─C bond formation (Figure 8B). In the case of PalA, the two hydroxylated substrates point with their hydroxyl groups toward the heme and a diradical mechanism induces C─O─C bridge formation (Figure 8B). Our analyses allow predictions of the reaction mechanisms and products of new P450 enzymes if the substrates are known.

**FIGURE 8:**
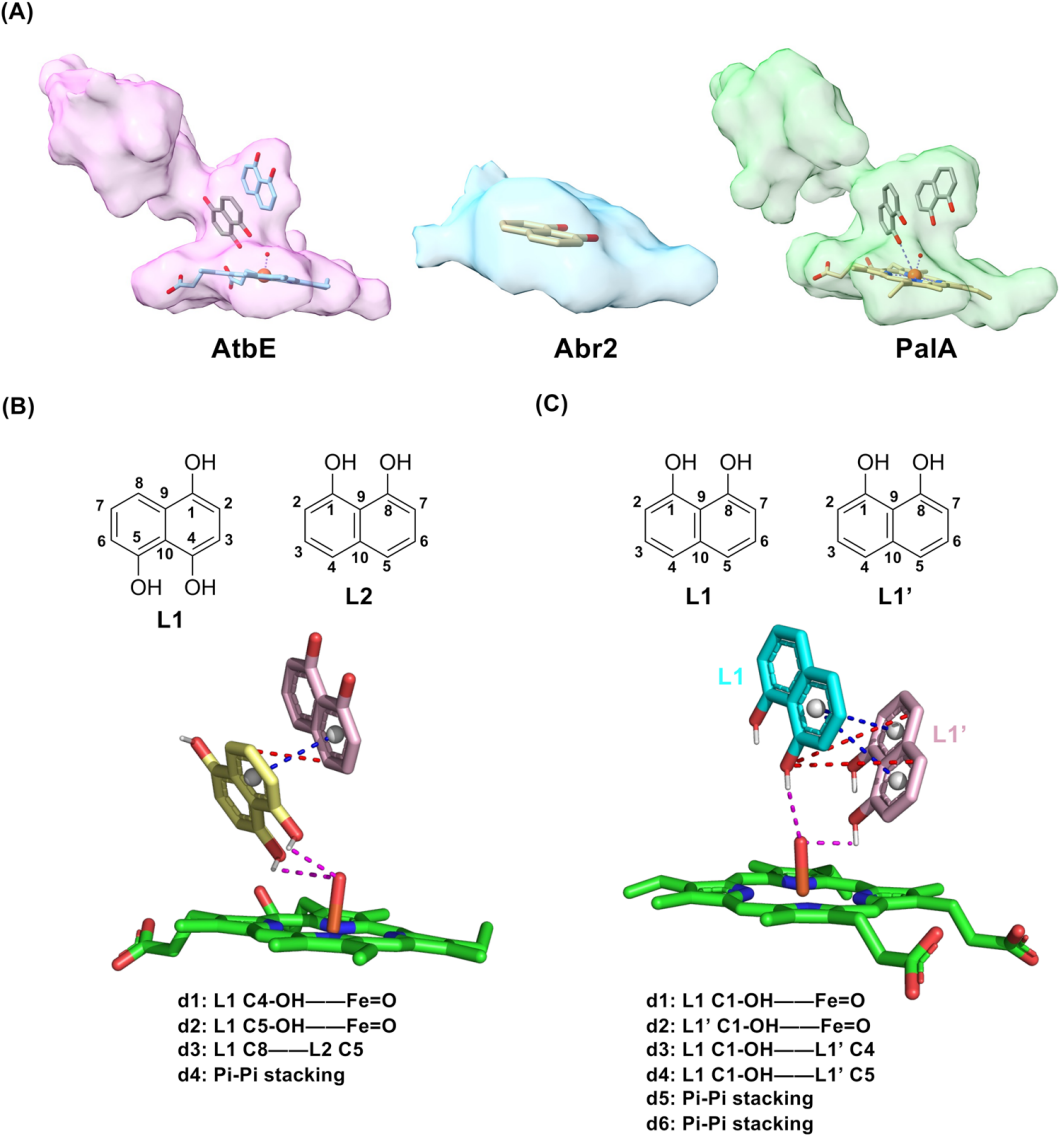
Specific C─C bonds and C─O─C bridges are formed by P450s because of the distribution of two naphthalenes inside the active site cavity, hydrogen bonds with heme, and Pi–Pi stacking between two naphthalenes. (A) Comparison of the active sites of P450 AtbE, *A. fumigatus* laccase Abr2, and P450 PalA from *Berklesmium spec*. The active sites were predicted and visualized by ChimeraX using the tool “Find Cavities”. The active sites are shown in light purple (AtbE), light blue (Abr2), and light green (PalA). Whereas the P450 enzymes accommodate two naphthalenes, the laccase cavity only allows entry of one molecule. (B) Accommodation of compound **7** (L2) and compound **11** (L1) inside the active site of AtbE. Hydrogen bonds formed between L1 and heme are shown in purple; the distance between C8 of L1 and C5 of L2 is shown in red; the distance of Pi–Pi stacking between L1 and L2 is shown in blue. d1 = 2.9 Å; d2 = 2.4 Å; d3 = 4.1 Å; d4 = 3.6 Å. (C) Accommodation of two molecules of compound **7** (L1 and L1’) inside the active site of PalA. The hydrogen bond formed between L1 and heme is shown in purple; the distances between C1‐O of L1 and C4 of L1’, and the distances between C1‐O of L1 and C5 of L1’ are shown in red; the distances of Pi–Pi stacking between L1 and L1’ are shown in blue. d1 = 2.5 Å; d2 = 2.1 Å; d3 = 5.0 Å; d4 = 5.3 Å; d5 = 4.1 Å; d6 = 4.2 Å.

To date, more than 60 PQs have been identified from diverse fungal species, mostly within the Dothideomycetes and Sordariomycetes, but also in Eurotiomycetes and Leotiomycetes [10]. Our evolutionary genomic analyses suggest that PQ biosynthetic pathways have evolved at least twice independently within ascomycete fungi, once within Pleosporales (altertoxins, *atb* gene cluster) and once within Mycosphaerellales (cercosporin, *ctb* gene cluster). Notably, the *ctb* gene cluster is also found in *Colletotrichum* plant pathogens, which belong to the class Sordariomycetes; *Colletotrichum* fungi likely acquired the *ctb* gene cluster through horizontal transfer from donors in the Mycosphaerellales [17, 18]. In recent years, reports of discoveries of convergent evolution of secondary metabolite pathways in fungi (e.g., psilocybin [39]; or anthraquinones [40]), plants [41], bacteria [42] and even across kingdoms are increasing, suggesting that independent evolution of unrelated biosynthetic pathways for the production of the same secondary metabolites may be more common than previously thought [43].

Although secondary metabolites often serve as vital lead structures for pharmaceuticals, their ecological functions frequently remain elusive [38]. Here, we provide evidence that *A. alternata* produces altertoxins to gain a competitive advantage over other fungi in its natural habitat. This finding raises the question of self‐resistance [44], which we addressed in this study. We demonstrate that the toxic primary products of the coupling reaction are likely partially inactivated via the reduction of a keto group to a hydroxyl group. Following export from the cell, these intermediates are re‐oxidized to restore higher toxicity. Thus, the self‐resistance strategy of *A. alternata* relies on a sophisticated combination of cellular compartmentalization and the utilization of low‐toxicity intermediates.

## 4 Conclusion

We decoded a key enzymatic mechanism governing fungal perylenequinone biosynthesis and identified a cytochrome P450 enzyme that drives radical‐mediated C─C coupling, contrasting with a homologous enzyme that forms C─O─C bonds. Structural modeling and mutagenesis reveal that substrate orientation within the active site dictates this catalytic divergence. These insights advance our understanding of DHN‐derived metabolite assembly and open new avenues for the biocatalytic synthesis of photodynamic therapy agents.

## 5 Experimental Section

### 5.1 Strains, Plasmids and Culture Conditions

*A. alternata*, *A. brassicicola*, *A. nidulans*, *B. maydis*, *P. crustosum* and *P. olsonii* strains are listed in Table S2; *A. alternata*, *A. brassicicola*, *B. maydis* strains were grown on mCDB medium (4% glucose, 0.1% yeast extract, 0.1% NaNO_3_, 0,025% NH_4_Cl, 0.1% KH_2_PO_4_, 0.025% KCl, 0.025% NaCl, 0.05% MgSO_4_ × 7H_2_O, 0.001% FeSO_4_ × 7H_2_O, 0.001% ZnSO_4_ × 7H_2_O and 1.5% agar) or mCDB medium containing tomato puree (400 g/liter) at 28°C. *A. nidulans* was grown on minimal medium (2% glucose, 0.6% NaNO_3_, 0.15% KH_2_PO_4_, 0.05% KCl, 0.05% MgSO_4_ × 7H_2_O, 0.0005% FeSO_4_ × 7H_2_O, 0.0022% ZnSO_4_ × 7H_2_O, 0.0011% H_3_BO_3_, 0.0005% MnCl_2_ × 4H_2_O, 0.00016% CoCl_2_ × 5H_2_O, 0.00016% CuSO_4_ × 5H_2_O, 0.00011% (NH_4_)_6_Mo_7_O_24_ × 4H_2_O, 0.005% Na_4_EDTA and 1.5% agar) at 37°C. *P. crustosum* and *P. olsonii* strains were grown on PDA medium at 28°C. For the confrontation assay, *A. alternata*, *A. nidulans*, *P. crustosum*, and *P. olsonii* strains were grown on mCDB medium at room temperature. Oligonucleotides and plasmids are listed in Tables S3 and S4, respectively.

### 5.2 CRISPR‐Cas9 Plasmids Construction for Gene Deletion

To design the deletion constructs, we followed the strategy described previously [16]. Briefly, two different protospacer sequences per gene, generating two different sgRNAs, were inserted into the linearized pFC332 vector in a NEBuilder reaction [16].

### 5.3 Complementation of *atbA‐E* in *A. alternata* Mutants

The *atbA‐E* gene sequences containing 1 kb upstream of ORF (promoter), ORF and 1 kb downstream of the ORF (terminator), were amplified by PCR using *A. alternata* genomic DNA as a template, respectively. The PCR fragments were cloned into the linearized vector pJet‐*hph* (hygromycin B phosphotransferase gene) in a NEBuilder reaction, respectively.

### 5.4 Complementation of *B. maydis atbA* in *A. alternata ΔatbA* Strain

The *B. maydis atbA* gene sequences containing 1 kb upstream of ORF (promoter), the ORF, and 1 kb downstream of ORF (terminator), were amplified by PCR using *B. maydis* genomic DNA as template. The PCR fragment was cloned into the linearized vector pJet‐*hph* in a NEBuilder reaction.

### 5.5 AtbB‐, AtbC‐, AtbD‐, AtbE‐, AtbF‐, AtbG‐ and AtbH‐Tagging Plasmid Construction

The construction of AtbB‐, AtbC‐, AtbD‐, AtbE‐, AtbF‐, AtbG, and AtbH‐tagging plasmids was based on the strategy tagging laccases [16]. GA5‐*mCherry‐trpC*(*t*) fragment was amplified from the plasmid pJG43, followed by cloning into the linearized vector pJet‐*hph*‐*atbA*. The *atbB*(*p*)‐*atbB*, *atbD*(*p*)‐*atbD*, *atbE*(*p*)‐*atbE, atbG*(*p*)‐*atbG* and *atbH*(*p*)‐*atbH* (AtbB‐, AtbD‐, AtbE‐, AtbG‐, and AtbH‐encoding gene promoters and ORF sequences) fragments were amplified using *A. alternata* genomic DNA as the template, respectively. The PCR fragments were cloned into the linearized vector pJet‐*hph*‐*atbA*‐GA5‐*mCherry‐trpC(t)* in a NEBuilder reaction, respectively. The *atbC*(*p*) (promoter), *atbF* (*p*) (promoter), *atbC*‐*atbC* (*t*) (AtbC‐encoding ORF and terminator) and *atbF*‐*atbF* (*t*) (AtbF‐encoding ORF and terminator) were amplified using *A. alternata* genomic DNA as the template, respectively. *mCherry*‐GA5 fragment was amplified from the plasmid pJG43. The chimeric fragment *atbC*(*p*)‐*mCherry*‐GA5‐*atbC*‐*atbC*(*t*) was obtained by the combination of *atbC*(*p*), *mCherry*‐GA5, and *atbC*‐*atbC* (*t*) fragments in a fusion‐cloning step. The chimeric fragment *atbF*(*p*)‐*mCherry*‐GA5‐*atbF*‐*atbF* (*t*) was obtained by the combination of *atbF*(*p*), *mCherry*‐GA5 and *atbF*‐*atbF* (*t*) fragments in a fusion‐cloning step. The two chimeric fragments were cloned into the linearized vector pJet‐*hph*‐*atbA*, respectively.

### 5.6 *atb* Gene Cluster Plasmid Construction

*atbA‐atbB‐atbC* (AtbA‐, AtbB‐, and AtbC‐encoding genes) and *atbD‐atbE‐atbF* (AtbD‐, AtbE‐, and AtbF‐encoding genes) fragments were amplified using *A. alternata* genomic DNA as a template. The two fragments were cloned into the linearized vector pJet‐*hph*.

### 5.7 Plasmids Construction for Site‐Directed Mutagenesis of AtbE

Site‐directed mutation of AtbE was performed via introducing nucleotide mutations into CDS sequences of *atbE* by PCR. In detail, the *atbE* gene sequences containing 1 kb upstream of the ORF (promoter region), ORF and 1 kb downstream of ORF (terminator region) were split into two parts based on the location of mutant site. Nucleotide mutations were introduced into primers. Afterwards, the two fragments were amplified using *A. alternata* genomic DNA as template and cloned into the linearized vector pJet‐*hph* in a NEBuilder reaction.

### 5.8 Plasmid Constructions for Overexpression of *atbC* and *atbE* in the *A. alternata ΔpksA/I* Strain

To overexpress *atbC* and *atbE* genes, expression of both genes was controlled by the *A. alternata gpdA* promoter. 1 kb upstream of the *gpdA* ORF (promoter region), *atbC*/*atbE* ORF and 1 kb downstream of the *atbC/atbE* ORF (terminator region) were amplified using *A. alternata* genomic DNA. Afterwards, fragments *gpdA(p)* and *atbC‐atbC(t)* or *atbE‐atbE(t)* were cloned into the linearized vector pJet‐*hph* in a NEBuilder reaction.

### 5.9 Polymerase Chain Reaction (PCR)

Polymerase chain reaction (PCR) was performed according to the manufacturer's protocols with Q5 from New England Biolabs (NEB, Frankfurt am Main, Germany). Oligonucleotide synthesis was performed by Eurofins Genomics (Ebersberg, Germany) with the final concentration of 0.2 µM in the PCR reactions. PCR reactions were run with ∼ 10 ng plasmid DNA or ∼ 100 ng *A. alternata* or *B. maydis* genomic DNA as template. dNTPs were purchased from Roth (Karlsruhe, Germany) and used at final concentrations of 200 µM in the PCR reactions. The PCR program included a pre‐denaturation step for 3 min at 98°C, followed by 35 cycles with: a denaturation step for 20 s at 98°C; an annealing step at 63°C for 20 s for 5 cycles, an annealing step at 61°C for 20 s for 5 cycles, followed by 25 cycles with an annealing temperature of 59°C for 20 s. The elongation step was performed at 72°C with times according to the length of the amplified fragments (2 kb/min).

### 5.10 Transformation of *A. alternata* and *B. maydis*

Fresh spores were harvested from a mCDB culture plate and inoculated into 100 mL of mCDB medium at 28°C and 180 rpm for overnight cultivation. The mycelia were harvested by filtering with two layers of Miracloth (Millipore), washed once with 0.7 M NaCl solution and then digested in a Kitalase (Wako Chemicals) suspension (150 mg in 15 mL of 0.7 M NaCl solution) at 100 rpm and 30°C for 1 h. The quality and quantity of protoplasts were checked with microscopy. Protoplasts were separated from cell fragments by filtering through two layers of Miracloth and precipitated at 2,500 rpm (1,201 × g) for 10 min at 4°C. The Kitalase solution was discarded, and the protoplasts were washed once with ice‐cold 0.7 M NaCl solution by repeating the centrifugation step. Then the protoplasts were resuspended in 200 µl of STC solution (1 M sorbitol, 50 mM CaCl_2_, 50 mM Tris‐HCl, pH 8.0). 10 µg of plasmid DNA was added to the protoplasts followed by a 10 min incubation on ice. DNA uptake was induced with a heat shock at 42°C for 5 min, and after a 10 min incubation step on ice, 1 mL of 40% (w/v) polyethylene glycol 4000 (PEG 4000) solution (dissolved in STC solution) was added to the protoplasts, followed by a 20 min incubation at room temperature. The suspension was mixed with 50 mL of hand‐warm regeneration medium (34.3% saccharose, 0.5% yeast extract, 0.5% Casein hydrolysate, 0.75% agar) in a 50 mL centrifugation tube and split into a Ø 15 cm Petri dish. After overnight incubation at 28°C, the transformation plate was overlayed with 40 mL of hand‐warm regeneration medium containing HygB (80 µg/mL) and again incubated at 28°C until colonies were formed.

### 5.11 Transformation of *A. brassicicola*

*A. brassicicola* transformation was carried out following the protocol published previously. Fresh spores were harvested from a mCDB culture plate and inoculated into 100 mL of GYEB (1% glucose, 0.5% yeast extract) at 25°C and 100 rpm for 36 h. The mycelia were harvested by filtering with two layers of Miracloth (Millipore), washed once with 0.7 M NaCl solution and then digested in a Kitalase (Wako Chemicals) suspension (60 mg in 6 mL of 0.7 M NaCl solution) at 110 rpm and 30°C for 3–4 h. The quality and quantity of protoplasts were checked with microscopy. Protoplasts were separated from cell fragments by filtering through two layers of Miracloth and precipitated at 2,500 rpm (1,201 × g) for 10 min at 4°C. The Kitalase solution was discarded, and the protoplasts were washed twice with ice‐cold 0.7 M NaCl solution and once with STC solution (1 M sorbitol, 50 mM CaCl_2_, 50 mM Tris‐HCl, pH 8.0) by repeating the centrifugation step. Then the protoplasts were resuspended in 70 µL of STC solution.10 µg of plasmid DNA were added to the protoplasts, followed by a 30 min incubation on ice. DNA uptake was induced with a heat shock at 42°C for 5 min, and 800 µL of 40% (w/v) polyethylene glycol 4000 (PEG 4000) solution (dissolved in STC solution) was added to the protoplasts, followed by a 20 min incubation at room temperature. The suspension was mixed with 50 mL of hand‐warm regeneration medium (34.3% saccharose, 0.5% yeast extract, 0.5% Casein hydrolysate, 1% agar) in a 50 mL centrifugation tube and split into a Ø 15 cm Petri dish. After overnight incubation at 28°C, the transformation plate was overlayed with 40 mL of hand‐warm PDA medium containing HygB (30 µg/mL) and again incubated at 28°C until colonies were formed.

### 5.12 Microscopy

For microscopic analysis, *A. alternata* strains were grown on mCDB medium for 2 days at 28°C. A slice of agar containing spores, aerial hypha, and substrate hypha was obtained by trimming the *A. alternata* colony using a razor blade. Then it was placed on a microscope slide and covered with a coverslip. Conventional fluorescence images were captured using an EC Plan‐Neofluar 100×/1.3 Oil or 40×/0.75 objective with an AxioImager Z.1 and AxioCamMR (Zeiss).

### 5.13 Feeding 1,8‐DHN to *A. alternata* Strains

*A. alternata* strains were inoculated into 25 mL of mCDB liquid medium at 28°C and 180 rpm for 2d. Afterwards, 1,8‐DHN dissolved in DMSO was added to the medium to the final concentration of 0.03 mg/mL. Then, *A. alternata* strains were grown for one more day at the same condition.

### 5.14 Secondary Metabolites Extraction

To extract secondary metabolites from liquid medium, *A. alternata* strains were grown on mCDB medium or mCDB medium plus tomato puree (400 g/liter) for 60 h at 28°C. 1 mL of medium from each sample was pipetted into a 2 mL microtube and extracted with 1 mL of ethyl acetate by shaking for 10 min. After centrifugation for 1 min at 13 000 rpm (15.700 × g), 800 µL of upper organic phase containing secondary metabolites per sample was token into a 1.5 mL microtube and dried in a centrifugal vacuum concentrator (Thermo Scientific). Each sample was dissolved again using 80 µL of methanol for further TLC or HPLC assay.

To extract secondary metabolites from solid medium, *A. alternata*, *A. brassicicola*, *A. nidulans*, *B. maydis*, *P. crustosum*, and *P. olsonii* strains were grown on mCDB medium for 2–5 days at 28°C or room temperature. The whole culture of each plate was collected into a 50 mL centrifugation tube and homogenized in 20 mL of ethyl acetate with an ultra‐turrax homogenizer (IKA T10). After centrifugation for 5 min at 2,500 rpm (1,201 × g), 2 mL of the upper organic phase containing secondary metabolites per sample was taken into a 2 mL microtube and dried in a vacuum centrifuge (Thermo Scientific). Each sample was dissolved again using 500 µL of methanol for further TLC or HPLC assay.

### 5.15 Thin Layer Chromatography (TLC) Analysis

For the qualitative analysis of the *A. alternata* and *A. brassicicola* extracts, 10 µL of crude extract was put on TLC plates coated with 0.25 mm silica gel as stationary phase (Pre‐coated TLC plates SIL G‐25, Macherey‐Nagel, Düren, Germany). The mobile phase for separation of the metabolites consisted of 50% of toluol, 40% ethyl acetate, and 10% formic acid. The secondary metabolites were visualized using UV light at 254 nm.

### 5.16 High‐Performance Liquid Chromatography (HPLC) Analysis

HPLC analysis was conducted with the Chromeleon 7.3.1 Chromatography Data System (Thermo Scientific) using a Hypersil GOLD PFP column (150 × 4.6 mm, 3 µm particle size, Thermo Scientific). Solvent A was water with 0.1% (v/v) of formic acid, and solvent B was acetonitrile with 0.1% (v/v) of formic acid. A linear gradient with a flow rate of 1 mL/min was applied. The gradient was as follows: 0 min: 5% B/95% A, 0–15 min: 5% B/95% A to 100% B, 15–20 min: 100% B, 20–23 min: 100% B to 5% B/95% A. UV absorptions at 254 nm were illustrated.

### 5.17 Secondary Metabolite Purification

A semi‐preparative Agilent 1200 HPLC system equipped with an Agilent Eclipse XDB‐C18 (9.4 × 250 mm, 5 µm) column (Agilent Technologies, Böblingen, Germany) was then used for metabolite isolation. 1.0 mg of **2** (t_R_ = 86.8 min) was purified by using an isocratic elution profile with acetonitrile/water (28:72, v/v) as solvents within 100 min. 5.0 mg of **3** (t*_R_* = 36.9 min), 4.8 mg of **4** (t*_R_* = 38.8 min) and 1.0 mg of **5** (t*_R_* = 44.6 min) were isolated by using a linear gradient of acetonitrile in water from 40% to 65% within 50 min. 4.0 mg of **6** (t*_R_* = 9.0 min) and 9.0 mg of **7** (t*_R_* = 31.0 min) were obtained by using a linear gradient from 20% to 60% within 40 min. 3.0 mg of **8** (t*_R_* = 11.1 min), 5.0 mg of **10** (t*_R_* = 20.5 min) and 8.0 mg of **9** (t*_R_* = 22.4 min) were isolated by using a linear gradient from 25% to 45% within 30 min. The integrity of the structures was confirmed by comparison of their NMR data with those published previously. For the compound described in Figure 6D, preparative HPLC was conducted on an Agilent system with a VDSpher PUR 100 C18‐M‐SE column (150 × 20 mm, 5 µm) for isolation. 2.0 mg of the compound (t*_R_* = 36.0 min) was isolated using a linear gradient from 30% to 80% methanol in water within 40 min at 10.0 mL/min (Supporting Information).

### 5.18 RNA Extraction and qRT PCR

1×10^5^ fresh spores were incubated using 10 mL mCDB medium or mCDB medium containing tomato puree (400 g/liter) in a Ø 5 cm Petri dish at 28°C for 60 h. The mycelia were collected and frozen immediately in liquid nitrogen, followed by being ground into powder using liquid nitrogen. Then, total RNA was isolated using TRIzol reagent according to the standard protocol. RNA samples were quantified and treated with the TURBO DNA‐free kit (Invitrogen) to remove DNA contamination. qRT PCR was performed as described [16].

### 5.19 Transcriptome Sequencing and Analyses

*A. alternata* strains were incubated using 10 mL mCDB medium or mCDB medium containing tomato puree (400 g/liter) in a Ø 5 cm Petri dish at 28°C for 60 h before RNA isolation. Samples of total mRNA were subjected to RNA sequencing by BGI TECH SOLUTIONS (HONGKONG) CO., LIMITED. After read filtering, the clean reads were mapped to the reference genome *A. alternata* ATCC66981 using HISAT2 [45]. For each transcript, a fragment per kilobase of transcript per million mapped reads value was calculated to quantify its expression abundance and variations, using StringTie v1.3.1. DEGs were identified using DEseq2 with thresholds of |log2(fold change)| ≥1 and FDR <0.05. Potential functions of these genes were assigned based on their interpro classification (https://www.ebi.ac.uk/interpro/).

### 5.20 NMR Analysis

NMR spectra were recorded on a JEOL ECA‐500 MHz spectrometer (JEOL Ltd., Tokyo, Japan) and analyzed with MestReNova 9.0.0 (MestreNova, Santiago de Compostela, Spain). The samples were dissolved in (CD_3_)_2_CO, CDCl_3_, DMSO‐*d_6_* or CD_3_OD. The data obtained were compared with those in the literature.

### 5.21 Genome Sequencing and Annotation

The genome of *A. brassicicola* DSM62008 was sequenced with a Nanopore 10.4 flowcell, and base called with dorado. Reads were assembled with canu v2.2 [46], with default settings and genomeSize = 38 m. After softmasking repetitive elements with EarlGrey [47], the resulting assembly was annotated with the funannotate pipeline (https://doi.org/10.5281/zenodo.1134477). To improve transcript prediction, we included CDS from *A. alternata* Altal1 (NCBI: GCF_001642055.1), which were mapped to the genome with minimap2 [48] prior to gene prediction with Augustus [49]. Functional annotation was added using iprscan [50], and secondary metabolite clusters were predicted with antiSMASH [51].

### 5.22 BGC Annotation and Phylogenetic Inferences

An initial survey of species containing the *atb* and *ctb* BGCs was performed in cblaster [52]. For representative species containing any of the BGCs, the reference genomes and proteomes were downloaded from NCBI GenBank and a blastp search with all queries shown in Figure S10 was performed (blast + v2.14.1; ‐evalue 10 ‐outfmt 6 ‐seg yes ‐soft_masking true ‐max_target_seqs 500). The results were filtered for hits with a minimum length of two‐thirds of the query sequence, and then the hit with the highest pident (percent identity to the query) was selected. The corresponding phylogenetic tree was inferred from the genomes. BUSCO (v 5.4.6 with lineage set fungi_odb10) was used to identify 437 BUSCOs present across all genomes. The BUSCOs were combined by locus and aligned using mafft (v7.505) with default settings. The phylogeny was inferred using iqtree2 (v 2.2.0.3) with default settings. For gene trees, collections of sequences were treated the same as for the species phylogeny.

## Author Contributions

**Jia Gao**: investigation, methodology, validation, formal analysis. **Leyao Chen**: methodology, investigation. **Karin Steffen**: methodology, investigation. **Henrik Schweder**: methodology, investigation. **Birgit Schreckenberger**: methodology. **Shu‐Ming Li**: validation, supervision. **Ben Auxier**: methodology, investigation, validation. **Antonis Rokas**: supervision. **Reinhard Fischer**: supervision, resources, project administration, validation, visualization, writing – review and editing, funding acquisition, conceptualization.

## Conflicts of Interest

A.R. is a scientific consultant for LifeMine Therapeutics, Inc. The authors declare no conflicts of interest.

## Supporting information



Supplementary figures and compound characterization data including spectra were included. **Supporting File 1**: anie74465‐sup‐0001‐SuppMat.pdf.

**Supporting File 2**: anie74465‐sup‐0002‐TableS1‐S4.zip.

**Supporting File 3**: anie74465‐sup‐0003‐VideoS1‐S3.zip.

## Data Availability

Annotated genome assembly as well as raw genomic data for *A. brassicicola* can be found under NCBI BioProject PRJNA1251268.
