# Supplementary material for: Intermolecular Carbon–Carbon Naphthalene Coupling Involves a Radical Mechanism Driven by Cytochrome P450 in Alternaria alternata
Source: Angew Chem Int Ed Engl. 2026 Sep 18;65(46):e2505284. doi: 10.1002/anie.2505284 (PMC13749262; doi:10.1002/anie.2505284)
Supplement: Supplementary file 1 [file ANIE-65-e2505284-s002.pdf]

Supporting Information

The authors have cited additional references within the Supporting Information.[53-61]

Intermolecular Carbon-Carbon Naphthalene Coupling Involves a Radical Mechanism Driven by P450 Cytochrome in *Alternaria alternata*

Jia Gao[1], Leyao Chen[2], Karin Steffen[3,4], Birgit Schreckenberger[1], Shu-Ming Li[2], Ben Auxier[5], Antonis Rokas[3,4] and Reinhard Fischer[1]*

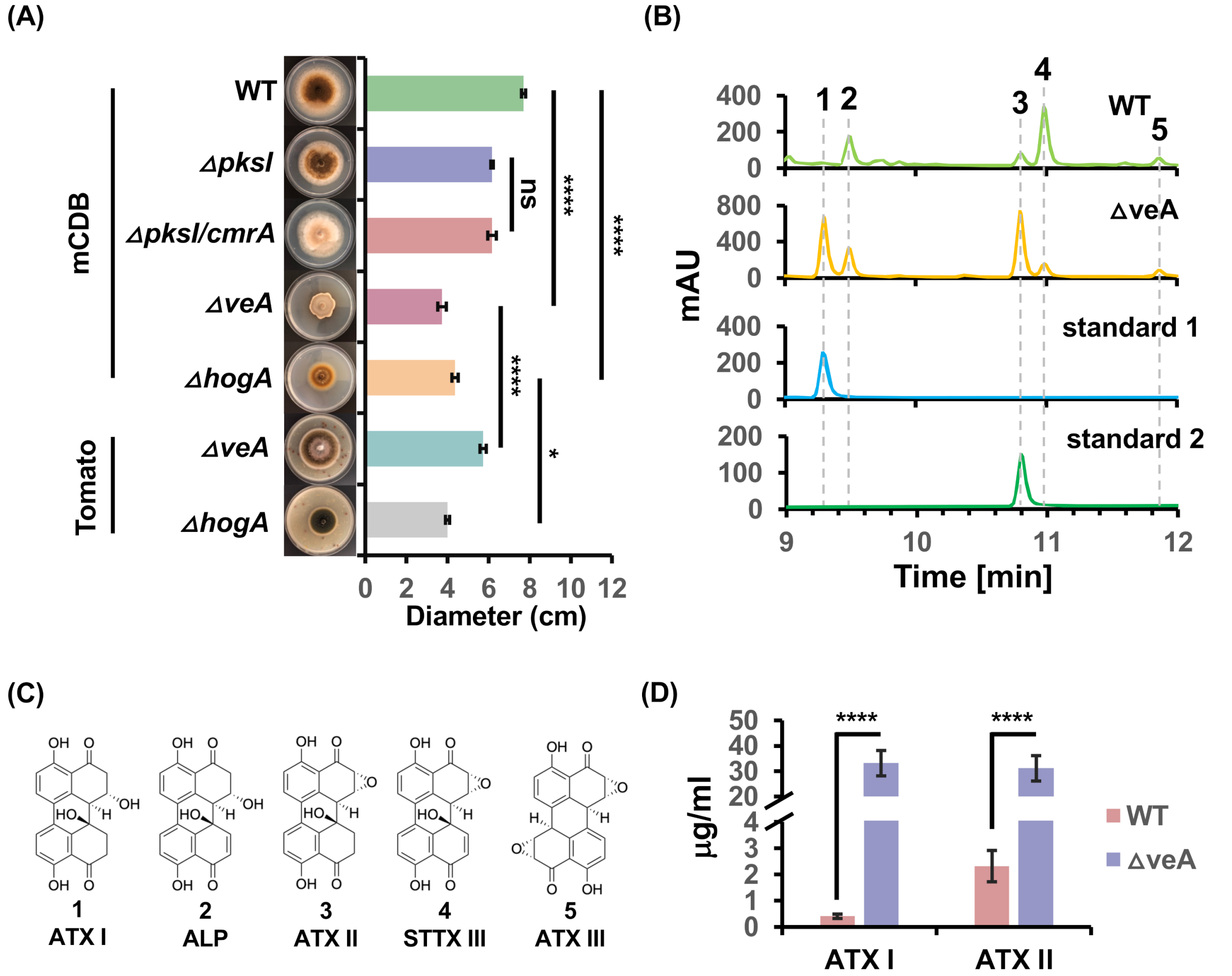

Figure S1. Perylenequinones (PQs) formation in *A. alternata* strains. (A) The growth of different *A. alternata* WT and different mutant strains grown on solid mCDB medium or mCDB plus tomato puree (400 g/liter) medium for 5d at 28 °C. (**B**) High Performance Liquid Chromatography assay at 254 nm of extracts from *A. alternata* WT and *ΔveA* strains grown on liquid mCDB medium for 60h at 28 °C. Compounds **1**-**5**: altertoxin I, alterperylenol, altertoxin II, stemphytotoxin III and altertoxin III; standard 1: altertoxin I; standard 2: altertoxin II. (**C**) Chemical structures of altertoxin I-III (ATX I-III), alterperylenol (ALP) and stemphytotoxin III (STTX III). (**D**) Quantification of ATX I and II produced by *A. alternata* WT and *ΔveA* strains grown on liquid mCDB medium for 60h at 28 °C.

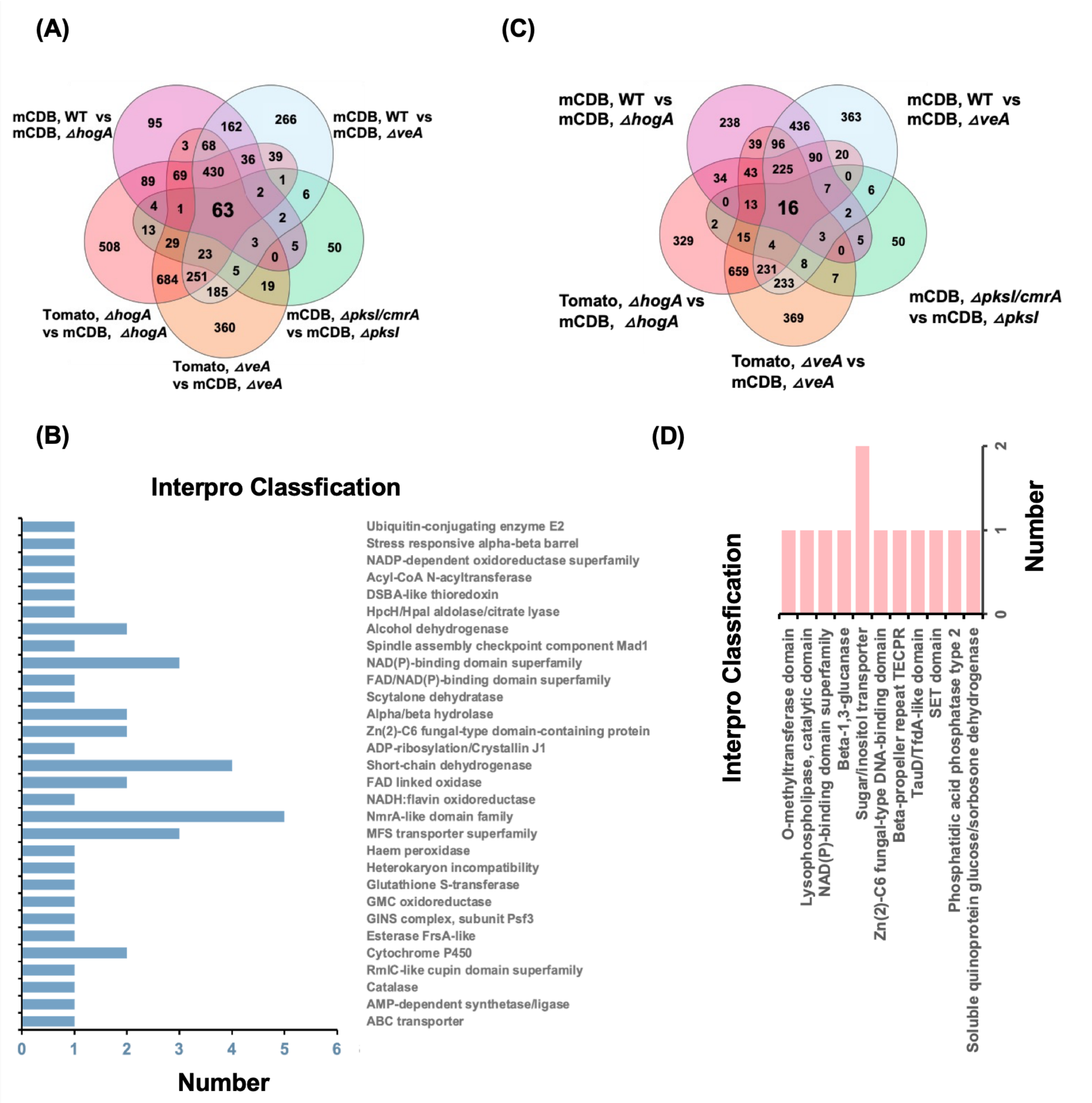

Figure S2. Genome-wide expression analyses to identify the gene encoding the enzyme dimerizing naphthalene to PQs. (**A**) Venn diagrams of the up-regulated differentially expressed genes (DEGs) identified among WT and different mutant strains grown on liquid mCDB medium or mCDB plus tomato puree (400 g/liter) medium for 60h at 28 °C. $|\log2(\text{fold change})| \geq 1$; $p<0.05$. **(B)** The function of proteins encoded by the common up-regulated 63 DEGs was predicted based on Interpro Classification. **(C)** Venn diagrams of the down-regulated differentially expressed genes (DEGs) identified among WT and different mutant strains grown on liquid mCDB medium or mCDB plus tomato puree (400 g/liter) medium for 60h at 28 °C. $|\log2(\text{fold change})| \geq 1$; $p<0.05$. **(D)** The function of proteins encoded by the common down-regulated 16 DEGs was predicted based on Interpro Classification.

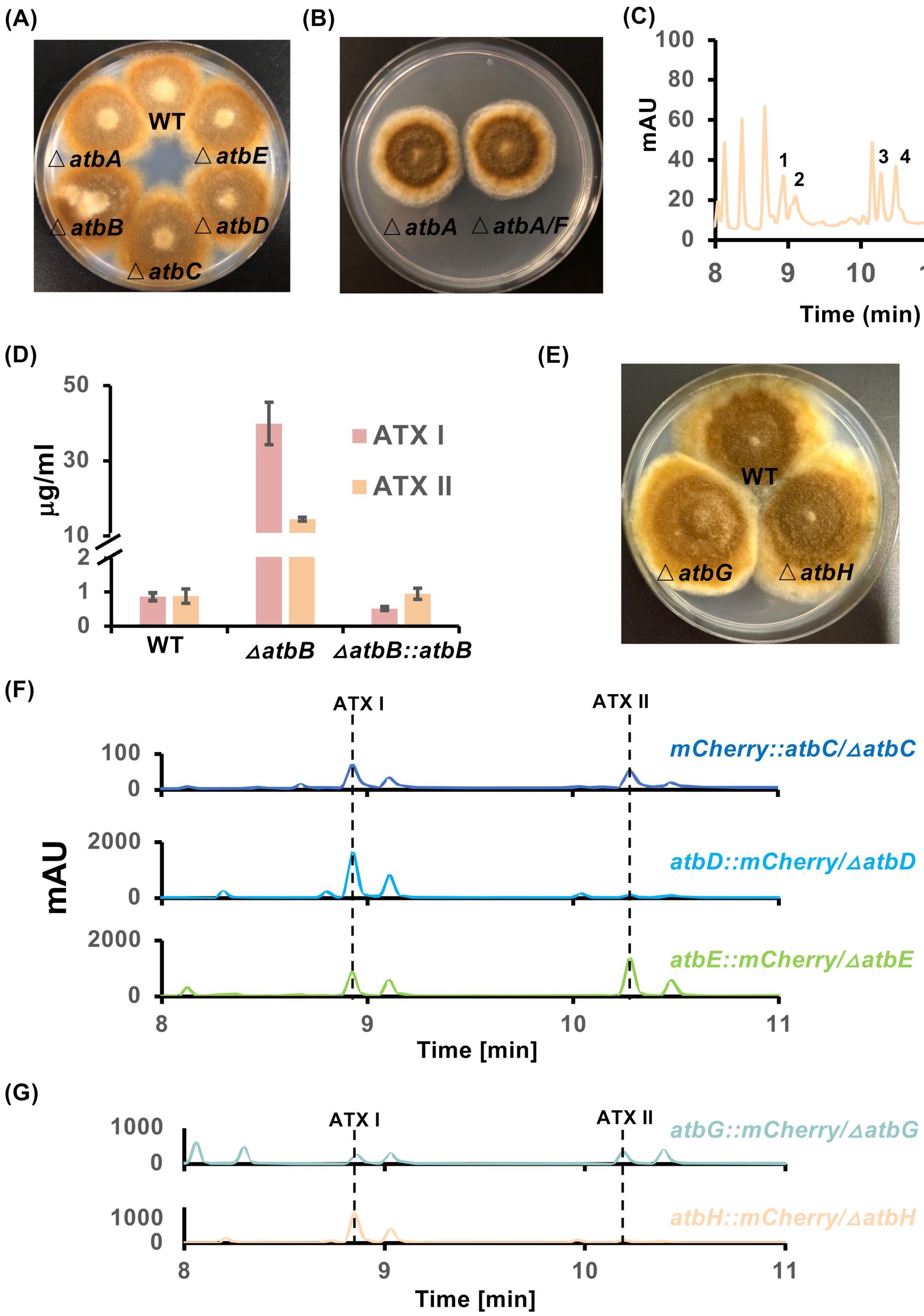

Figure S3. The *atb*-gene cluster is required for PQ biosynthesis. The absence of AtbA-F has no significant influence on growth and DHN-melanin production. (A) Growth of WT and different mutant strains on mCDB medium for 5d at 28 °C. **(B)** Growth of *ΔatbA* and *ΔatbA*/F strains on mCDB medium for 5d at 28 °C. (**C**) HPLC chromatograms at 254 nm of extracts from WT grown on regeneration medium for 3d at 28 °C. Compounds **1**-**5**: altertoxin I, alterperylenol, altertoxin II, stemphytotoxin III and altertoxin III. **(D)** Quantification of ATX I and II produced by WT, *ΔatbB* and the re-complemented strain *ΔatbB::atbB* grown on mCDB medium for 3d at 28 °C. **(E)** Growth of WT, *ΔatbG* and *ΔatbH* strains on mCDB medium for 5d at 28 °C. **(F)** mCherry-AtbC, AtbD-mCherry and AtbE-mCherry rescue the production of PQs in *ΔatbC*, *ΔatbD* and *ΔatbE* strains. ATX I and II are indicated by dash lines. (**G**) AtbG-mCherry and AtbH-mCherry rescue the production of PQs in *ΔatbG* and *ΔatbH* strains. ATX I and II are indicated by dash lines.

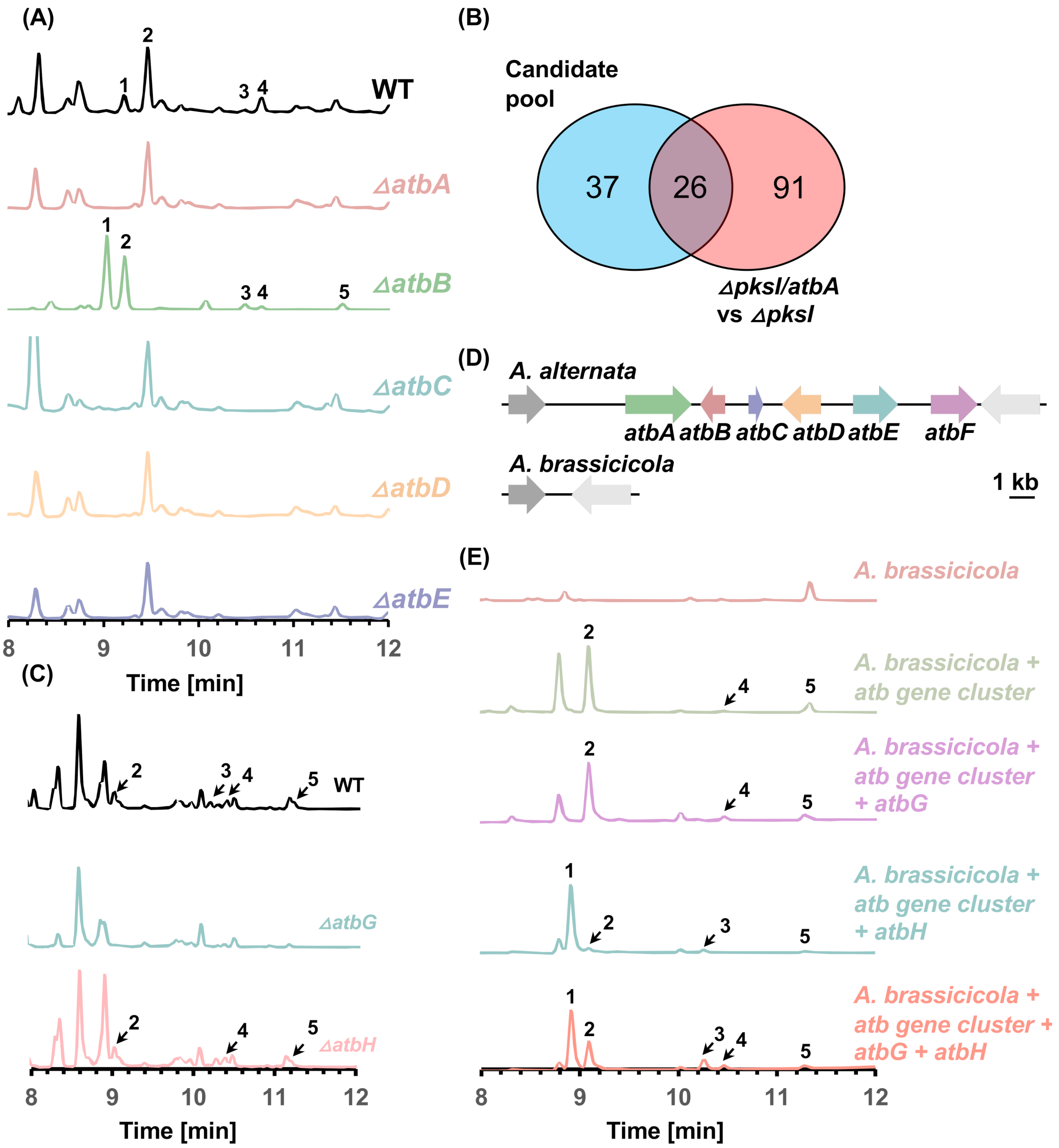

Figure S4. *atb* gene cluster plus *atbG* and *atbH* scattered in the genome are required for the biosynthesis of PQs in *A. alternata*. (**A**) HPLC chromatograms at 254 nm of extracts from WT and each mutant strain (*ΔatbA*, *ΔatbB*, *ΔatbC*, *ΔatbD* and *ΔatbE*) grown mCDB medium for 5d at 28 °C. Compounds 1-5: altertoxin **I**, alterperylenol, altertoxin **II**, stemphytotoxin **III** and altertoxin **III**. (**B**) Venn diagram of the up-regulated DEGs identified between candidate pool and compared group *ΔatbA/pksI* vs *ΔpksI*. |log2(fold change)| ≥1; p<0.05. *ΔatbA/pksI* and *ΔpksI* strains were grown on liquid mCDB medium for 60h at 28 °C for transcriptome assay. Candidate pool represents the 63 commonly up-regulated DEGs in Supplementary Figure2a. (**C**) HPLC chromatograms at 254 nm of extracts from WT and each mutant strain (*ΔatbG* and *ΔatbH*) grown mCDB medium for 5d at 28 °C. **(D)** Absence of the *atb*-gene cluster from the genome of *A. brassicicola*. **(E)** Reconstitution of PQ biosynthesis in *A. brassicicola*.

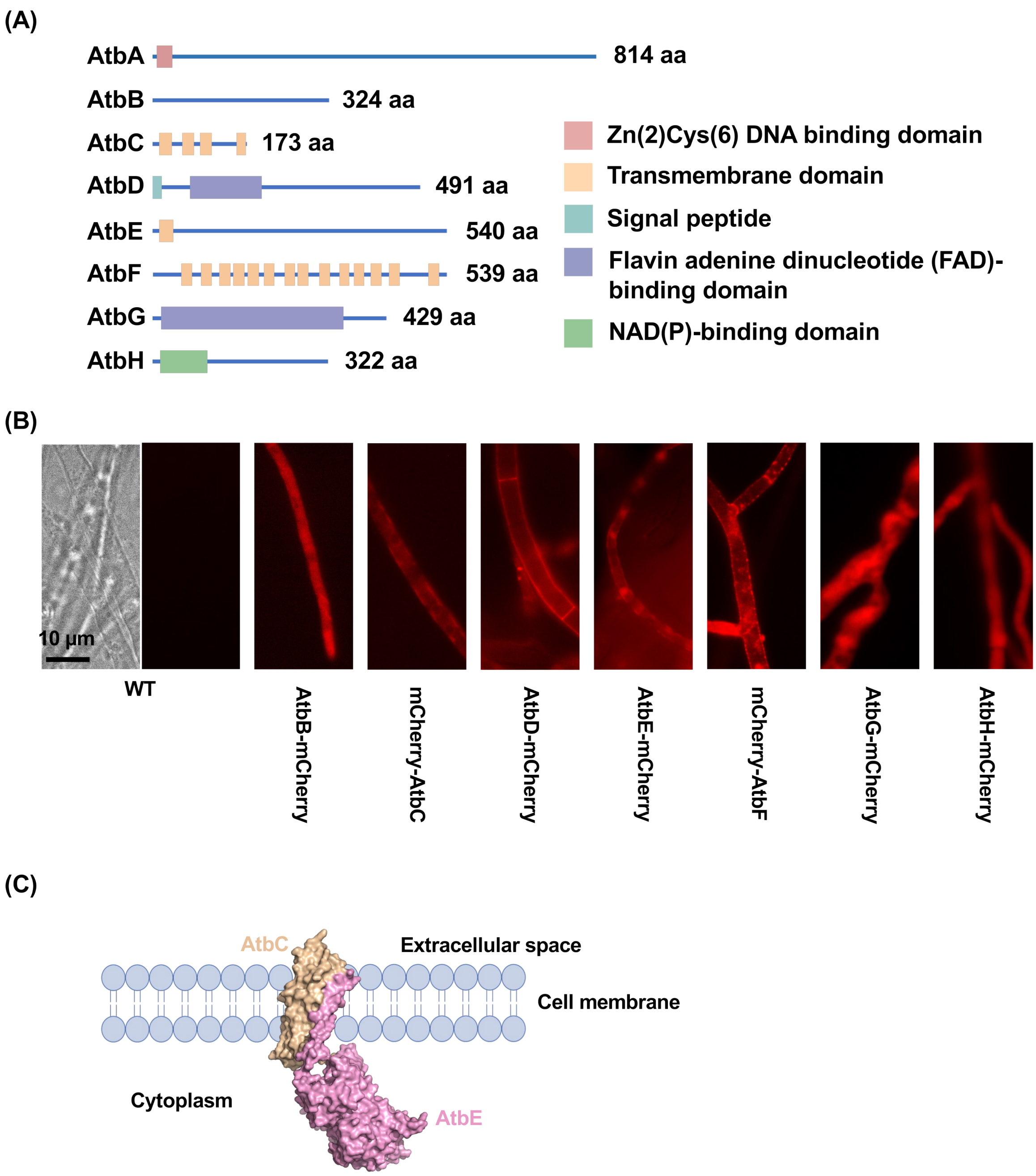

Figure S5. Localization of AtbB-H in *A. alternata*. (A) Prediction of protein domains of AtbA-H. **(B)** Subcellular localization of AtbB-H. AtbB, AtbG and AtbH are located into the cytoplasm; AtbC-F are located at the cell membrane. Scale bar: 10 µm. **(C)** AlphaFold 3 predicts interaction of AtbC and AtbE through the transmembrane domains.

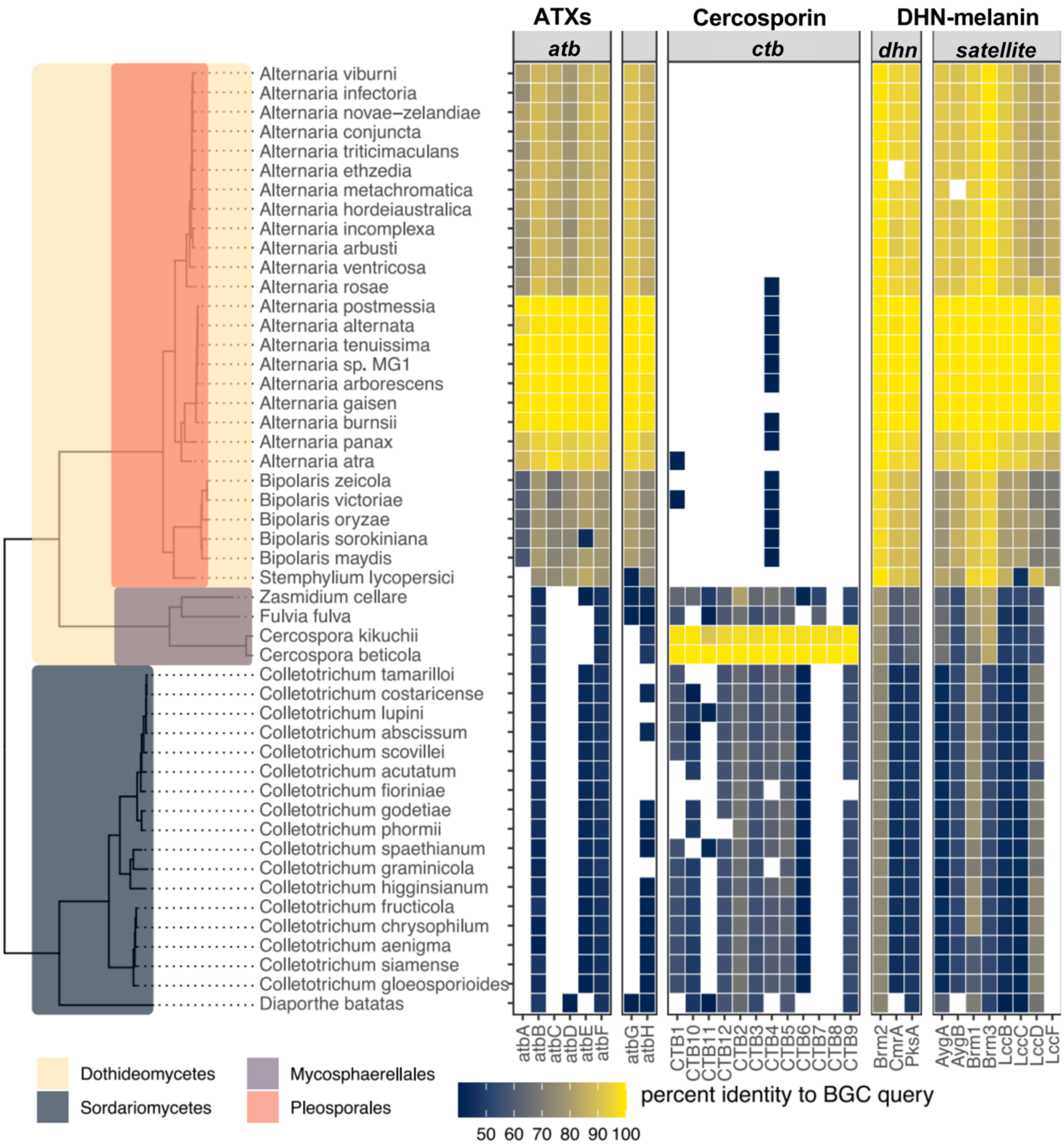

Figure S6. BGC presence across fungal genomes. The phylogenetic inference was based on the 437 single-copy orthologs (BUSCOs) found complete across all 49 species. Phylogeny of 49 species containing either the *atb* or the *ctb* BGC, as well as DHN-melanin core biosynthesis genes (*dhn*) and additional biosynthetic “satellite” genes present in those. Color scheme indicates percent identity to the respective query.

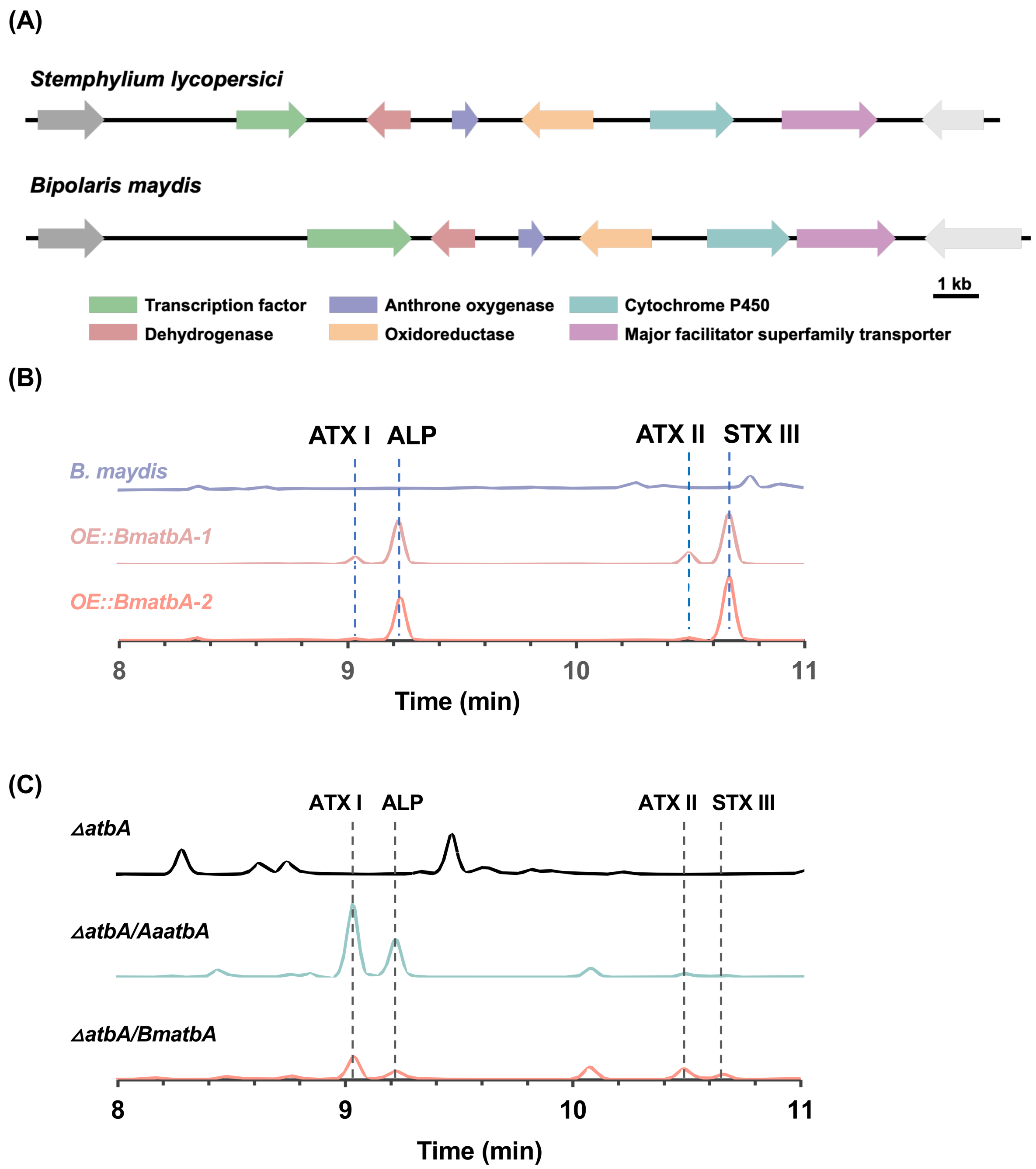

Figure S7. PQs biosynthesis in different fungi. (A) Synteny and rearrangements of *atb* gene cluster in *Stemphylium lycopersici* and *Bipolaris maydis*. **(B)** HPLC chromatograms at 254 nm of extracts from *B. maydis* WT and *BmatbA*-overexpression strains grown on mCDB medium for 3d at 28 °C. ATX I, ALP, ATX II and STTX III are indicated by dash lines. **(C)** *BmatbA* of *B. maydis* rescues the lack of *atbA* in *A. alternata*. HPLC chromatograms at 254 nm of extracts from *A. alternata ΔatbA* and *atbA* re-complemented strains, *ΔatbA/AaatbA* and *ΔatbA/BmatbA* grown on mCDB medium for 3d at 28 °C. ATX I, ALP, ATX II and STTX III are indicated by dash lines.

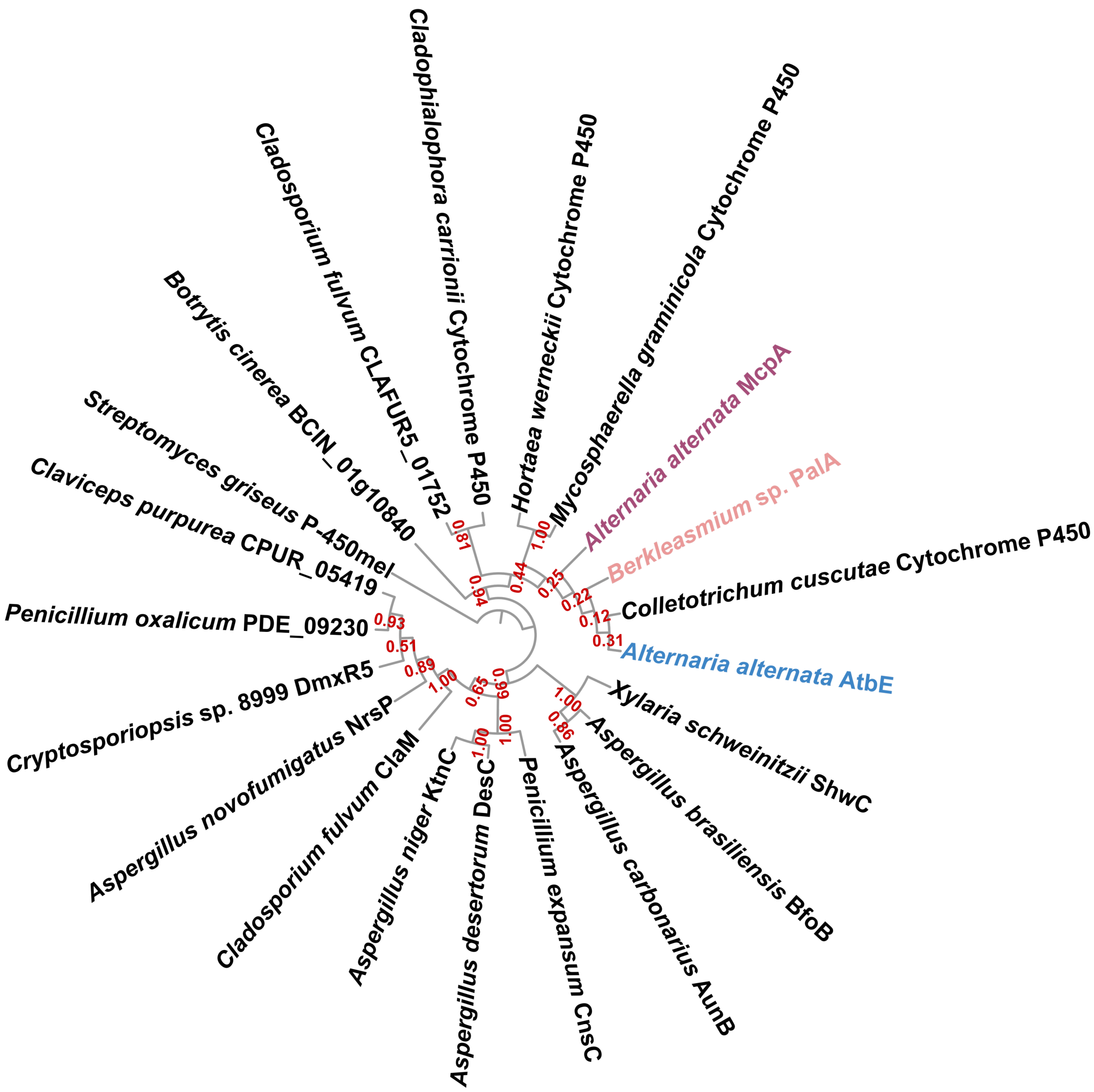

Figure S8. Identification of a new AtbE-related enzyme in *A. alternata*. AtbE was compared to other P450 enzymes identified after database searches and inclusion of P450 enzymes which catalyze also phenol coupling reactions[7]. Protein sequences were aligned using Multiple Sequence Comparison by Log-Expectation (MUSCLE) program (https://toolkit.tuebingen.mpg.de/tools/muscle). The phylogenetic tree was generated using MEGA12. The statistical method is maximum likelihood (ML). The test of phylogeny is standard bootstrap with 1000 replicates. The substitution model is WAG model. The parameter of rates among sites is Gamma Distributed, and the number of discrete gamma categories is 5. The gaps/missing data is partial deletion with 95% site coverage cutoff. The ML Heuristic method is Nearest-Neighbor-Interchange (NNI). The initial tree for ML is Make initial tree automatically without branch swap filter. The number of threads is 7. *Streptomyces griseus* P-450mel was used as outgroup. The following sequences were used: *Streptomyces griseus* P-450mel (BAE07215), *Aspergillus niger* KtnC (A2QK67), *Aspergillus desertorum* DesC (A0A0N9HKQ7), *Cladosporium fulvum* ClaM (P0CU70.1), *Cryptosporiopsis* sp. 8999 DmxR5 (QCL09096), *Claviceps purpurea* CPUR_05419 (CCE31566.1), *Penicillium oxalicum* PDE_09230 (EPS34266.1), *Aspergillus novofumigatus* NrsP (XP_024680889), *Aspergillus carbonarius* AunB (OOF95607), *Aspergillus brasiliensis* BfoB (OJJ74130), *Xylaria schweinitzii* ShwC (QLM00038.1), *Penicillium expansum* CnsC (A0A0A2JY25), *Alternaria alternata* AtbE (JGI protein ID: 110840), *Berkleasmium* sp. PalA (WYV77111.1), *Alternaria alternata* McpA (JGI protein ID: 109432), *Botrytis cinerea* BCIN_01g10840 (XP_001556428.1), *Mycosphaerella graminicola* Cytochrome P450 (XP_003852976.1), *Colletotrichum cuscutae* Cytochrome P450 (KAK1458644.1), *Cladosporium fulvum* CLAFUR5_01752 (XP_047756537.1), *Hortaea werneckii* Cytochrome P450 (KAI7344807.1), *Cladophialophora carrionii* Cytochrome P450 (OCT47350.1).

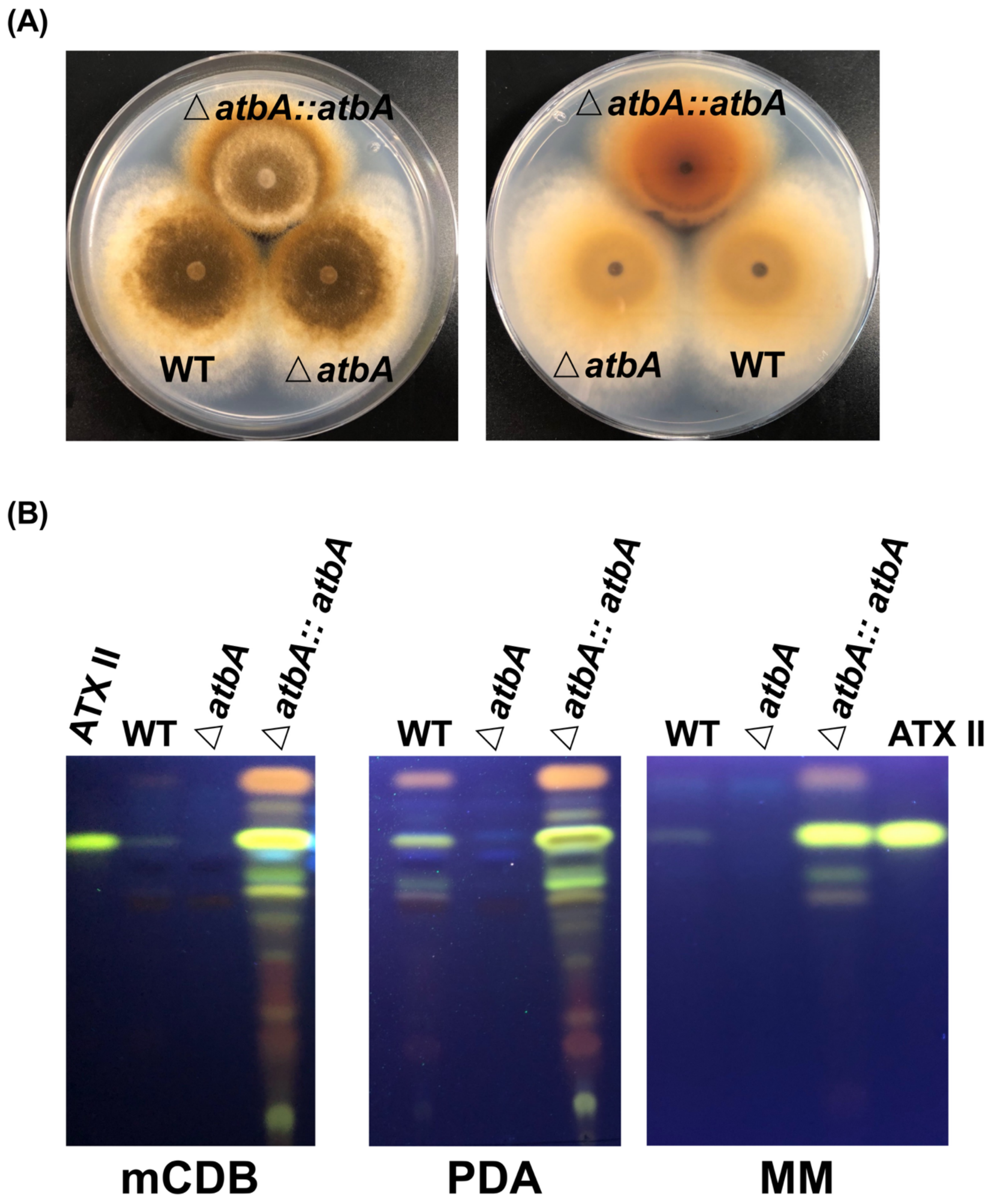

Figure S9. *atbA* re-complemented strain produces more PQs compared to WT. (A) Growth of WT, *ΔatbA* and *atbA* re-complemented strain on mCDB medium for 5d at 28 °C. **(B)** Thin layer chromatography assay of extracts from WT, *ΔatbA* and *atbA* re-complemented strain grown on mCDB, PDA and minimum medium (MM) for 3d at 28 °C. ATX II was used as standard for comparison.

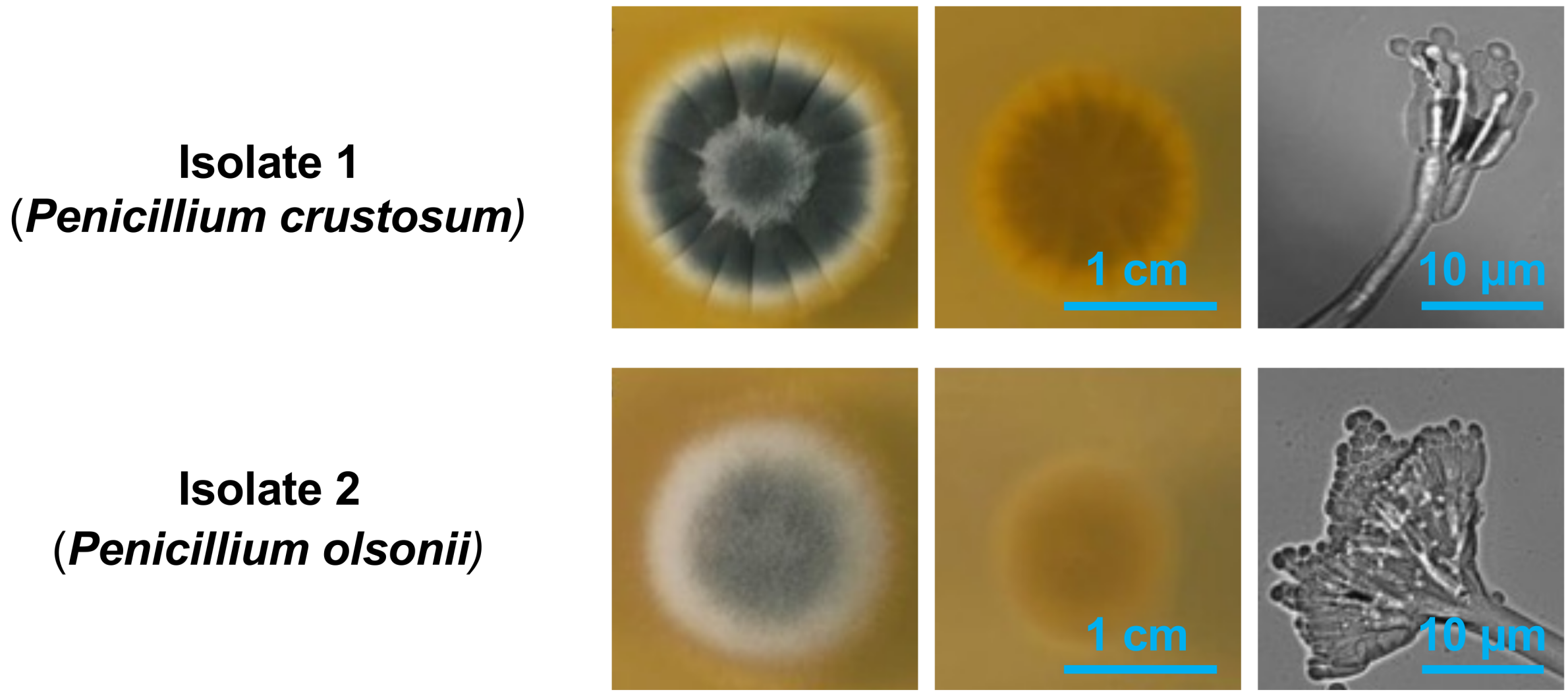

Figure S10. Physiological characterization of two fungi isolated from tomato fruit grown on PDA medium for 3d at 28 °C. Isolate 1 and 2 were identified as *Penicillium crustosum* and isolate 2 was identified as *Penicillium olsonii* based on ITS sequences amplified by the primer pair ITS1 and ITS4.

(A)

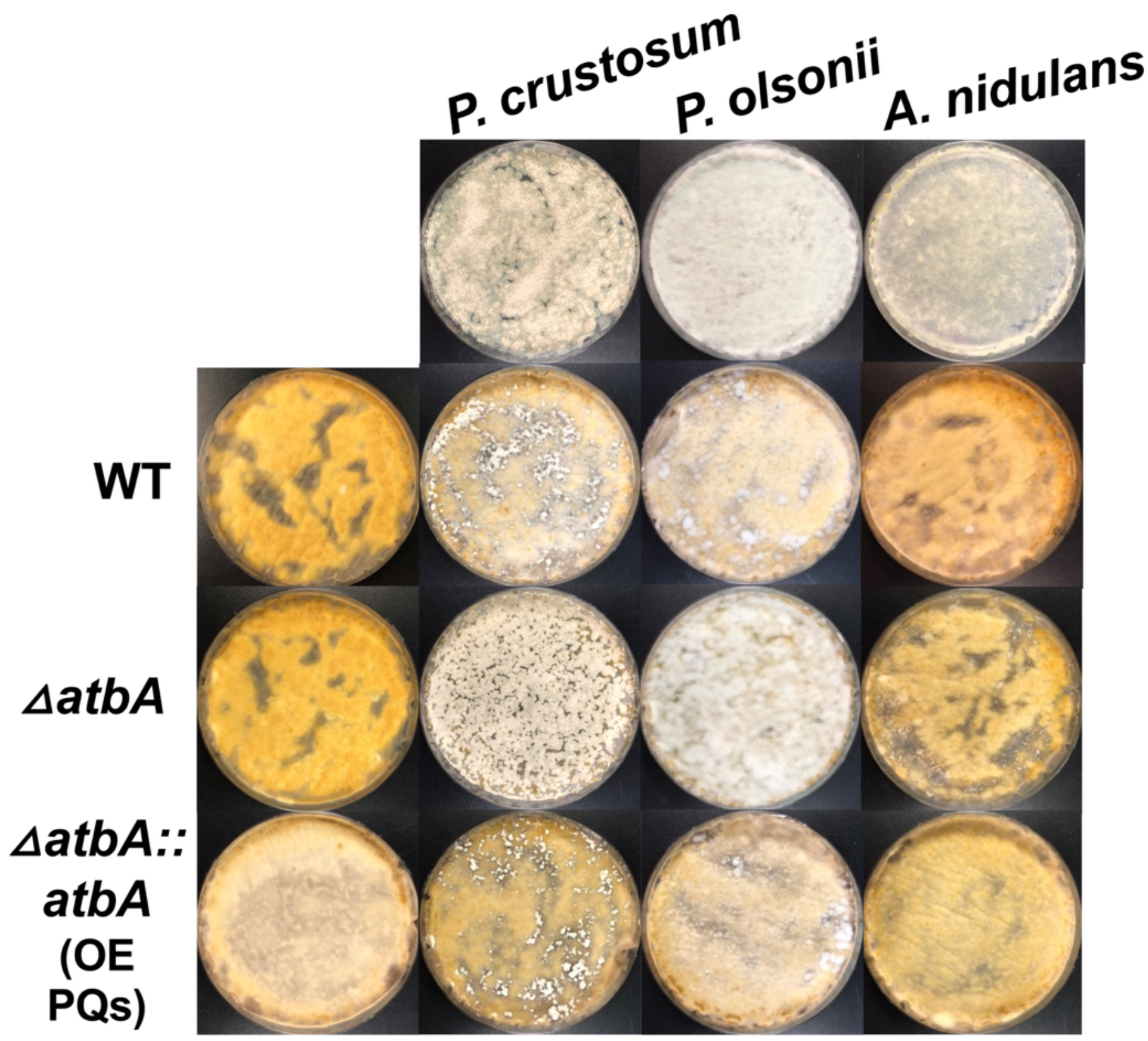

(B)

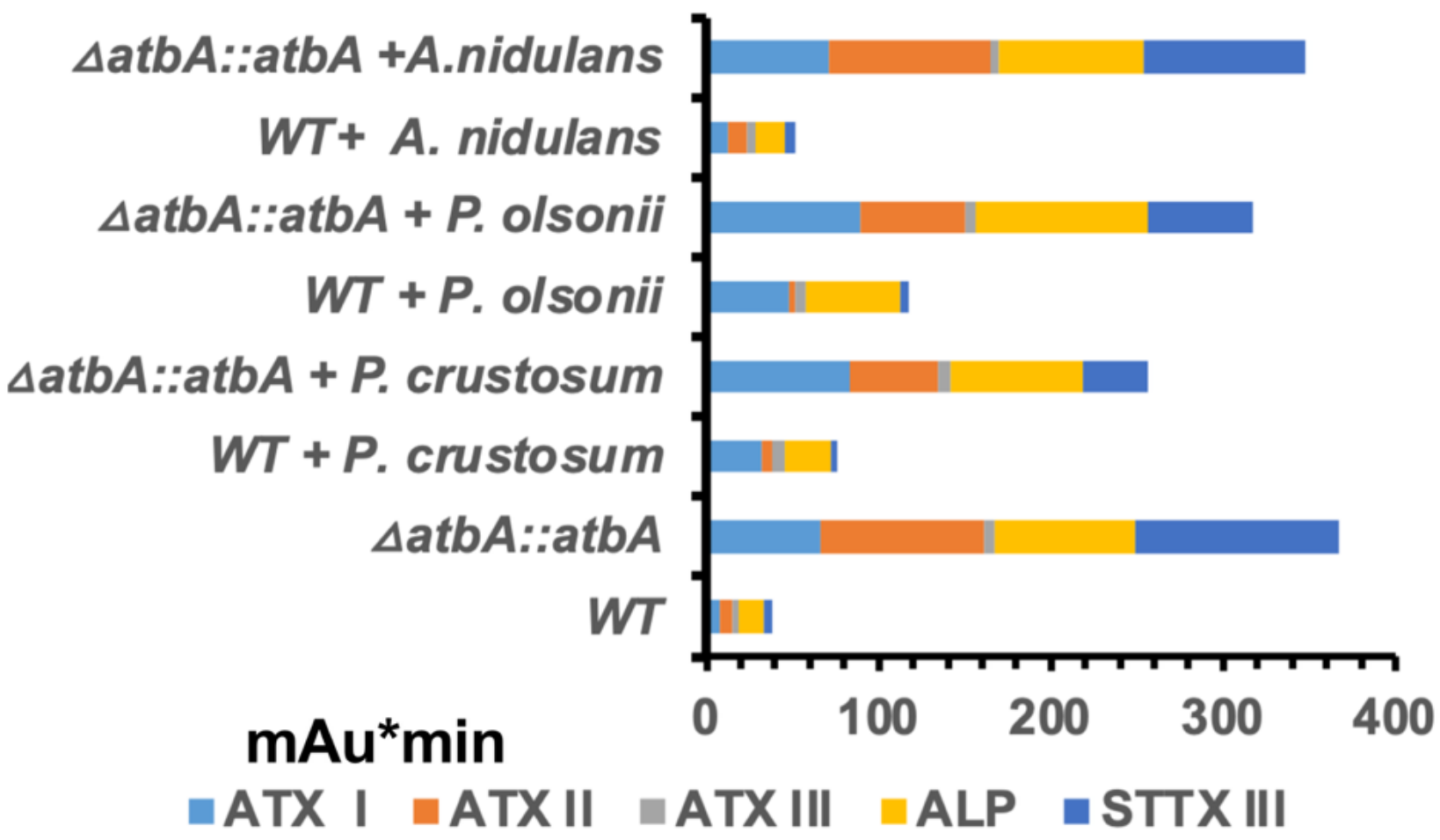

Figure S11. PQs play a role in interfungal competition. In order to test the role of PQs in interspecies competition, we used the isolates described in **Figure S7** and *A. nidulans* as a control. (**A**) Growth of *A. alternata* strains (WT, *ΔatbA* and the re-complemented strain *ΔatbA::atbA*) competing with *P. crustosum*, *P. olsonii*, *A. nidulans* on mCDB medium for 5 days at room temperature. *A. alternata* colonies appeared brownish, *P. crustosum* and *P. olsonii* are greenish or whitish, and *A. nidulans* light-yellow. The color of the fungal surface was a first hint to the proportion of the different fungi after co-cultivation. The surface of wild-type *A. alternata* grown together with *A. nidulans* resembled the surface of *A. alternata* grown alone, suggesting that *A. alternata* outcompeted *A. nidulans*. Combination of *A.*

alternata with the two *Penicillium* strains showed a light-yellow color, suggesting intermingled growth of the fungi. Combination of the ∆*atbA* strain with the *Penicillium* strains led to more white-, and the combination of the PQ-overproducing strain led to more yellow-appearing mycelial surfaces, suggesting dominance of the *Penicillium* strains or the *Alternaria* strain, respectively. (**B**) Quantification of PQs produced by *A. alternata* strains (WT, and the re-complemented strain *∆atbA::atbA*), and *A. alternata* strains competing with *P. crustosum*/*P. olsonii*/*A. nidulans* on mCDB medium for 5 days at room temperature. In total, the amounts of PQs isolated from extracts of *A. alternata* WT co-cultured with *P. crustosum*, *P. olsonii* and *A. nidulans* were 2.0-, 3.1- and 1.3-fold higher compared to that of *A. alternata* WT grown alone. The amounts of PQs from extracts of the *A. alternata atbA*-complemented strain competing with *P. crustosum*, *P. olsonii* and *A. nidulans* were slightly reduced compared to that of the *A. alternata atbA*-complemented strain grown alone. In detail, the amount of ATX I isolated from extracts of *A. alternata* WT co-cultured with *P. crustosum*, *P. olsonii* and *A. nidulans* were 5.7-, 3.8- and 1.5-fold higher compared to that of *A. alternata* WT grown alone. However, the amount of ATX II from extracts of *A. alternata* WT competing with *P. crustosum* and *P. olsonii* were slightly reduced compared to that of *A. alternata* WT grown alone. The amount of ATX II from extracts of *A. alternata* WT competing with *A. nidulans* was 1.6-fold higher compared to that of *A. alternata* WT grown alone. The amounts of ATX III isolated from extracts of *A. alternata* WT co-cultured with *P. crustosum*, *P. olsonii* and *A. nidulans* were 2.1-, 1.7- and 1.2-fold higher compared to that of *A. alternata* WT grown alone. The amounts of ALP (alterperylenol) isolated from extracts of *A. alternata* WT co-cultured with *P. crustosum*, *P. olsonii* and *A. nidulans* were 1.8-, 3.8- and 1.2-fold higher compared to that of *A. alternata* WT grown alone. No significant difference was detected on the amounts of STTX III (stemphyltoxin III). The amount of STTX III from extracts of *A. alternata* WT competing with *A. nidulans* was 1.4-fold higher compared to that of *A. alternata* WT grown alone. The amounts of ATXI from extracts of the *A. alternata atbA*-complemented strain competing with *P. crustosum*, *P. olsonii* and *A. nidulans* were slightly increased compared to that of the *A. alternata atbA*-complemented strain grown alone. However, the amounts of ATXII from extracts of the *A. alternata atbA*-complemented strain competing with *P. crustosum* and *P. olsonii* were 46.8% and 36.2% reduced compared to that of the *A. alternata atbA*-complemented strain grown alone. No significant difference in ATXII production was detected when the *A. alternata atbA*-complemented strain competed with *A. nidulans* and was grown alone. The amounts of ATX III and ALP from extract of the *A. alternata atbA*-complemented strain competing with *P. crustosum*, were slightly increased compared to that of the *A. alternata atbA*-complemented strain grown alone. No significant difference in ATX III and ALP production was detected when the *A. alternata atbA*-complemented strain competed with *P. olsonii* and *A. nidulans* was grown alone. The amounts of STTX III from extract of the *A. alternata atbA*-complemented strain competing with *P. olsonii*, *P. crustosum* and *A. nidulans* was reduced to 32.2%, 51.4% and 80.4% of that of the *A. alternata atbA*-complemented strain grown alone, respectively. Taken together, the two Penicillium strains derived from tomato surfaces stimulated ATX I-III and ALP formation, whereas *A. nidulans*, which probably does not share the same habitat, did not.

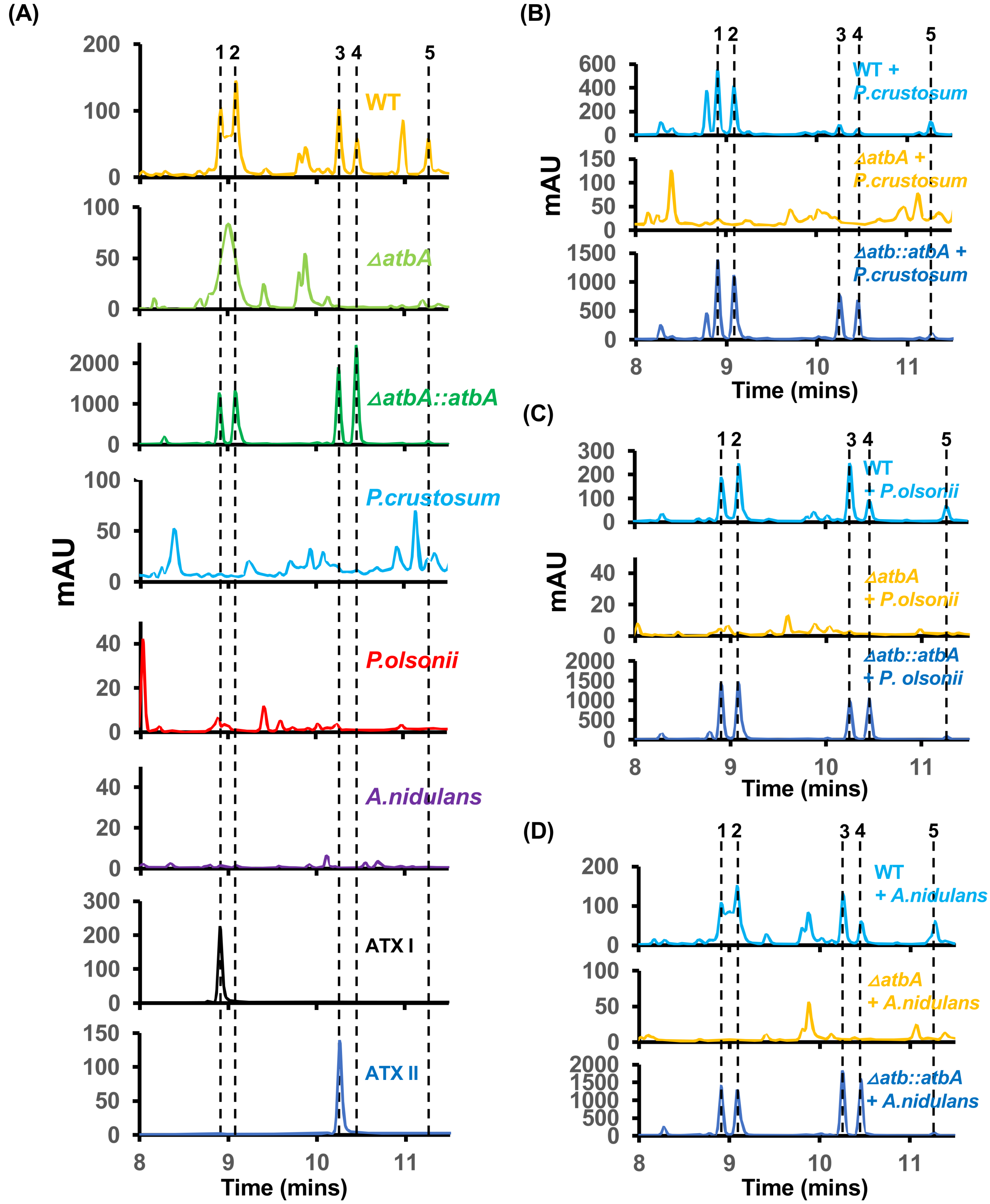

Figure S12. HPLC chromatograms at 254 nm of extracts from *A. alternata* and other fungi. (A) HPLC chromatograms at 254 nm of extracts from *A. alternata* strains (WT, *ΔatbA* and the re-complemented strain *ΔatbA::atbA*), *P. crustosum*, *P. olsonii* and *A. nidulans* on mCDB medium for 5 days at room temperature. HPLC chromatograms at 254 nm of extracts from *A. alternata* strains competing with *P. crustosum* **(B)**, *P. olsonii* **(C)** and *A. nidulans* **(D)** on mCDB medium for 5 days at room temperature. Compounds **1**-**5** are altertoxin I, alterperylenol, altertoxin II, stemphytotoxin III and altertoxin III respectively, and are indicated by dash lines.

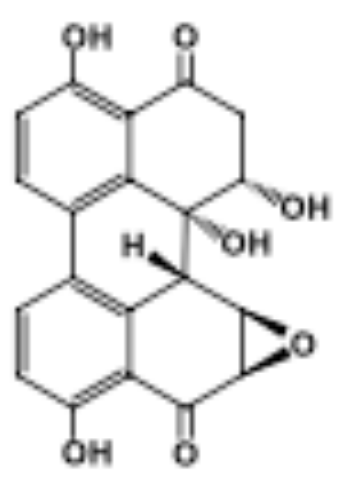
Stemphyltoxin I

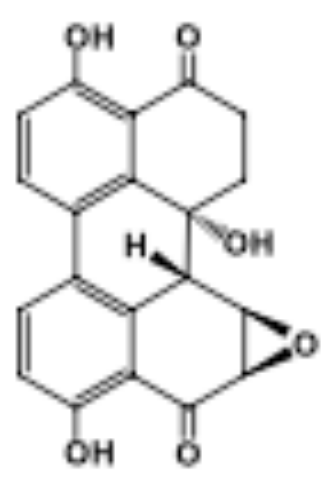
Stemphyltoxin II

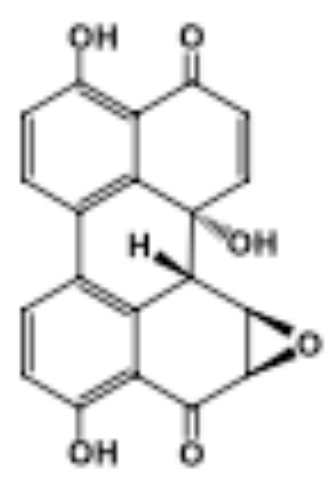
Stemphyltoxin III

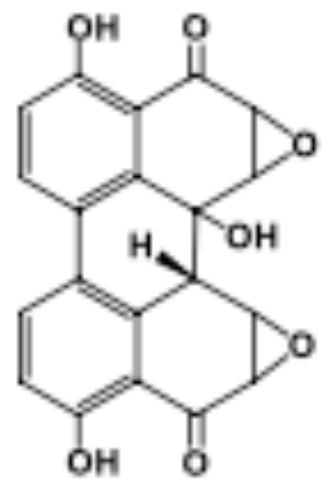
Stemphyltoxin IV

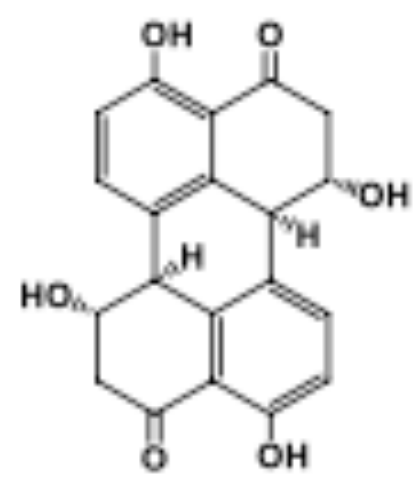
Stemphyperylenol

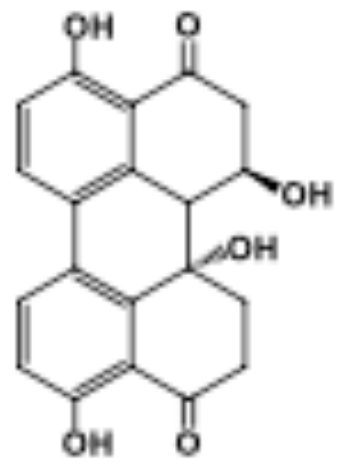
Altertoxin I

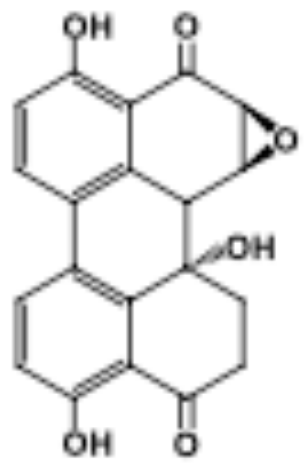
Altertoxin II

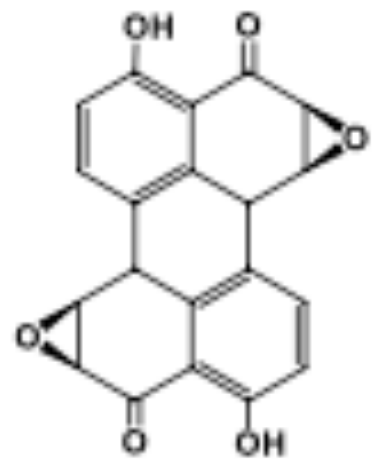
Altertoxin III

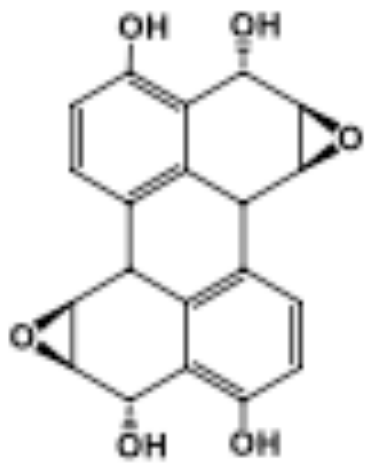
Altertoxin IV

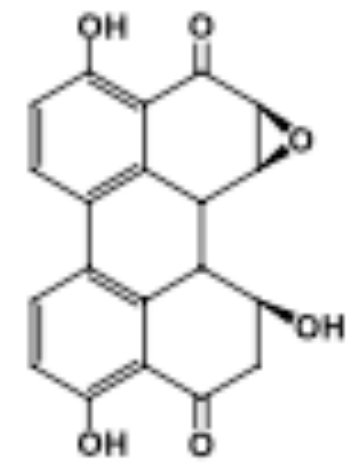
Altertoxin V

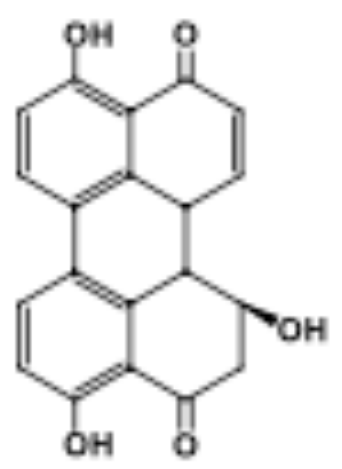
Altertoxin VI

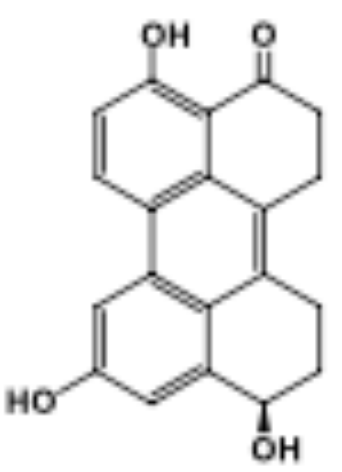
Altertoxin VII

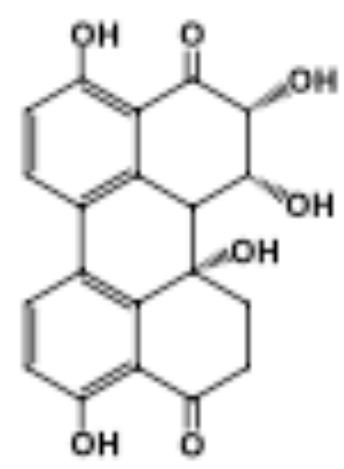
7-epi-8-hydroxyaltertoxin

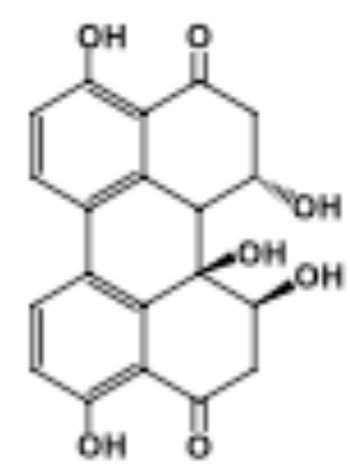
Stemphytriol

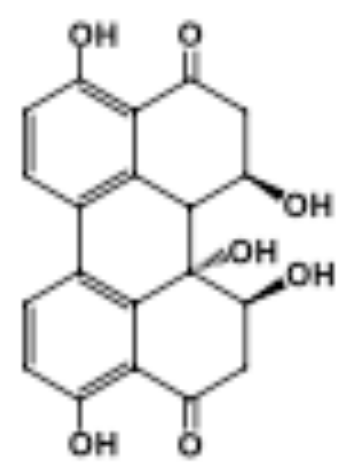
6-epi-stemphytriol

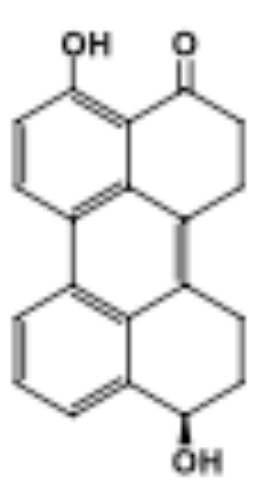
Altertoxin VIII

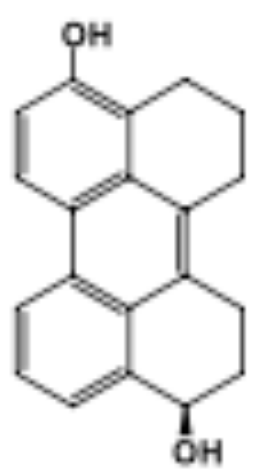
Altertoxin IX

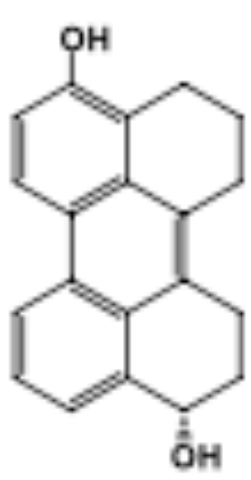
Altertoxin X

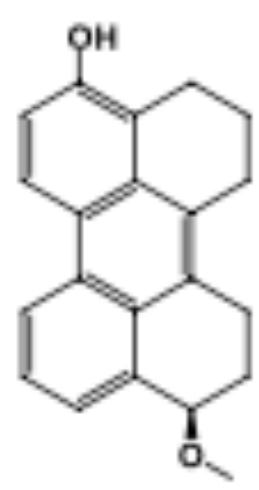
Altertoxin XI

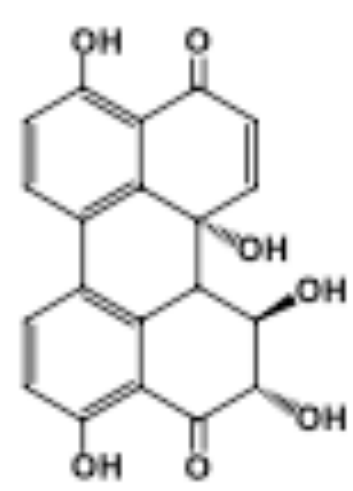
Alterlosin I

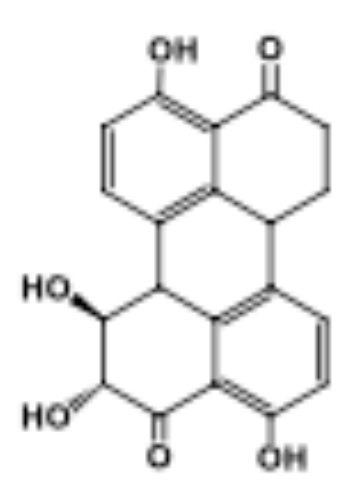
Alterlosin II

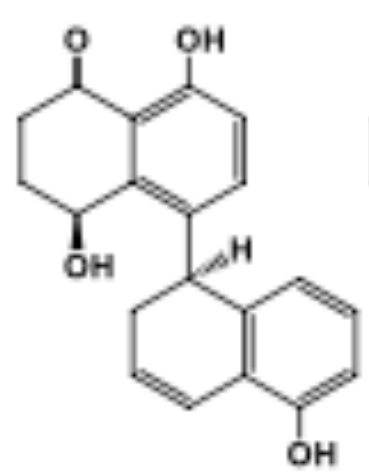
Altertoxin XII

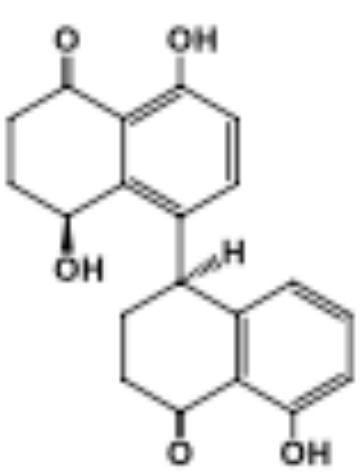
Cladosporol I

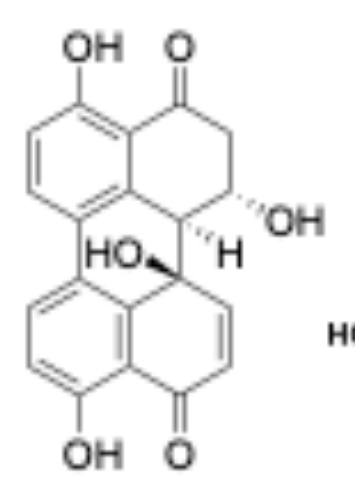
Alterperylenol

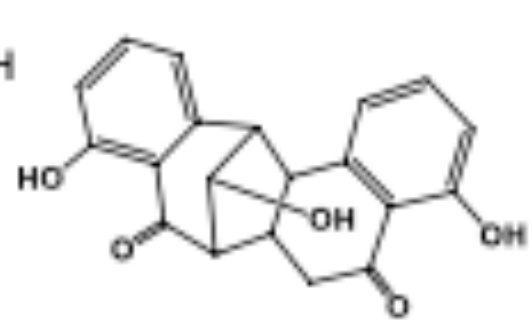
Alternatone A

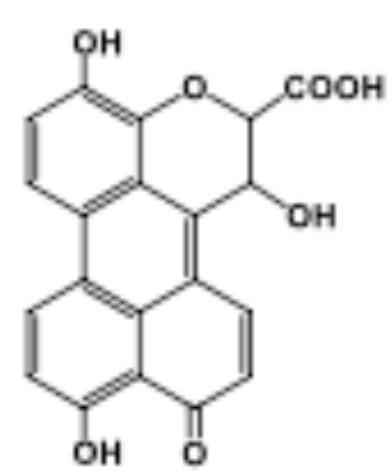
Xanalteric acid I

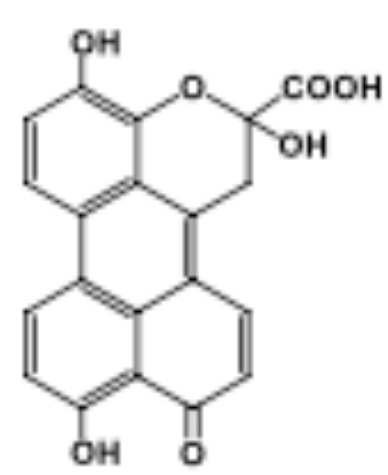
Xanalteric acid II

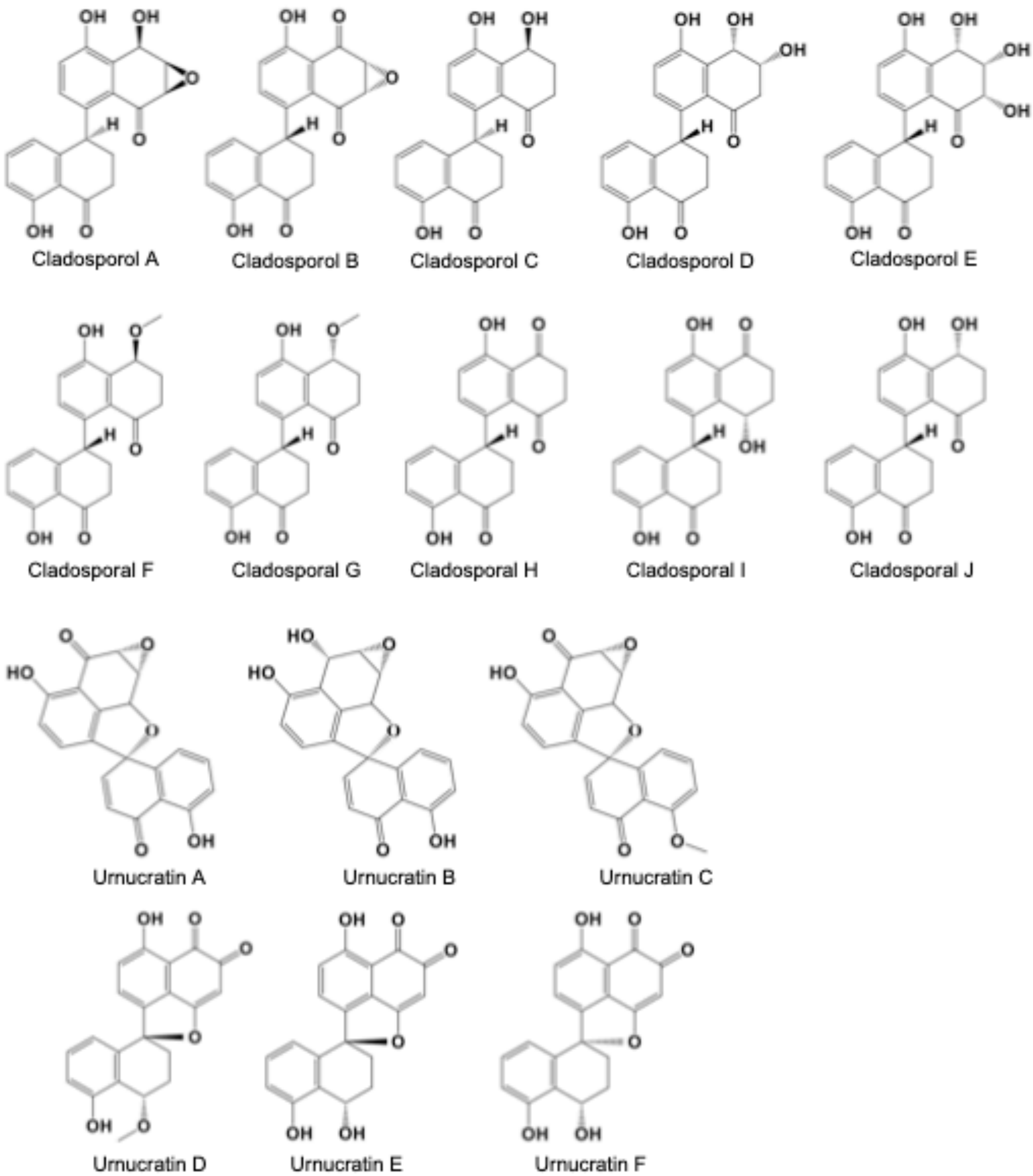

Figure S13. Chemical structures of PQs and other secondary metabolites originating from the DHN-melanin biosynthetic pathway in fungi.

Movie S1: Active site of *A. alternata* P450 AtbE using compound 7 and 11 as ligands.

Movie S2: Active site of *A. fumigatus* laccase Abr2 using one molecule of compound 7 as ligand.

Movie S3: Active site of *Berkleasmium* P450 PalA using two molecules of compound 7 as ligands.

Identification of chemical compounds

Measurement of optical rotations

The optical rotation was measured on a polarimeter Jasco DIP-370 at 20 °C using the D-line of the sodium lamp at λ = 589.3 nm. Prior to the measurement, the 12 polarimeter was calibrated with MeOH as solvent.

Experimental electronic circular dichroism (ECD) spectroscopic analysis

Experimental ECD spectra were taken on a J-1500 CD spectrometer (Jasco Deutschland GmbH, Pfungstadt, Germany). The samples were dissolved in methanol and measured in the range of 200 - 400 nm by using a 1 mm path length quartz cuvette (Hellma Analytics, Müllheim, Germany).

Physicochemical properties of the compounds described in this study

(+)-scytalone (compound **6**): Yellow oil. $[\alpha]^{25}_{D}$ +12 (*c* 0.10, MeOH); ^{1}H NMR (DMSO-d_6, 500 MHz) δ_H 12.74 (1H, s), 10.58 (1H, br s), 6.21 (1H, d, *J* = 2.3 Hz), 6.08 (1H, d, *J* = 2.3 Hz), 5.11 (1H, br s), 4.16 (1H, m), 3.00 (1H, dd, *J* = 16.0, 3.5 Hz), 2.79 (1H, ddd, *J* = 17.0, 3.7, 0.7 Hz), 2.75 (1H, dd, *J* = 16.0, 7.0 Hz), 2.56 (1H, ddd, *J* = 17.0, 7.3, 0.8 Hz). HRMS (ESI-TOF) m/z: 195.0654 $[M + H]^+$ (calcd for $C_{10}H_{11}O_4$, 195.0652). The NMR data and optical rotation correspond very well to those reported for (+)-scytalone [53].

1,8-DHN (compound **7**): White solid. ^{1}H NMR ($CDCl_3$, 500 MHz) δ_H 8.94 (2H, s), 7.32 (2H, dd, *J* = 8.4, 1.1 Hz), 7.26 (2H, dd, *J* = 8.2, 7.4 Hz), 6.78 (2H, dd, *J* = 7.5, 1.1 Hz). HRMS (ESI-TOF) m/z: 161.0600 $[M + H]^+$ (calcd for $C_{10}H_9O_2$, 161. 0597). The NMR data correspond very well to those reported for 1,8-DHN [54].

ATXIV (compound **8**): Yellow oil. $[\alpha]^{25}_{D}$ +73 (*c* 0.10, MeOH); ^{1}H NMR (DMSO-d_6, 500 MHz) δ_H 9.25 (2H, s), 7.17 (2H, d, *J* = 8.2 Hz), 6.57 (2H, d, *J* = 8.2 Hz), 5.10 (2H, br d, *J* = 5.0 Hz), 5.03 (2H, d, *J* = 5.6 Hz), 4.10 (2H, d, *J* = 3.5 Hz), 3.83 (2H, br s), 3.47 (2H, dd, *J* = 3.5, 1.6 Hz). HRMS (ESI-TOF) m/z: 351.0982 $[M - H]^-$ (calcd for $C_{20}H_{15}O_6$, 351.0874). The NMR data and optical rotation value correspond very well to those reported for ATXIV [55].

alterperylenepoxide A (compound **9**): Yellow oil. $[\alpha]^{25}_{D}$ +85 (*c* 0.10, MeOH); ^{1}H NMR (CD_3OD, 500 MHz) δ_H 8.03 (1H, br d, *J* = 9.1 Hz), 7.58 (1H, d, *J* = 8.5 Hz), 6.99 (1H, d, *J* = 9.1 Hz), 6.83 (1H, dd, *J* = 8.5, 0.8 Hz), 5.24 (1H, t, *J* = 1.5 Hz), 3.85 (1H, d, *J* = 3.9 Hz), 3.53 (1H, ddd, *J* = 3.8, 1.5, 0.8 Hz), 3.21 (1H, br d, *J* = 1.2 Hz), 3.17 (1H, ddd, *J* = 17.7, 14.2, 5.1 Hz), 2.81 (1H, ddd, *J* = 13.5, 5.1, 2.5 Hz), 2.72 (1H, ddd, *J* = 17.7, 4.2, 2.5 Hz), 2.39 (1H, ddd, *J* = 14.2, 13.5, 4.2 Hz). HRMS (ESI-TOF) m/z: 353.1033 $[M + H]^+$ (calcd for $C_{20}H_{17}O_6$, 353.1020). The NMR data and optical rotation correspond very well to those reported for alterperylenepoxide A [56].

(3*S*,4*R*,18*R*,19*R*,20*S*)-alterperylenepoxide A isomer (compound **10**): Orange solid. $[\alpha]^{25}_{D}$ +27 (*c* 0.10, MeOH); ^{1}H NMR (CD_3OD, 500 MHz) δ_H 7.99 (1H, d, *J* = 8.9 Hz), 7.65 (1H, d, *J* = 10.4 Hz), 7.63 (1H, d, *J* = 8.4 Hz), 6.99 (1H, d, *J* = 8.9 Hz), 6.82 (1H, dd, *J* = 8.9, 0.8 Hz), 6.48 (1H, d, *J* = 10.4 Hz), 5.15 (1H, t, *J* = 1.5 Hz), 3.96 (1H, d, *J* = 4.0 Hz), 3.56 (1H, ddd, *J* = 4.0, 1.4, 0.6 Hz), 3.32 (1H, overlapped). HRMS (ESI-TOF) m/z: 351.0986 $[M + H]^+$ (calcd for $C_{20}H_{15}O_6$, 351.0863). The NMR data and

optical rotation correspond very well to those reported for (3*S*,4*R*,18*R*,19*R*,20*S*)-alterperylenepoxide A isomer [57].

alterperylenol (compound **2**): Yellow powder. $[\alpha]_D^{25}$ +300 (*c* 0.10, MeOH); ^{1}H NMR (DMSO-d_6, 500 MHz) δ_H 12.49 (1H, s), 12.41 (1H, s), 8.11 (1H, d, *J* = 8.9 Hz), 8.05 (1H, d, *J* = 8.9 Hz), 7.92 (1H, d, *J* = 10.4 Hz), 7.09 (1H, d, *J* = 8.8 Hz), 6.99 (1H, dd, *J* = 8.8, 0.9 Hz), 6.38 (1H, d, *J* = 10.4 Hz), 5.77 (1H, d, *J* = 1.4 Hz), 5.73 (1H, d, *J* = 6.2 Hz), 4.53 (1H, m), 3.14 (1H, d, *J* = 9.5 Hz) , 3.00 (1H, dd, *J* = 15.9, 11.9 Hz) , 2,93 (1H, dd, *J* = 15.9, 5.1 Hz). HRMS (ESI-TOF) m/z: 351.0849 $[M + H]^+$ (calcd for $C_{20}H_{15}O_6$, 351.0863). The NMR data and optical rotation correspond very well to those reported for alterperylenol [58].

STX III (compound **4**): Yellow powder. $[\alpha]_D^{25}$ +330 (*c* 0.10, MeOH); ^{1}H NMR ($(CD_3)_2CO$, 500 MHz) δ_H 12.46 (1H, s), 12.22 (1H, br s), 8.14 (2H, dd, *J* = 8.8, 1.7 Hz), 7.71 (1H, d, *J* = 10.4 Hz), 7.20 (1H, d, *J* = 8.8 Hz), 7.13 (1H, dd, *J* = 8.8, 0.9 Hz), 6.64 (1H, d, *J* = 10.4 Hz), 4.49 (1H, d, *J* = 3.6 Hz), 4.01 (1H, s), 3.90 (1H, s), 3.83 (1H, dd, *J* = 3.6, 0.9 Hz). HRMS (ESI-TOF) m/z: 349.0695 $[M + H]^+$ (calcd for $C_{20}H_{13}O_6$, 349.0707). The NMR data and optical rotation correspond very well to those reported for STX III [59].
ATX III (compound **5**): Yellow powder. $[\alpha]_D^{25}$ +340 (*c* 0.10, MeOH); ^{1}H NMR ($CDCl_3$, 500 MHz) δ_H 11.50 (2H, s), 7.60 (2H, d, *J* = 8.6 Hz), 6.90 (2H, d, *J* = 8.6 Hz), 4.60 (2H, d, *J* = 3.4 Hz), 4.22 (2H, s), 3.86 (2H, dd, *J* = 3.4, 0.4 Hz). HRMS (ESI-TOF) m/z: 349.0688 $[M + H]^+$ (calcd for $C_{20}H_{13}O_6$, 349.0707). The NMR data and optical rotation correspond very well to those reported for ATX III [60].

ATX II (compound **3**): Yellow powder. $[\alpha]_D^{25}$ +237 (*c* 0.10, MeOH); ^{1}H NMR ($CDCl_3$, 500 MHz) δ_H 12.73 (1H, s), 12.14 (1H, s), 7.91 (1H, d, *J* = 8.9 Hz), 7.86 (1H, d, *J* = 8.9 Hz), 7.12 (1H, d, *J* = 8.9 Hz), 7.07 (1H, dd, *J* = 8.9, 0.6 Hz), 4.23 (1H, d, *J* = 3.6 Hz), 3.72 (1H, dd, *J* = 3.6, 0.5 Hz), 3,55 (1H, s), 3.26 (1H, ddd, *J* = 17.5, 14.2, 4.9 Hz), 2.89 (1H, ddd, *J* = 13,6, 4.9, 2.5 Hz), 2.83 (1H, ddd, *J* = 17.5, 3.9, 2.5 Hz) , 2.41 (1H, dd, *J* = 13.6, 3.9 Hz). HRMS (ESI-TOF) m/z: 351.0838 $[M + H]^+$ (calcd for $C_{20}H_{15}O_6$, 351.0863). The NMR data and optical rotation correspond very well to those reported for ATX II [60].

2-2'-dihydroxynaphthalene dimer (Figure 6D)
White solid; ^{1}H NMR (methanol-d_4, 500 MHz) δ_H 7.37 (4H, m), 7.30 (2H, dd, *J* = 8.2, 0.9 Hz), 7.25 (2H, dd, *J* = 8.2, 7.6 Hz), 6.75 (2H, dd, *J* = 7.6, 0.9 Hz); ^{13}C NMR (methanol-d_4, 125 MHz) δ_C 155.5, 152.0, 137.8, 131.1, 127.7, 121.3, 120.6, 120.5, 116.5, 109.8; HRMS (ESI-TOF) m/z: 319.0973 $[M + H]^+$ (calcd for $C_{20}H_{15}O_4$, 319.0965). These data correspond well to those of the 2-2'-dihydroxynaphthalene dimer [61].

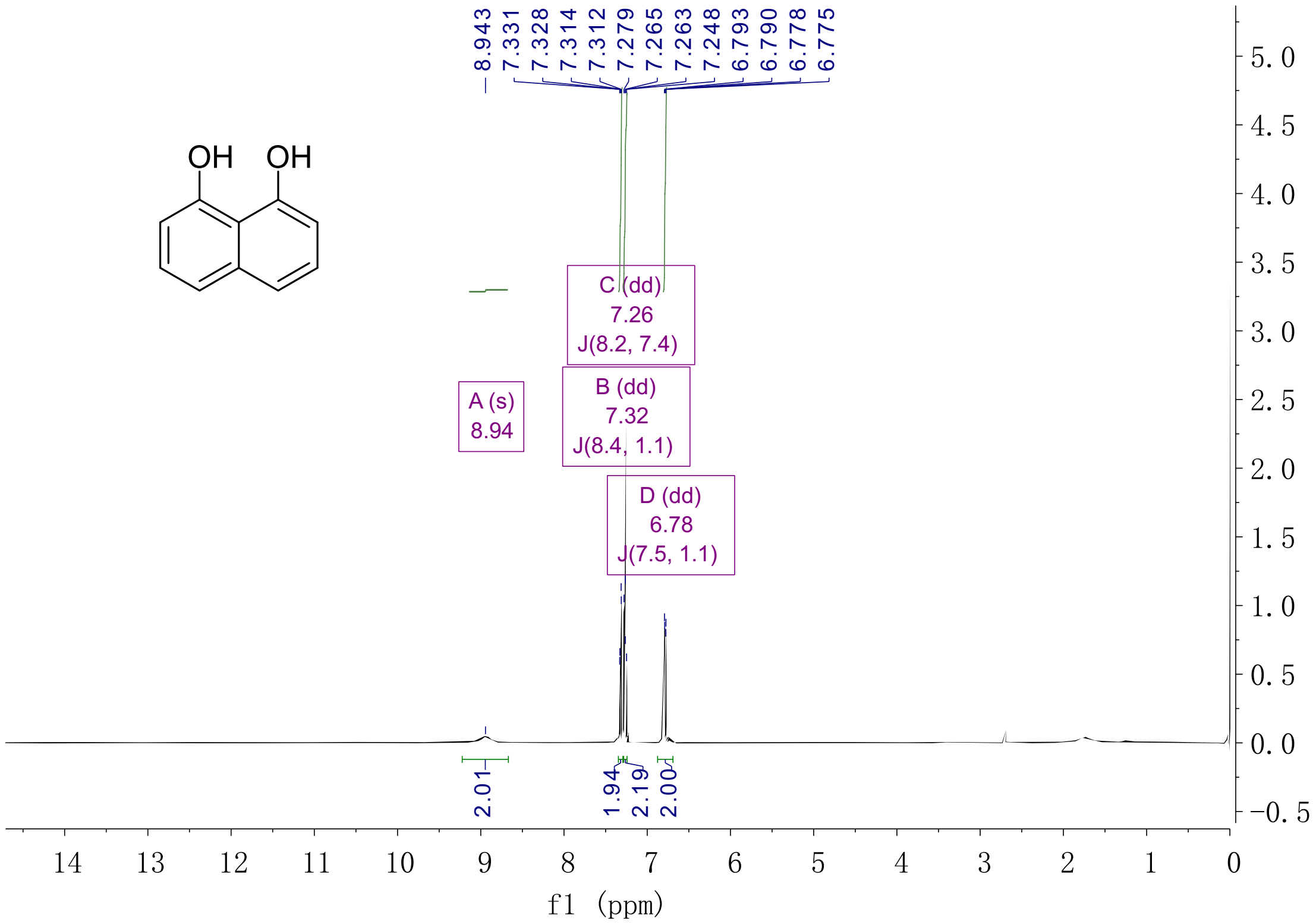

^{1}H NMR spectrum of compound **7** in $CDCl_3$ (500 MHz)

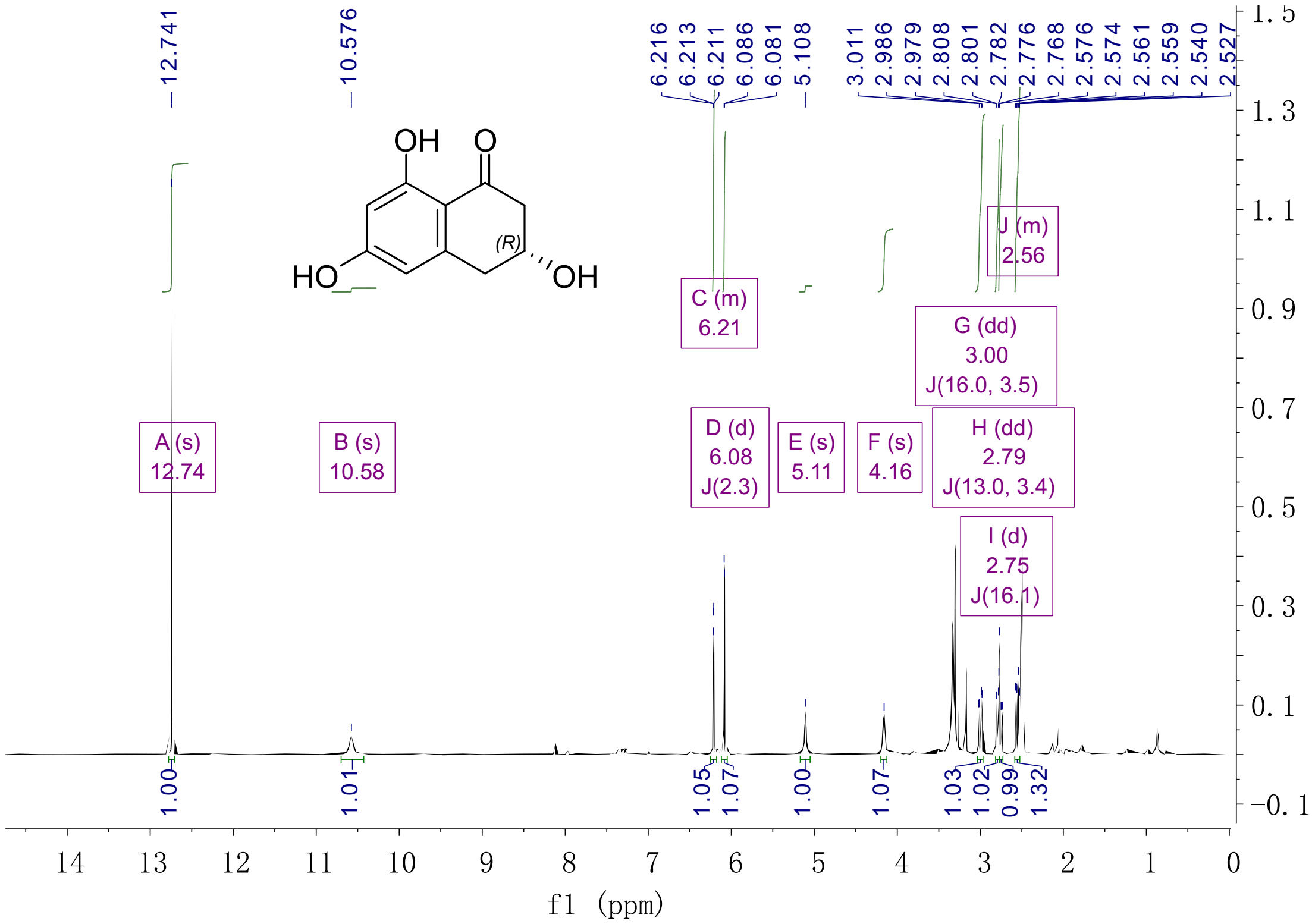

^{1}H NMR spectrum of compound **6** in DMSO-d_6 (500 MHz).

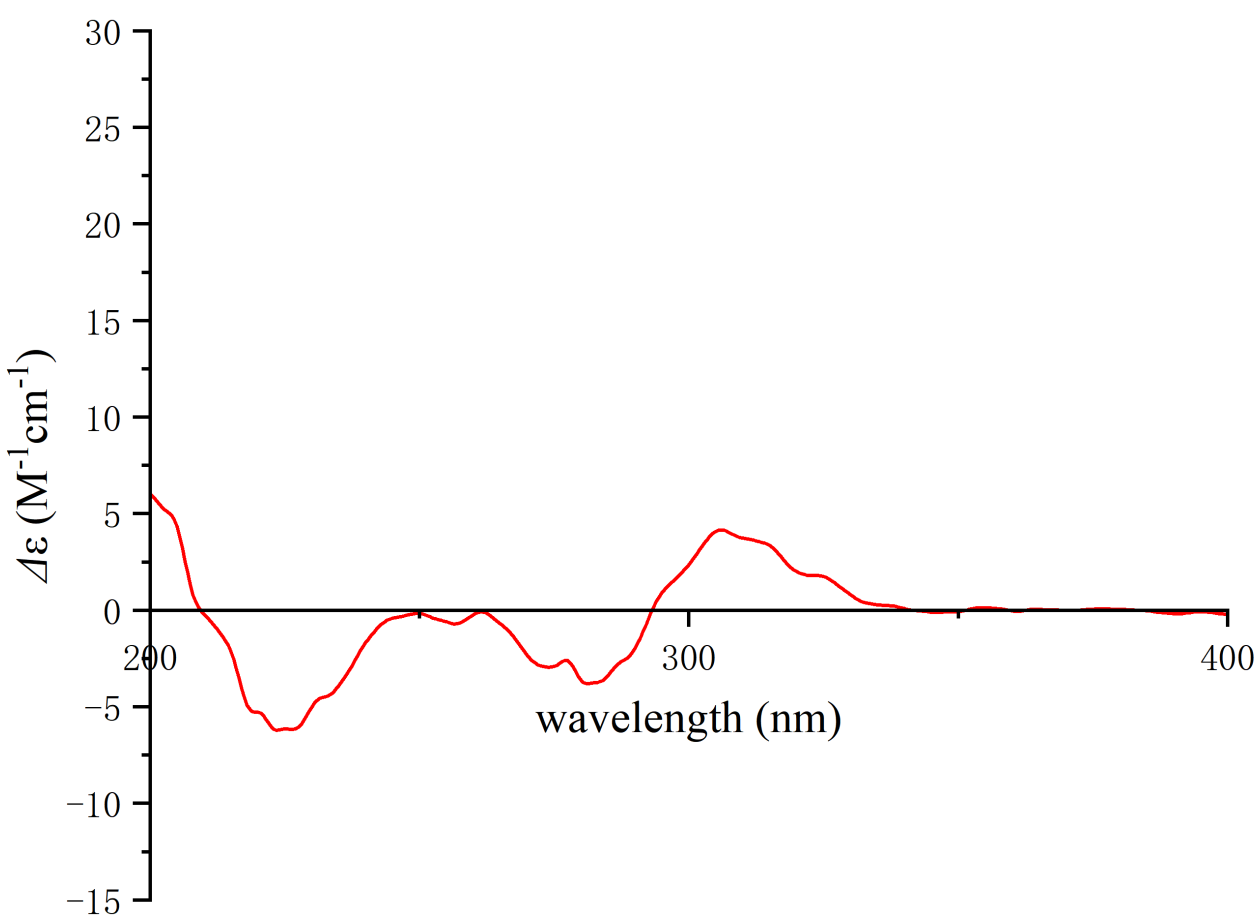

Experimental ECD spectrum of compound **6** in MeOH.

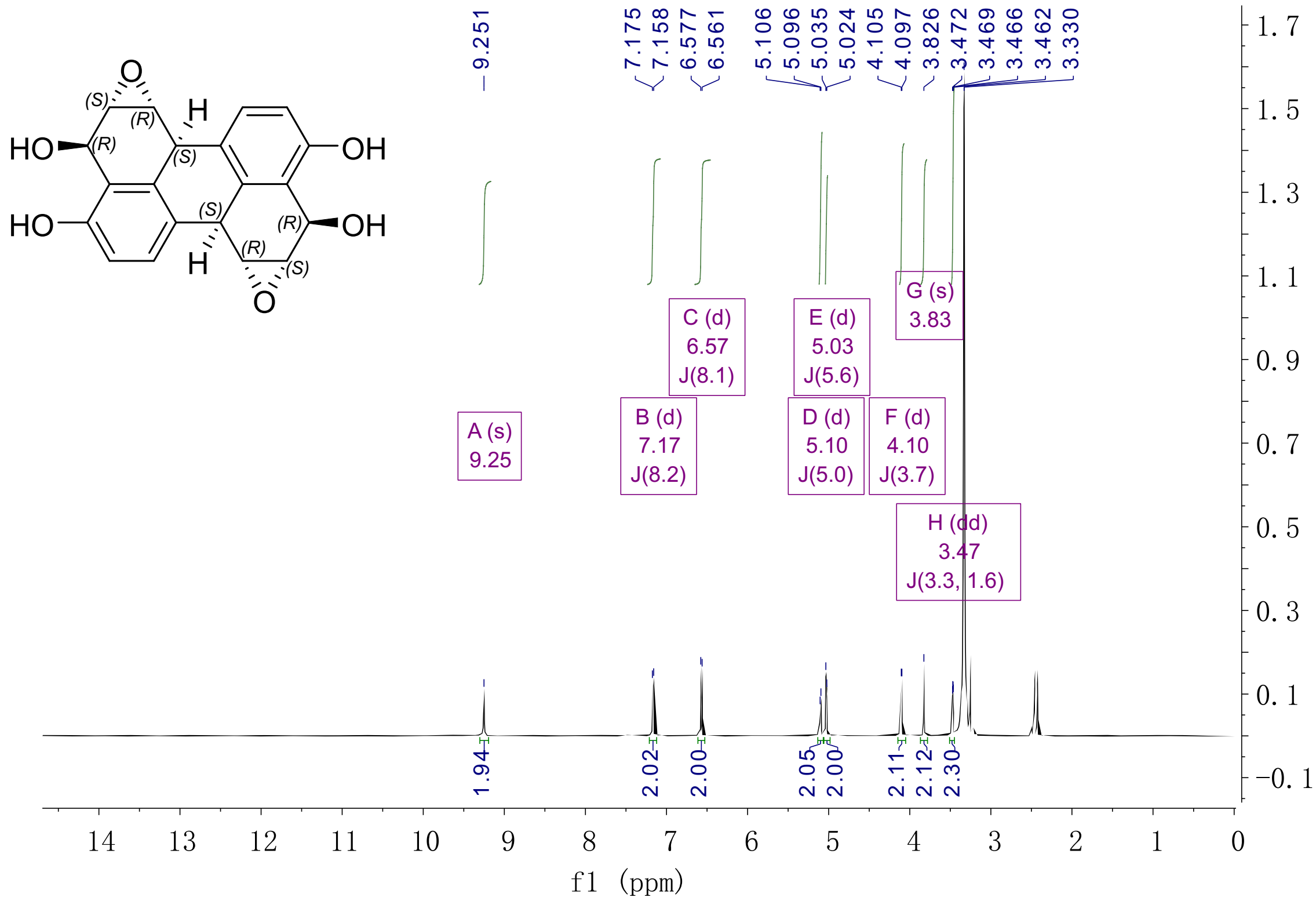

^{1}H NMR spectrum of compound **8** in DMSO-d_6 (500 MHz).

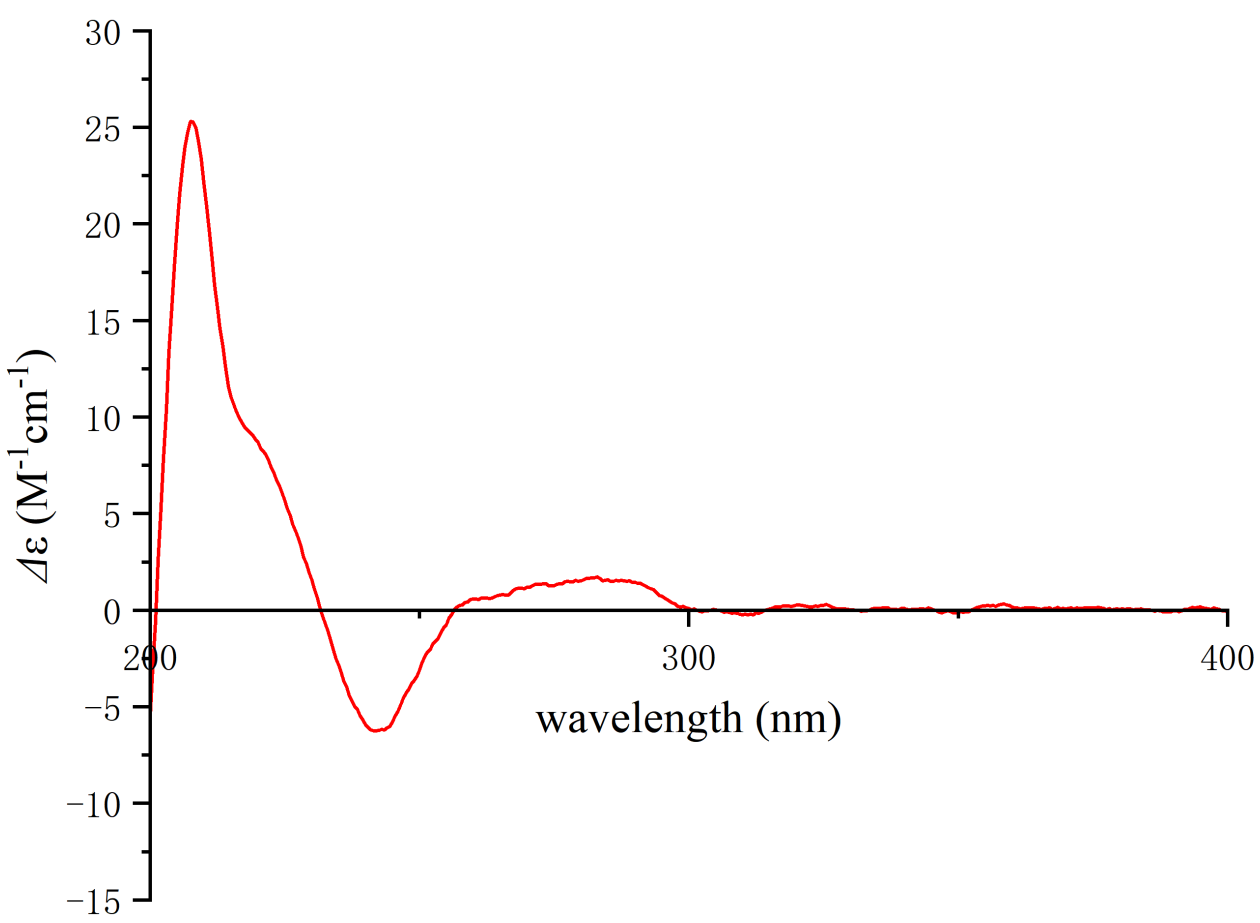

Experimental ECD spectrum of compound **8** in MeOH.

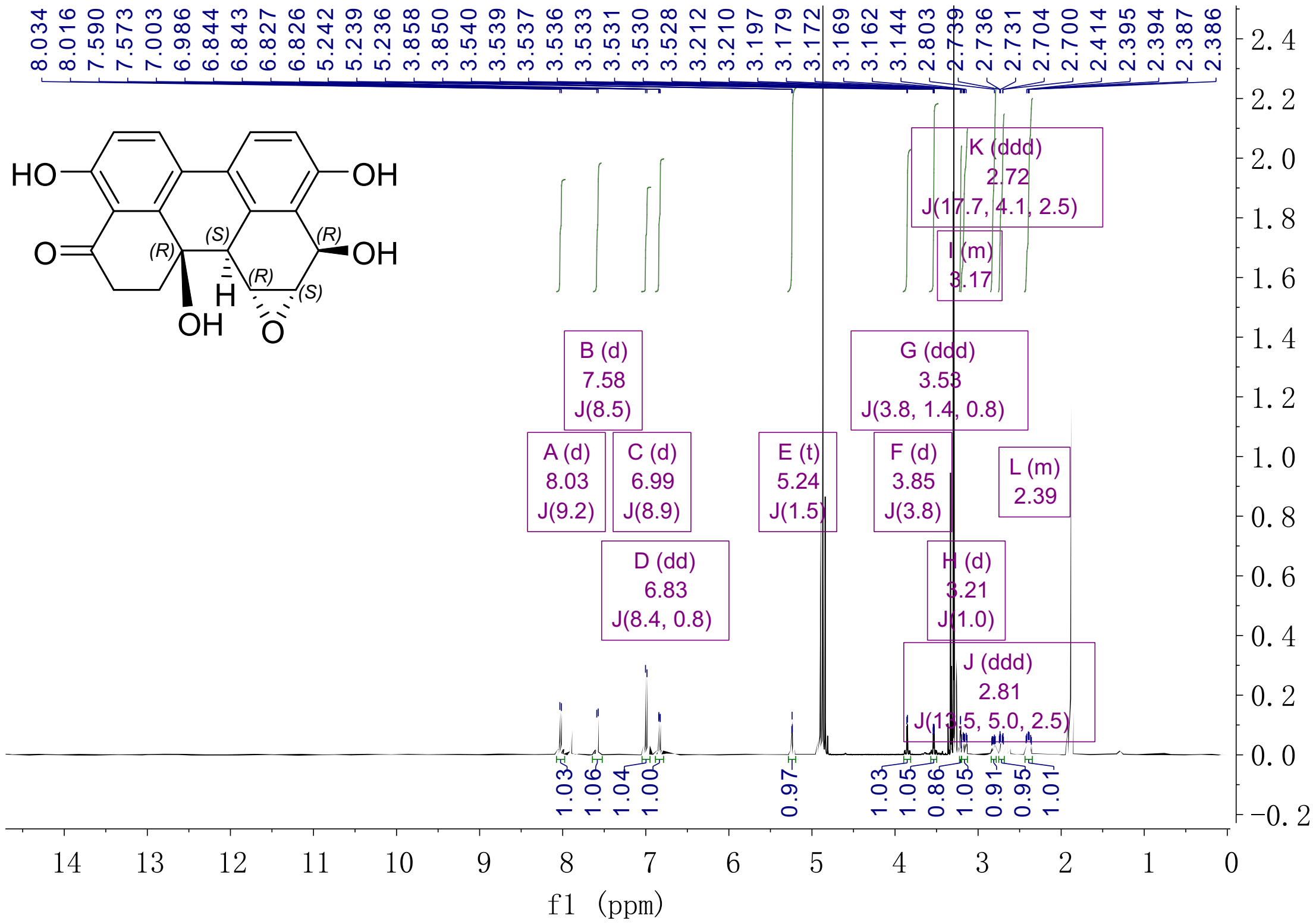

^{1}H NMR spectrum of compound **9** in CD_3OD (500 MHz).

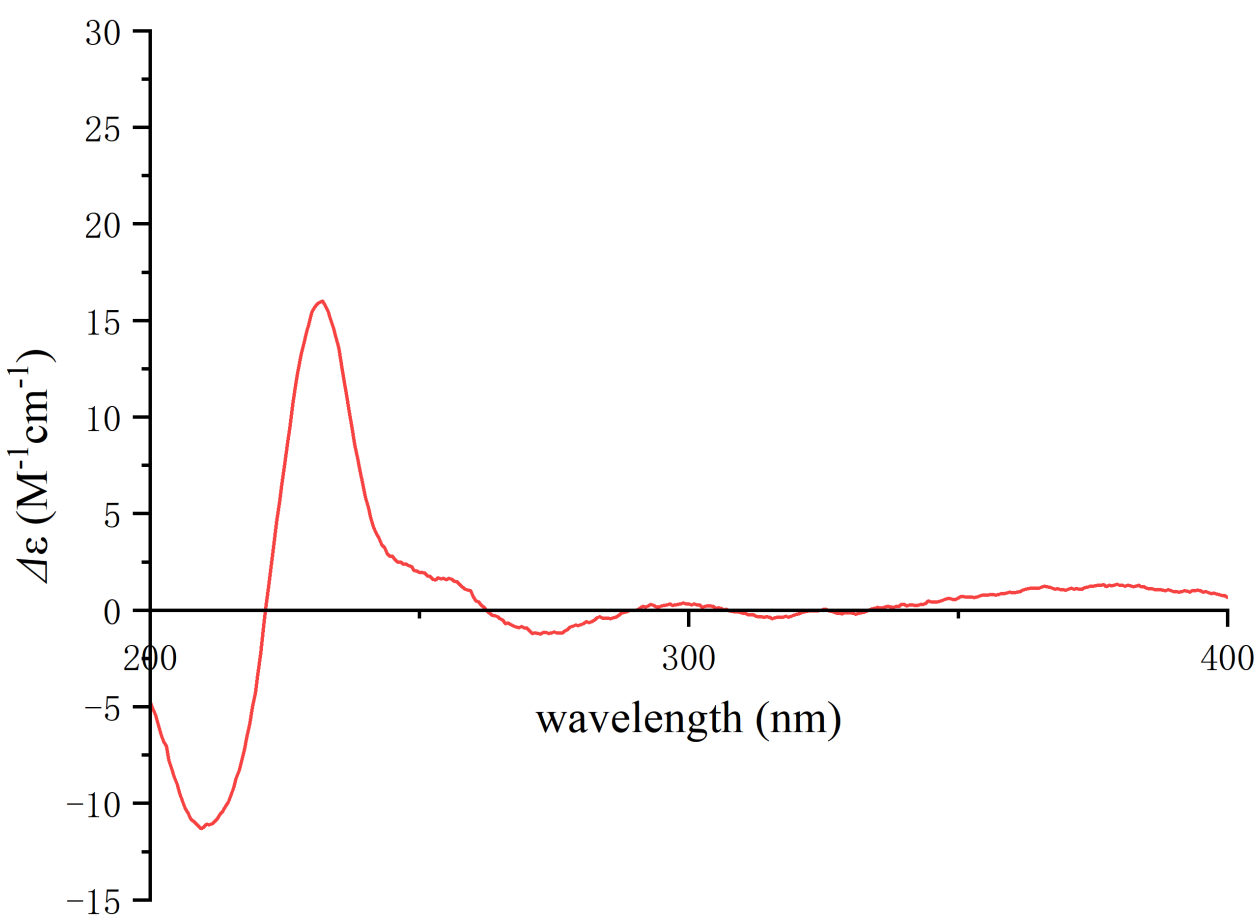

Experimental ECD spectrum of compound **9** in MeOH.

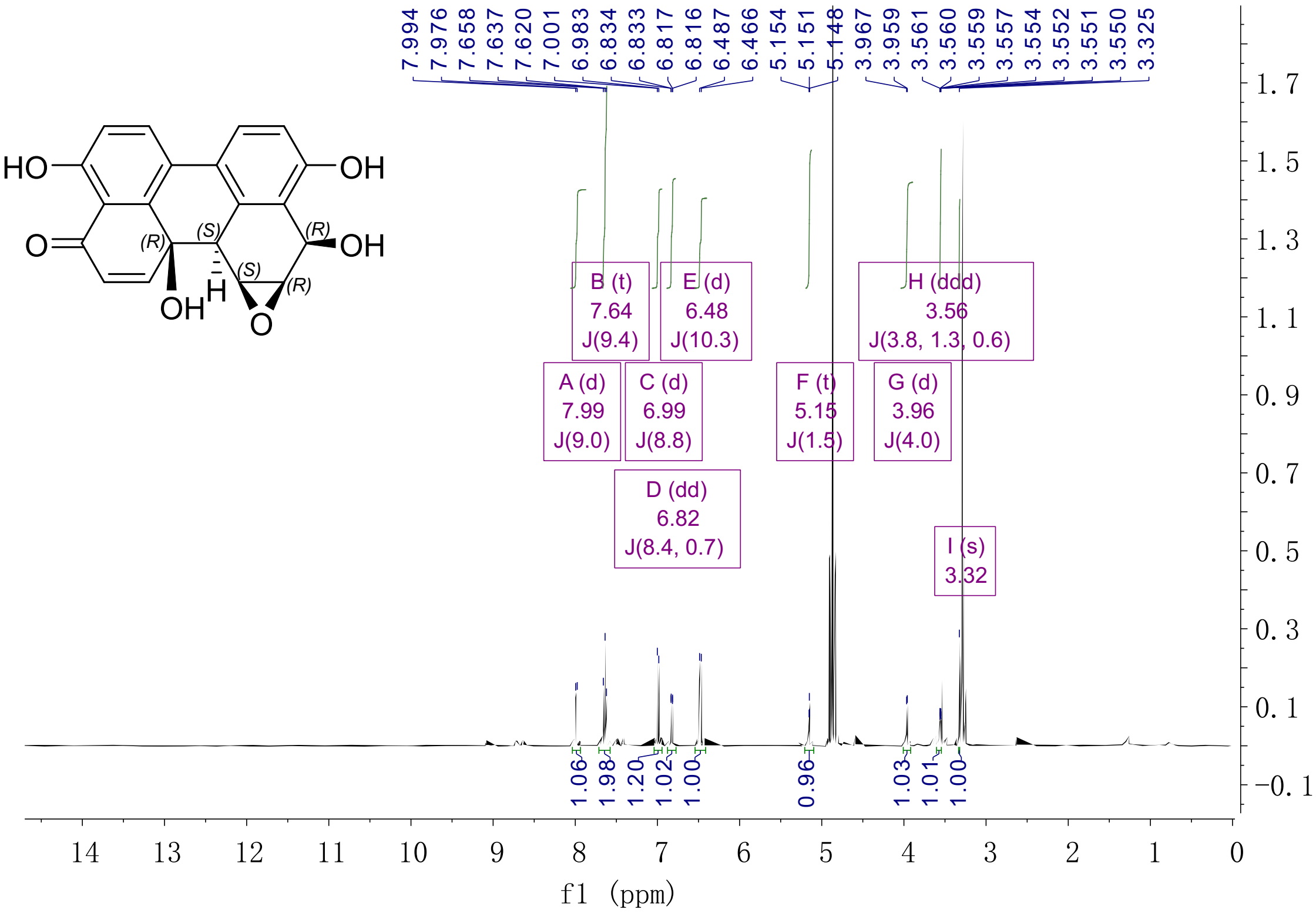

^{1}H NMR spectrum of compound **10** in CD_3OD (500 MHz)

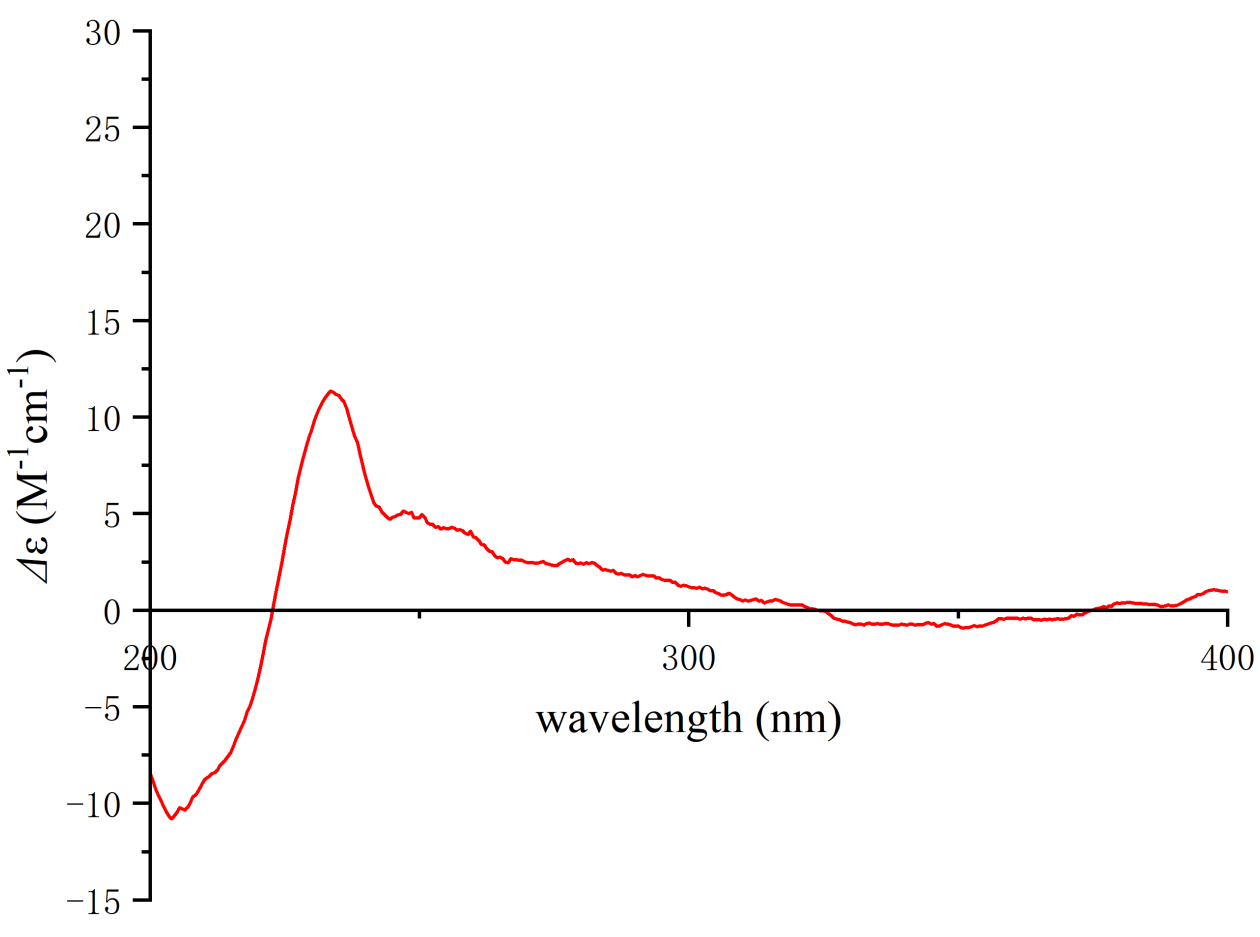

Experimental ECD spectrum of compound **10** in MeOH.

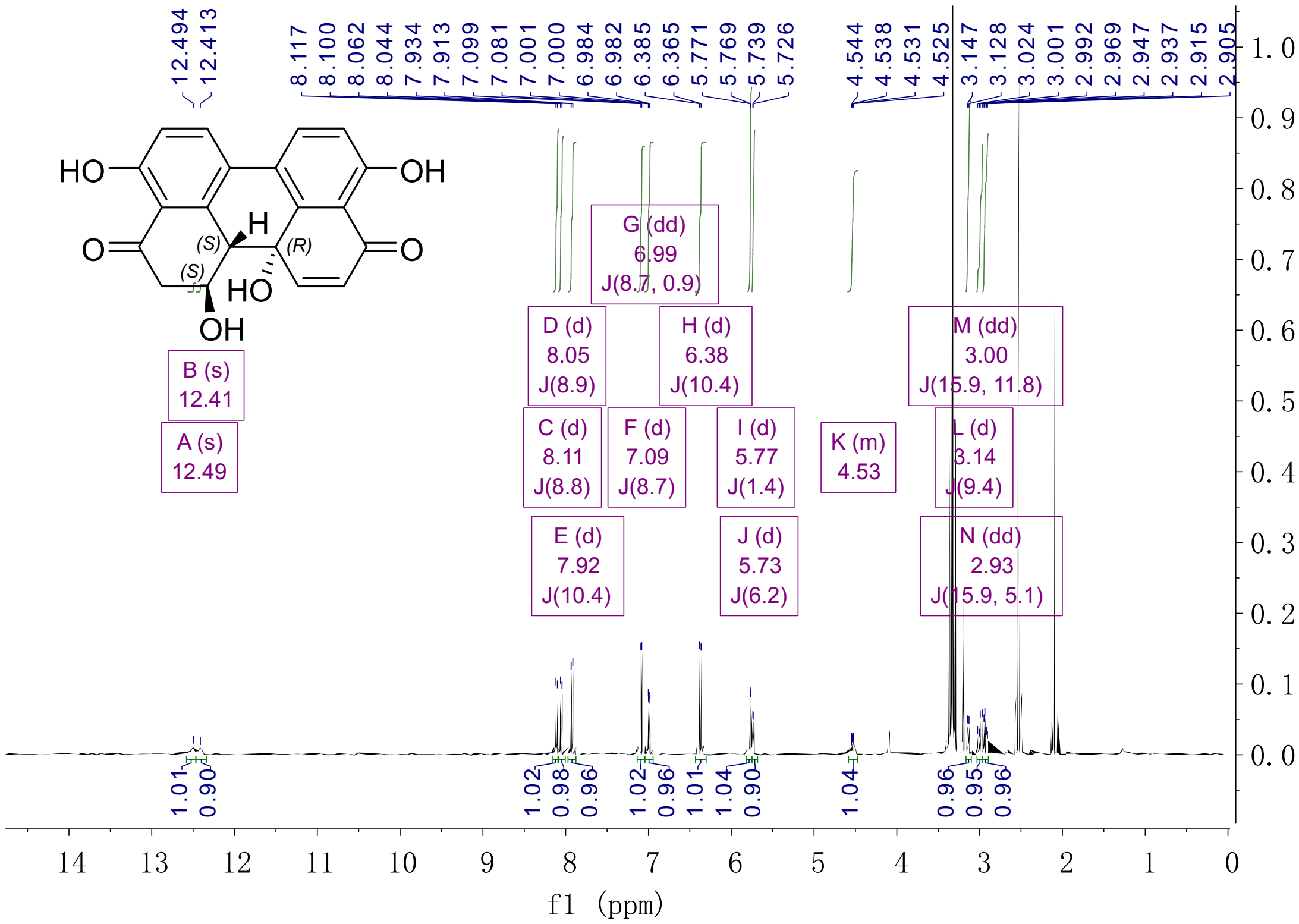

^{1}H NMR spectrum of compound **2** in DMSO-d_6 (500 MHz).

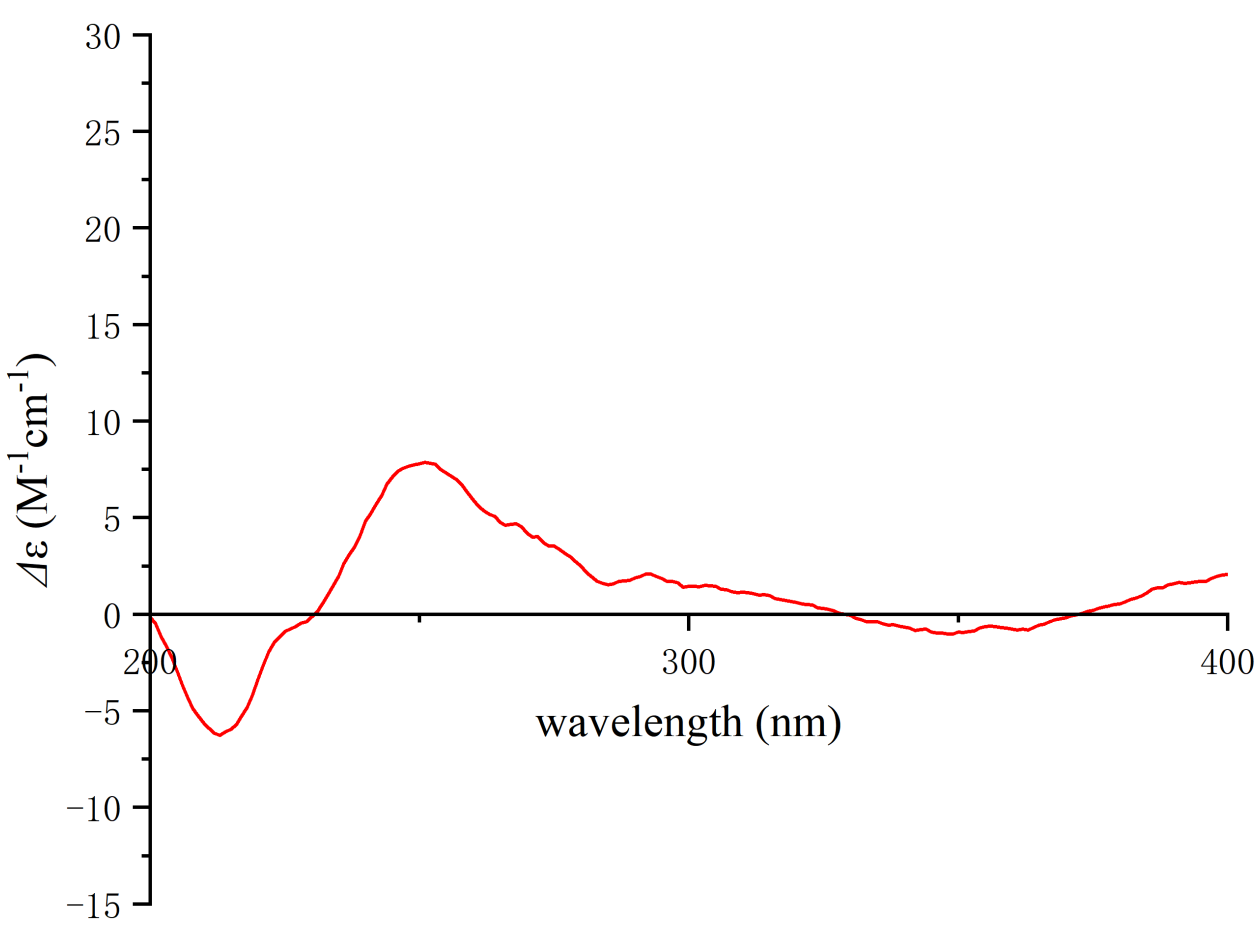

Experimental ECD spectrum of compound **2** in MeOH.

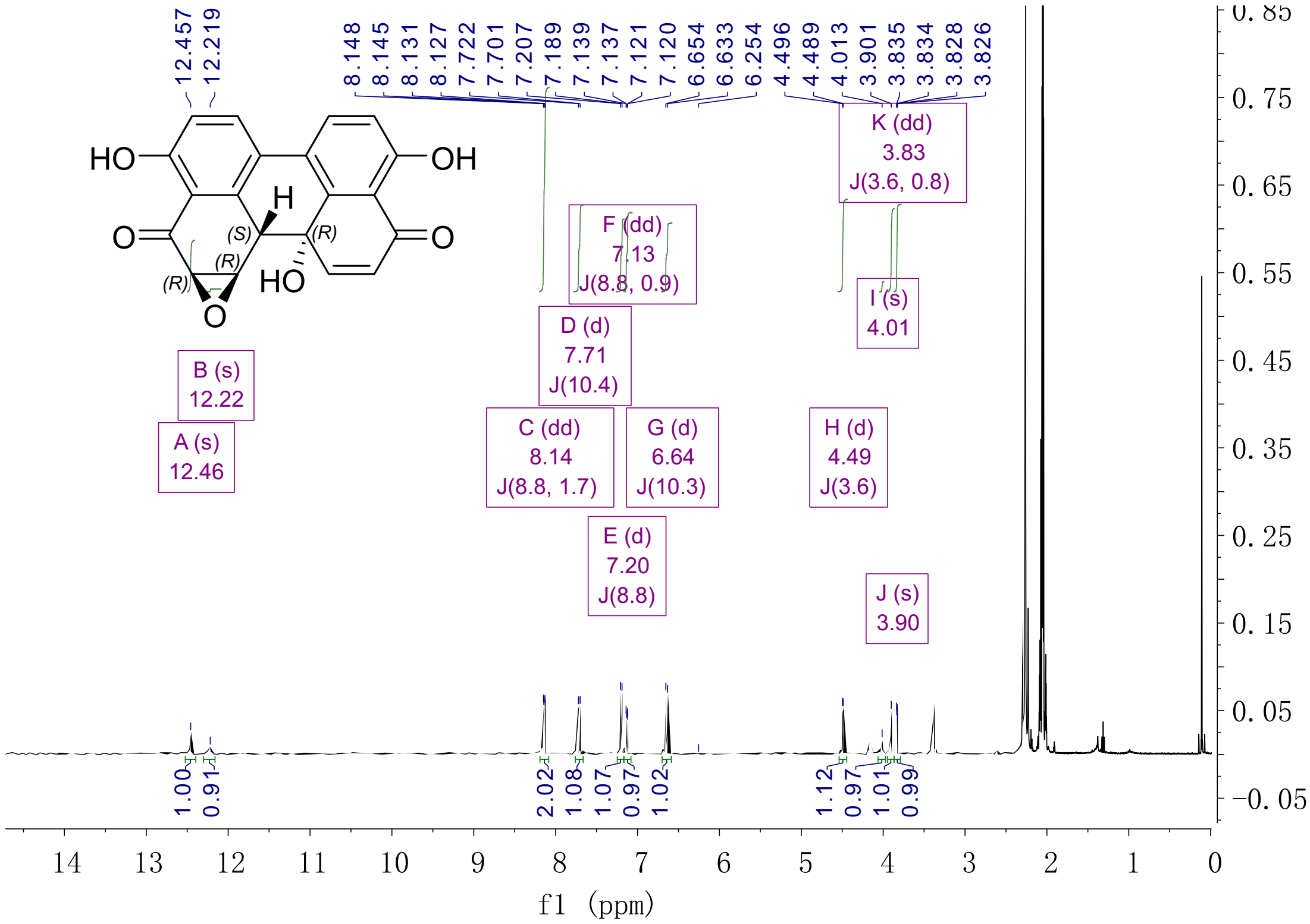

^{1}H NMR spectrum of compound **4** in $(CD_3)_2CO$ (500 MHz).

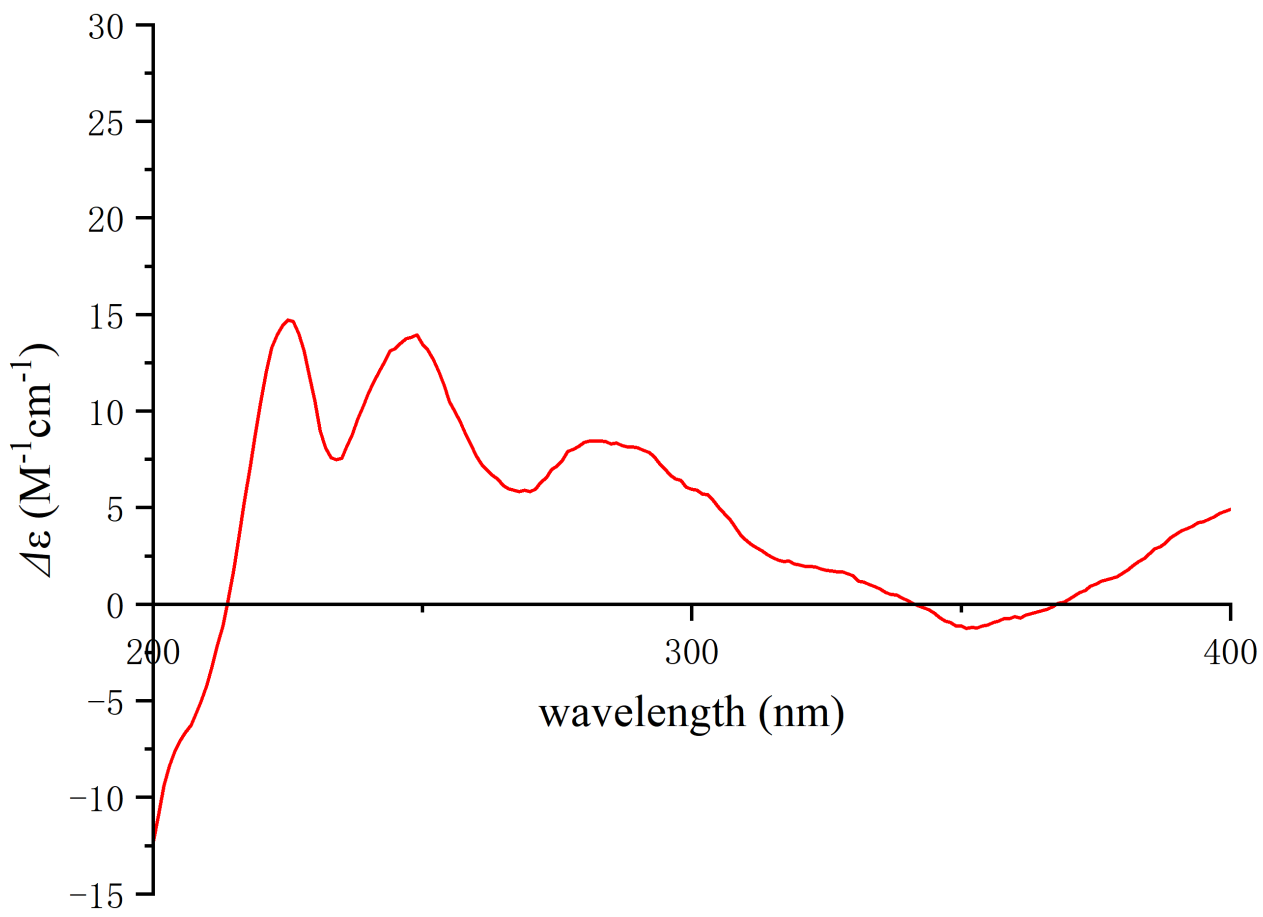

Experimental ECD spectrum of compound **4** in MeOH.

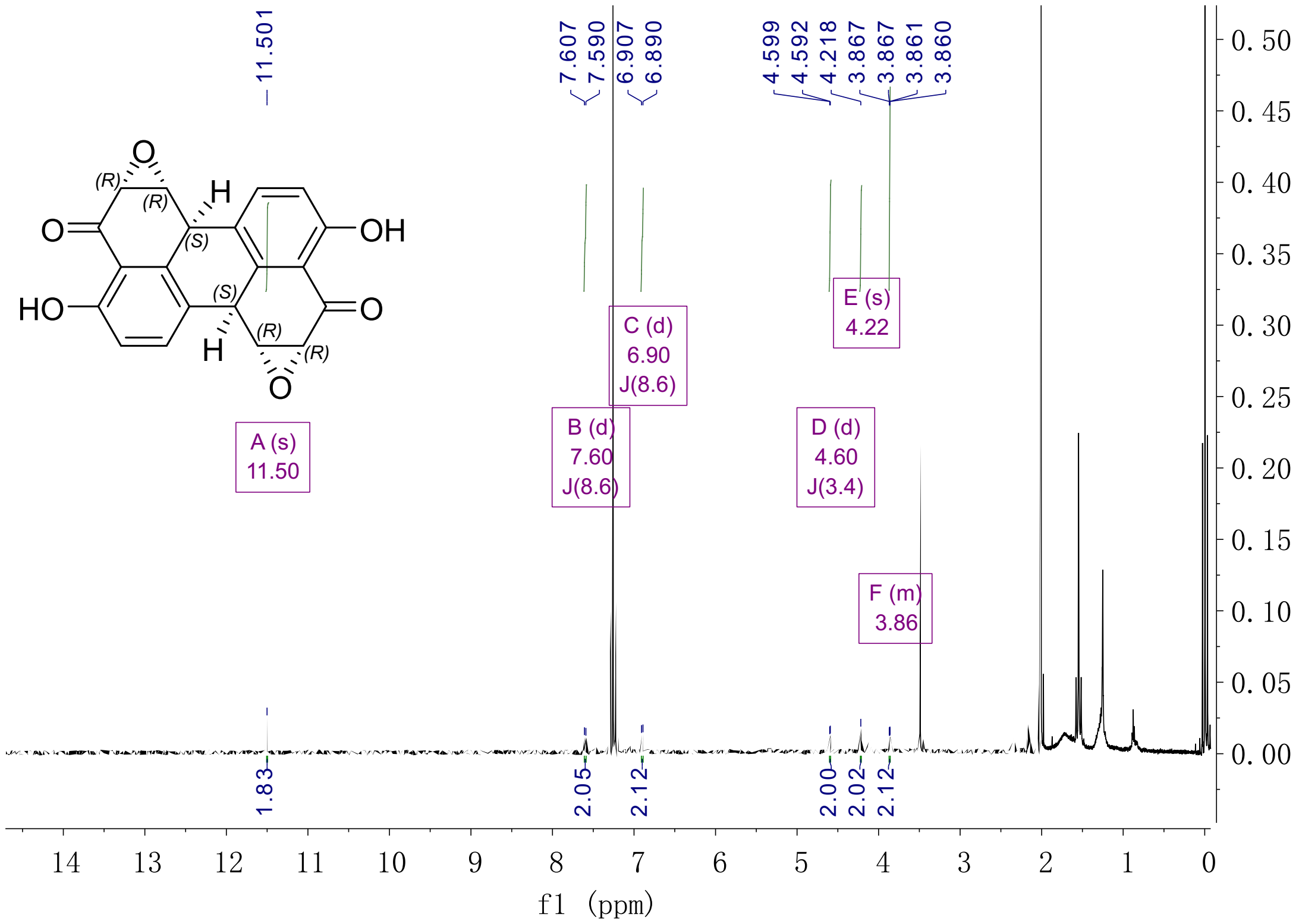

^{1}H NMR spectrum of compound **5** in $CDCl_3$ (500 MHz).

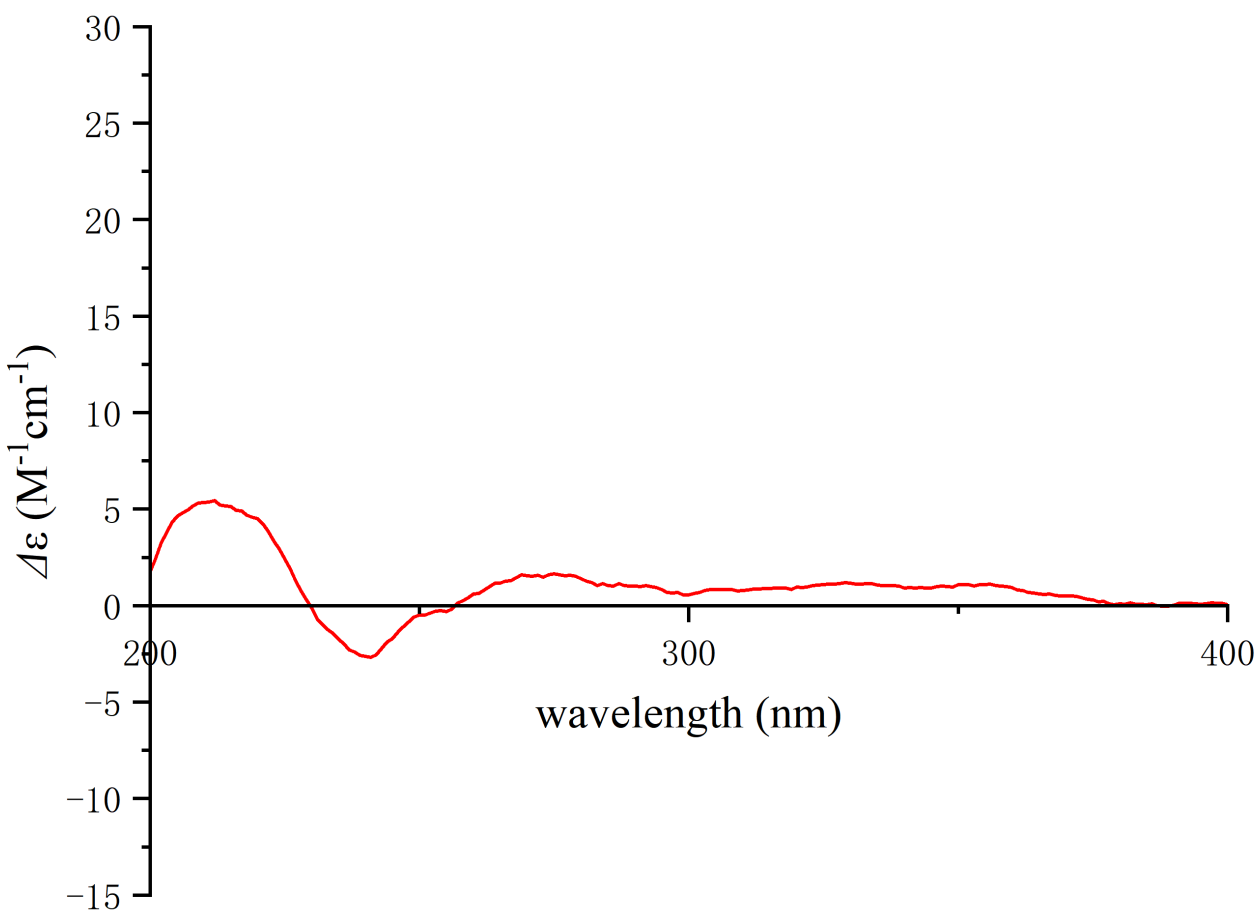

Experimental ECD spectrum of compound **5** in MeOH.

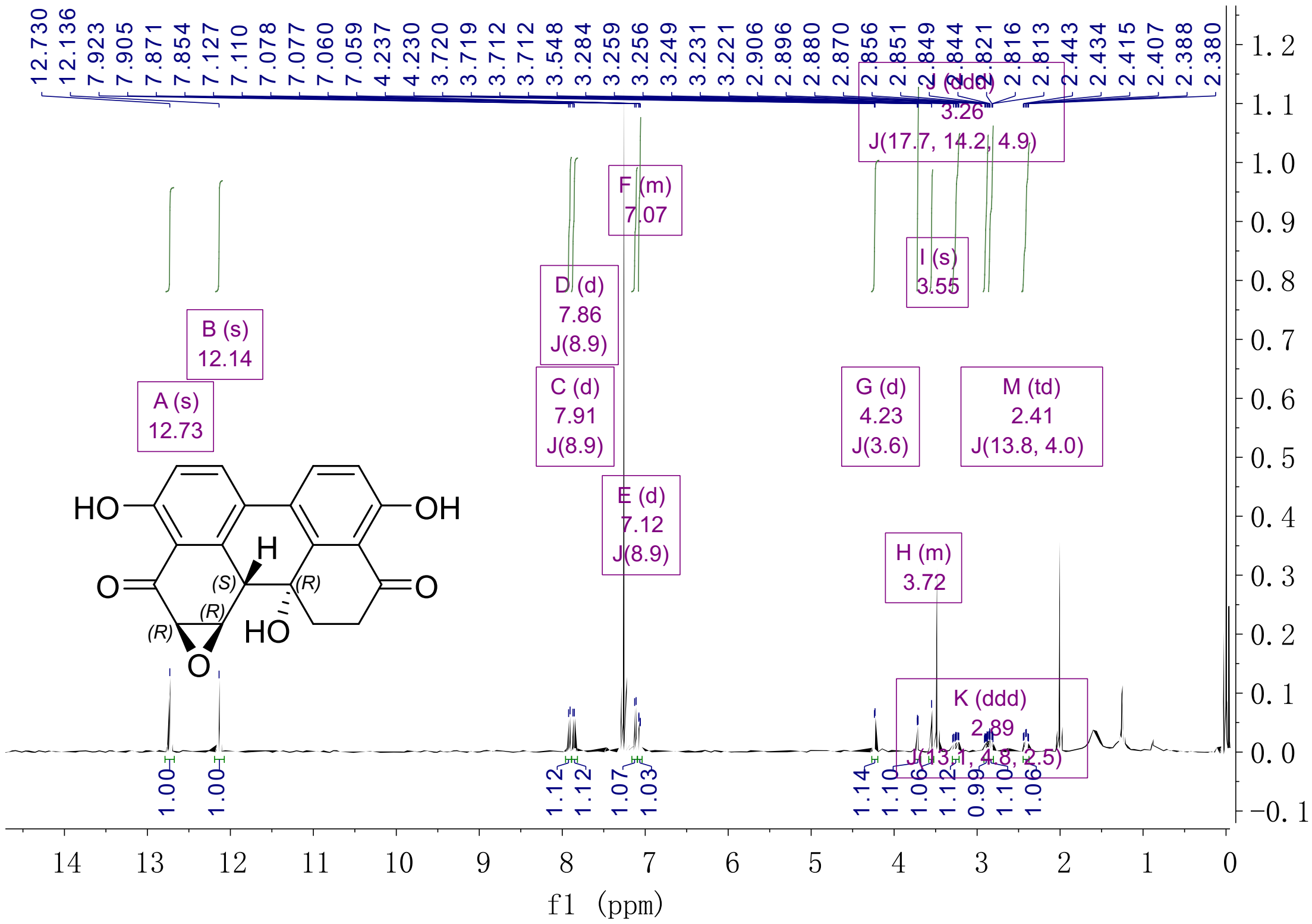

^{1}H NMR spectrum of compound **3** in $CDCl_3$ (500 MHz).

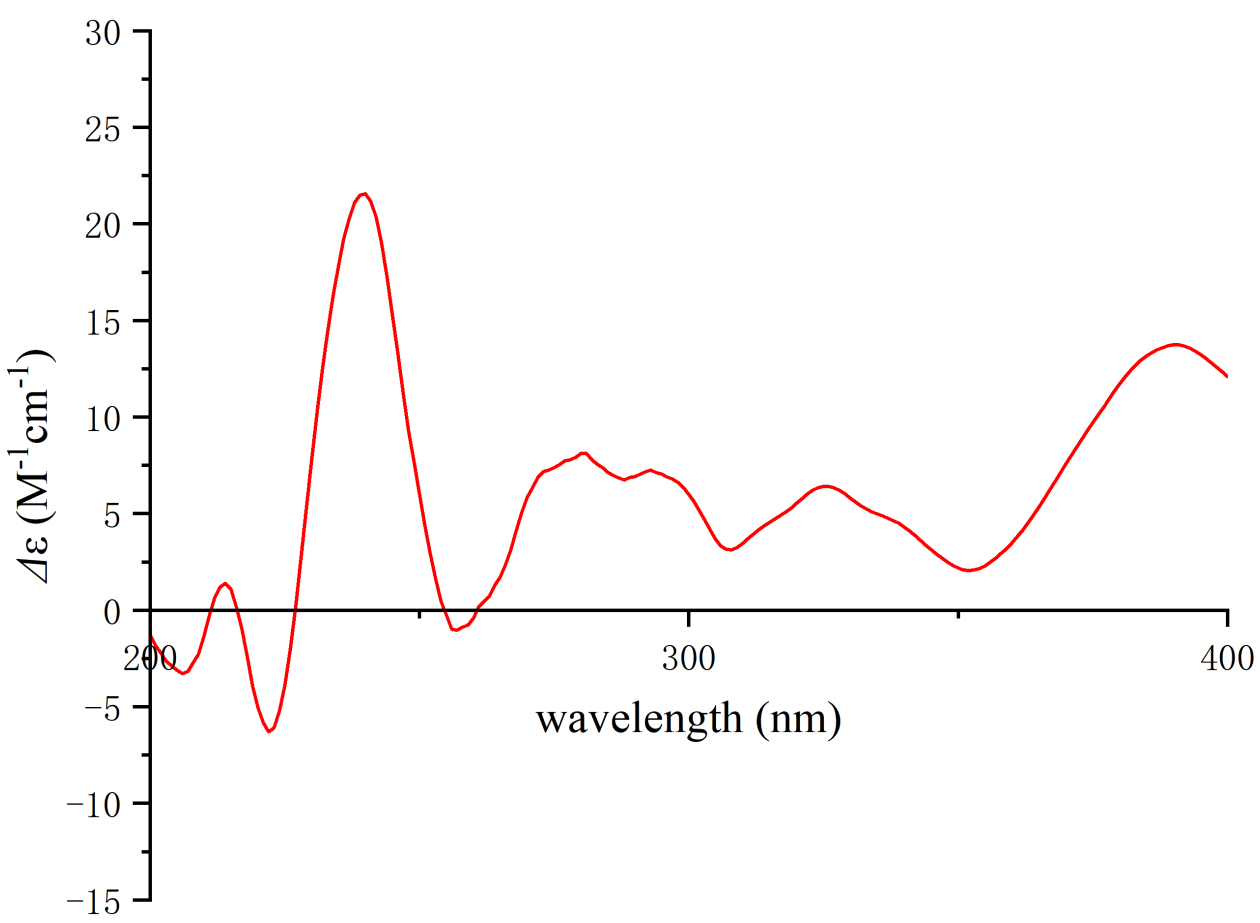

Experimental ECD spectrum of compound **3** in MeOH.

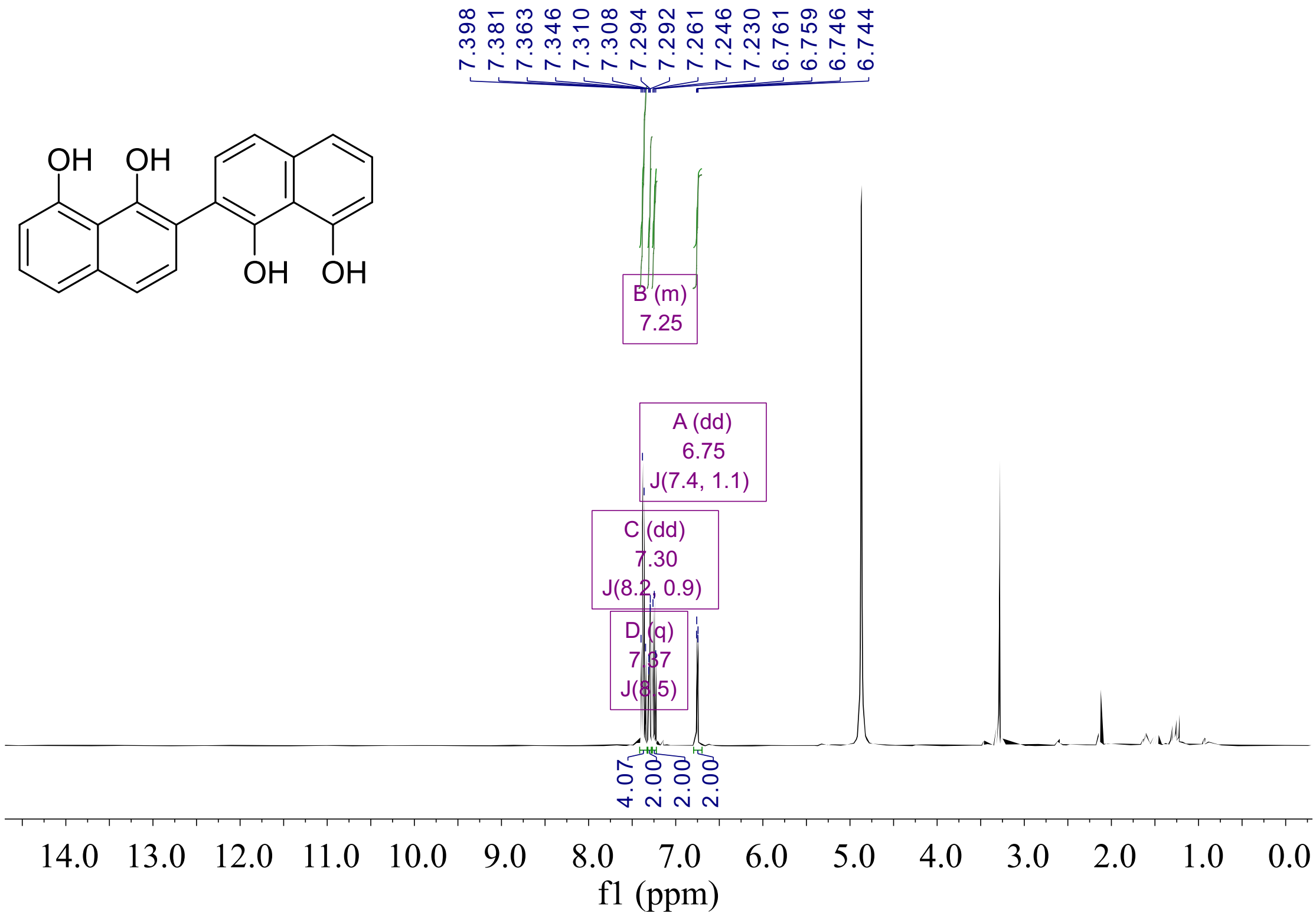

^{1}H NMR spectrum of 2-2'-dihydroxynaphthalene dimer **Figure 6D** in CD_3OD (500 MHz).

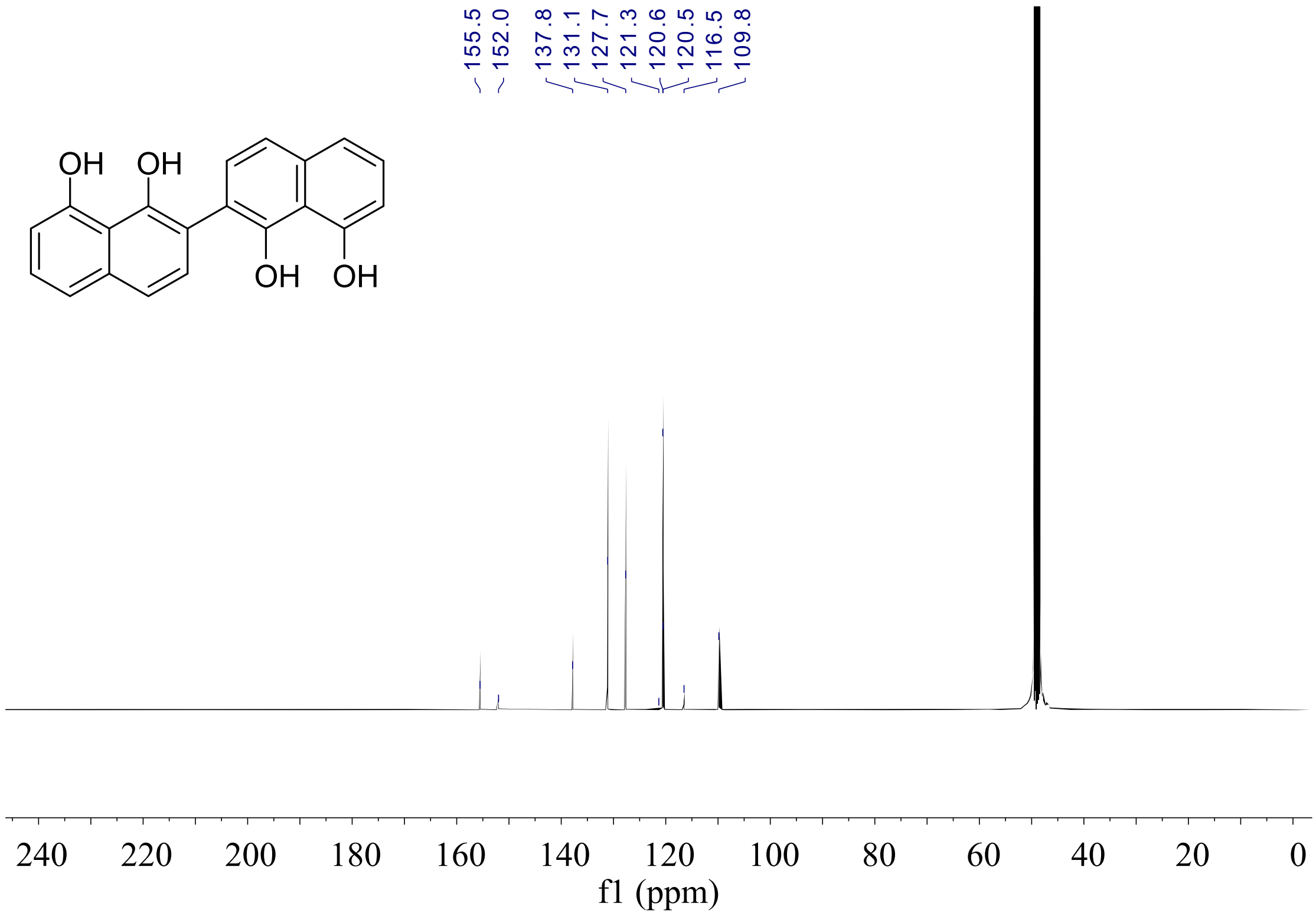

^{13}C NMR spectrum of 2-2'-dihydroxynaphthalene dimer in CD_3OD (125 MHz).
