# Supplementary material for: Intermolecular Carbon–Carbon Naphthalene Coupling Involves a Radical Mechanism Driven by Cytochrome P450 in Alternaria alternata
Source: Angew Chem Int Ed Engl. 2026 Sep 18;65(46):e2505284. doi: 10.1002/anie.2505284 (PMC13749262; doi:10.1002/anie.2505284)
Supplement: Supplementary file 3 [file ANIE-65-e2505284-s003.zip › Table S1 Proof of mutant strains.pdf]

Table S1: Proof of the mutant strains. The mutant strains generated through CRISPR-Cas9 were confirmed by PCR and DNA sequencing using primers located upstream and downstream of the protospacers. The primers used to confirm the mutant strains are listed in **Table S2**. The protospacers are underlined; the start codon (red); the stop codon (blue); 5' un-translated region (UTR) (gray); 3' UTR (gray); M: 1 kb gene ruler; WT: wild type (ATCC 66981 or SMW35).

Mutants	PCR Testing Results	Sequencing Results	Deletion Region
△*veA* (in ATCC 66981)	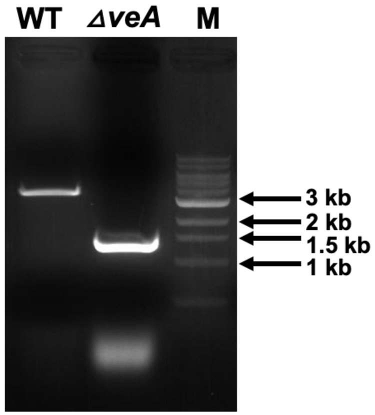	***veA*** GCGATGCGGACGTGGCCCTGCGGCATCCACGAGATGCGCTCAT<br>***△veA*** GCGATGCGGACGTGGCCCTGCGGCATCCACGAGA------------------<br>***veA*** GGCAGTCAGTGTCAGAGACATGT***************************************<br>***△veA*** --<br>***veA*** ***CAACGTCAACGTCATGTACCTGCAGTCGGTCACTGAAGTTGT<br>***△veA*** --GTCGGTCACTGAAGTTGT	2,034-bp deletion: 2,034 bp of the ORF

△*atbA* (in ATCC 66981)	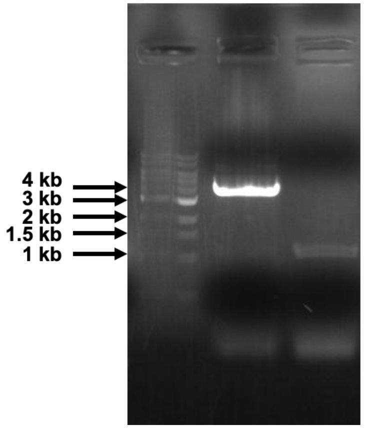	***atbA*** CCAATGAGCAA****ATGCTCGCAATGTGTTGCAAGAGGATAT*****<br>***ΔatbA*** CCAATGAGCAA****ATGCTCGCAATGTGTTGC--------------------------<br><br>***atbA*** ******************AGGTACTTCTTAGATAGATGGAGGTCATGATCAC<br>***ΔatbA*** ---<br><br>***atbA*** *********AGTATTTGTTCTAAAGGTAAACACTGTCTTTATAAGTTCGA<br>***ΔatbA*** ----------AGTATTTGTTCTAAAGGTAAACACTGTCTTTATAAGTTCGA	2,616-bp deletion: 2,455 bp of the ORF and 159 bp of the 3' UTR
△*atbA* (in SMW35)	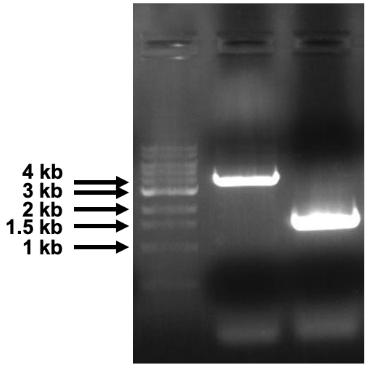	***atbA*** CCAATGAGCAA****ATGCTCGCAATGTGTTGCAAGAGGATAT*****<br>***ΔatbA*** CCAATGAGCAA****ATGCTCGCAATGTGTTGC--------------------------<br><br>***atbA*** ******************AGGTACTTCTTAGATAGATGGAGGTCATGATCAC<br>***ΔatbA*** --TGGAGGTCATGATCAC	2,443-bp deletion: 2,443 bp of the ORF

△atbB (in SMW35)

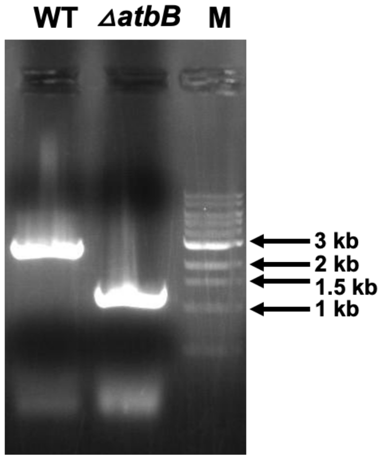

atbB GCGGCTGAGGCTGATGATC***************ATGTCATCTTTTCCC****
⊿atbB GCGGCTGAGGCTGATGATC***************ATGTCATCTTTTCCC****

atbB ***TTATCGCTTGCCGAACACGGTCGAAAGGCGAA******************
⊿atbB ***TTATCGCTTGCCGAACACGG--

atbB ***TTGTAGGGTAGA***CCAGACCTGATATAGAACGCCGGCAC****
⊿atbB --

atbB ***GCGTCAATGCAGACTTCGAGCGATTGGGCGCATGCCGAAGA
⊿atbB ------------CAATGCAGACTTCGAGCGATTGGGCGCATGCCGAAGA

1,327-bp deletion: 789 bp of the ORF and 538 bp of the 3' UTR

△atbC (in SMW35)

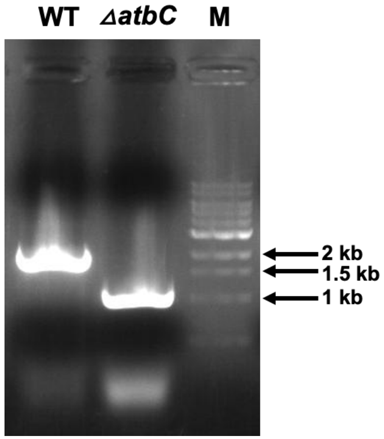

atbC AACGGATCGAGAGGAAACATCTTGGTGGTCTTGGTAT************
⊿atbC AACGGATCGAGAGGAAACATCT--

atbC ***ATGCTCACCTTCCAACACCTTC************************************
⊿atbC ---

atbC ***GCTTAGATGTGTGGGCGAGTATCAGCTGGAGGTGACCAGGC
⊿atbC --TGGAGGTGACCAGGC

815-bp deletion: 222 bp of the 5' UTR, 573 bp of the ORF and 20 bp of the 3' UTR

△*atbD* (in SMW35)

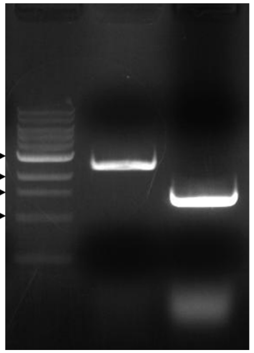

atbD TTGCCATCTCTAGGAGATGGCCCGAAGGACGGCCTG*************
Δ*atbD* TTGCCATCTCTAGGAGATGGCC---

atbD **ATGGCATTCTTGGCATCATTC***
Δ*atbD* --

atbD ***TGATGACTGCATGTGGAAACCCGGAGGGTAATGTTTTCC******
Δ*atbD* --- GGAGGGTAATGTTTTCC******

atbD ****ATTACAACTCTTTCGCTTAATCACTTCTCGCGTTGCATTCTTG
Δ*atbD* ****ATTACAACTCTTTCGCTTAATCACTTCTCGCGTTGCATTCTTG

1,520-bp deletion: 139 bp of the 5' UTR and 1,381 bp of the ORF

△*atbE* (in SMW35)

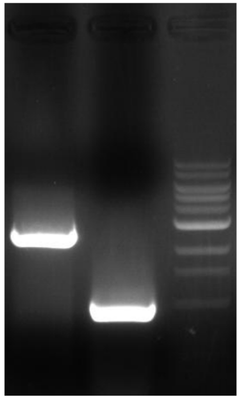

atbE GGCAATCATGATACAAGCAAGTGTTCAATCAGCCTCACAGG****
Δ*atbE* GGCAATCATGATACAAGCAAGTGTTCAATCAGCCTCACAGG****

atbE ***GCATGGGAAGACCCTACCCTGAGTCGTGGTCTGA**************
Δ*atbE* ***GCATGGGAAGACCCTACCCTGAG---------------------------------------

atbE ***CGTGTCGATGTTCGAAGAGGCATTGGCAGCTCATCCTGATCT
Δ*atbE* ---CATTGGCAGCTCATCCTGATCT

atbE TGCTATCTGAATGGGTCTTTGTCTAAAACCCTTGGGTATTGAGA
Δ*atbE* TGCTATCTGAATGGGTCTTTGTCTAAAACCCTTGGGTATTGAGA

1,023-bp deletion: 1,023 bp of the ORF

△*atbF*(△*atbA/atbF* double *mutant* in SMW35)	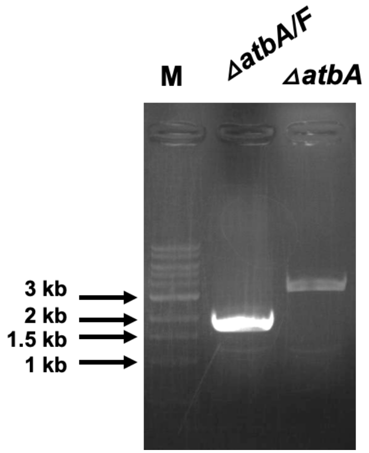	***atbF*** GTTCATGACGCTACGGCAC***************ATGAAGGAAGGTGT****<br>***△atbF*** GTTCATGACGCTACGGCAC***************ATGAAGGAAGGTGT****<br><br>***atbF*** ***ATGAGTTCCACAGTCTTCAAGACATCGGTTGG*******************<br>***△atbF*** ***ATGAGTTCCACAGTCTTCAAG---<br><br>***atbF*** ***GCGTAGAGGTGG***TAGCATATTAGCGAGCGTATAGGGCTCT<br>***△atbF*** ---TATAGGGCTCT	1,603-bp deletion: 1,487 bp of the ORF and 116 bp of the 3’ UTR
△*atbG* (in SMW35)	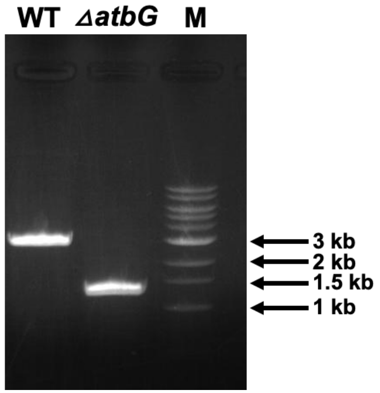	***atbG*** AATCAGGGCGCATGCAAAGGACCGAAGGAGAGAA*****************<br>***△atbG*** AATCAGGGCGCATGCAAAGGAC---<br><br>***atbG*** **ATGCCGTCAGCTACAAGTCC***<br>***△atbG*** --<br><br>***atbG*** ***AGAGTACGGGTCATAGAGTTGACGTATGGCTCGTGTATTAGC<br>***△atbG*** ---GTATGGCTCGTGTATTAGC	1,633-bp deletion: 202 bp of the 5’ UTR, 1,424 bp of the ORF and 7 bp of the 3’ UTR
			1,093-bp deletion:

△*atbH* (in SMW35)	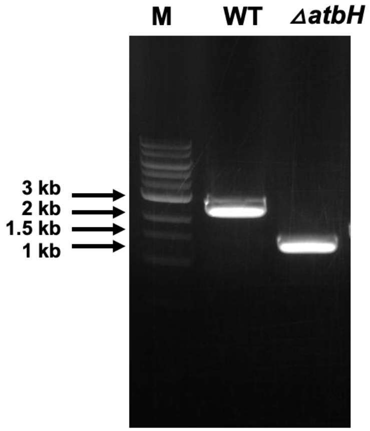	**199 bp of the 5' UTR and 894 bp of the ORF**
	atbH ATCCCCAAAACACGAACGCCATTTCAA********************************<br>***⊿atbH*** ATCCCCAAAACACGAACGCCATTTCAA-----------------------------------<br><br>***atbH*** **ATGGTATTGCTTACCACCCTCAAAAATATCGCCGTGGTTGG****<br>***⊿atbH*** ---<br><br>***atbH*** **GGCCTGAACAATGACGAGCTAGGCCTTCCGAAAGAGGATCTG<br>***⊿atbH*** ---GATCTG<br><br>***atbH*** ***TCACAAGAACATGTACTCGTAGACTGTCTACCTTCTTCGCATG<br>***⊿atbH*** ***TCACAAGAACATGTACTCGTAGACTGTCTACCTTCTTCGCATG	
