# Supplementary material for: Intermolecular Carbon–Carbon Naphthalene Coupling Involves a Radical Mechanism Driven by Cytochrome P450 in Alternaria alternata
Source: Angew Chem Int Ed Engl. 2026 Sep 18;65(46):e2505284. doi: 10.1002/anie.2505284 (PMC13749262; doi:10.1002/anie.2505284)
Supplement: Supplementary file 3 [file ANIE-65-e2505284-s003.zip › Table S2 oligonucleotides used in this study.pdf]

Table S2: Olionucleotides used in this study.

Oligonucleotide	Sequence 5' → 3'
CRISPY_fw_2.0/3.0	GGTCATAGCTGTTTCCGCTGA
CRISPY_re_TtrpCkurz/PtrpC	CTTCAATATCAGTTAACGTCGAGCCAAGAGCGGATTCC
CRISPY_fw_PtrpC	GACGTTAACTGATATTGAAGGAG
CRISPY_re_TtrpClang/Vect	GTCTCGGCTGAGGTCTTAATTCTAGAAAGAAGGATTACCTCTAAAC
Protospacer veA 1 fw	GTCCGTGAGGACGAAACGAGTAAGCTCGTCACCTTCCAACACCTTCAGCTGTTTTAGAGCTAGAAATAGCAAGTTAAA
HH veA 1 rev	GACGAGCTTACTCGTTTCGTCCTCACGGACTCATCAGACCTTCCGGTGATGTCTGCTCAAGCG
Protospacer veA 2 fw	GTCCGTGAGGACGAAACGAGTAAGCTCGTCTGTGGGCGAGTATCAGCTGGGTTTTAGAGCTAGAAATAGCAAGTTAAA
HH veA rev mit PtrpC	GACGAGCTTACTCGTTTCGTCCTCACGGACTCATCAGTGTGGGATCGATGCTTGGGTAGAATAGG
veA-kotest-fw	GGCCTGAGCAAGTATGCATC
veA-kotest-rev	GGTAGGTTGGAGGGGTACT
Protospacer atb 1 fw	GTCCGTGAGGACGAAACGAGTAAGCTCGTCTGCTCGCAATGTGTTGCAAGGTTTTAGAGCTAGAAATAGCAAGTTAAA
HH atb 1 rev	GACGAGCTTACTCGTTTCGTCCTCACGGACTCATCAGTGCTCGCGGTGATGTCTGCTCAAGCG
Protospacer atbA 2 fw	GTCCGTGAGGACGAAACGAGTAAGCTCGTCGGTACTTCTTAGATAGATGGGTTTTAGAGCTAGAAATAGCAAGTTAAA
HH atb atbA rev mit PtrpC	GACGAGCTTACTCGTTTCGTCCTCACGGACTCATCAGGGTACTATCGATGCTTGGGTAGAATAGG
atbA-kotest-fw	CGATTTAAGCAGCTAACAGTCTC
atbA-kotest-rev	AGGTTGGCAGTCGGTTGC
Protospacer atbB 1 fw	GTCCGTGAGGACGAAACGAGTAAGCTCGTCGCTTGCCGAACACGGTCGAAGTTTTAGAGCTAGAAATAGCAAGTTAAA
HH atbB 1 rev	GACGAGCTTACTCGTTTCGTCCTCACGGACTCATCA

	GGCTTGCCGGTGATGTCTGCTCAAGCG
Protospacer atbB 2 fw	GTCCGTGAGGACGAAACGAGTAAGCTCGTCCCAGACCTGATATAGAACGCGTTTTAGAGCTAGAAATAGCAAGTTAAA
HH atbB rev mit PtrpC	GACGAGCTTACTCGTTTCGTCCTCACGGACTCATCAGCCAGACATCGATGCTTGGGTAGAATAGG
atbB-kotest-fw	GCGGCTGAGGCTGATGATC
atbB-kotest-rev	CGATATCGCGTCCTTTCCAAG
Protospacer atbC 1 fw	GTCCGTGAGGACGAAACGAGTAAGCTCGTCACCTTCCAACACCTTCAGCTGTTTTAGAGCTAGAAATAGCAAGTTAAA
HH atbC 1 rev	GACGAGCTTACTCGTTTCGTCCTCACGGACTCATCAGACCTTCCGGTGATGTCTGCTCAAGCG
Protospacer atbC 2 fw	GTCCGTGAGGACGAAACGAGTAAGCTCGTCTGTGGGCGAGTATCAGCTGGGTTTTAGAGCTAGAAATAGCAAGTTAAA
HH atbC rev mit PtrpC	GACGAGCTTACTCGTTTCGTCCTCACGGACTCATCAGTGTGGGATCGATGCTTGGGTAGAATAGG
atbC-kotest-fw	GACAGCGCGCAGTTCCTATT
atbC-kotest-rev	TGAAGACTGCAGTCGAATGACC
Protospacer atbD 1 fw	GTCCGTGAGGACGAAACGAGTAAGCTCGTCATCTCTAGGAGATGGCCCGAGTTTTAGAGCTAGAAATAGCAAGTTAAA
HH atbD 1 rev	GACGAGCTTACTCGTTTCGTCCTCACGGACTCATCAGATCTCTCGGTGATGTCTGCTCAAGCG
Protospacer atbD 2 fw	GTCCGTGAGGACGAAACGAGTAAGCTCGTCGACTGCATGTGGAAACCCGGGTTTTAGAGCTAGAAATAGCAAGTTAAA
HH atbD rev mit PtrpC	GACGAGCTTACTCGTTTCGTCCTCACGGACTCATCAGGACTGCATCGATGCTTGGGTAGAATAGG
atbD-kotest-fw	CCATCGACCTGCAGCACG
atbD-kotest-rev	CTGCTTCTGCTTCGTTTGACC
Protospacer atbE 1 fw	GTCCGTGAGGACGAAACGAGTAAGCTCGTCGAAGACCCTACCCTGAGTCGGTTTTAGAGCTAGAAATAGCAAGTTAAA
HH atbE 1 rev	GACGAGCTTACTCGTTTCGTCCTCACGGACTCATCAGGAAGACCGGTGATGTCTGCTCAAGCG
Protospacer atbE	GTCCGTGAGGACGAAACGAGTAAGCTCGTCGTCGA

2 fw	TGTTCGAAGAGGCATGTTTTAGAGCTAGAAATAGCAAGTTAAA
HH atbE rev mit PtrpC	GACGAGCTTACTCGTTTCGTCCTCACGGACTCATCAGGTCGATATCGATGCTTGGGTAGAATAGG
atbE-kotest-fw	GCAAGTGTTCAATCAGCCTCAC
atbE-kotest-rev	CATGACAGATGCATTGCTACGAC
Protospacer atbF 1 fw	GTCCGTGAGGACGAAACGAGTAAGCTCGTCTTCCACAGTCTTCAAGACATGTTTTAGAGCTAGAAATAGCAAGTTAAA
HH atbF 1 rev	GACGAGCTTACTCGTTTCGTCCTCACGGACTCATCAGTTCCACCGGTGATGTCTGCTCAAGCG
Protospacer atbF 2 fw	GTCCGTGAGGACGAAACGAGTAAGCTCGTCTAGCATATTAGCGAGCGTATGTTTTAGAGCTAGAAATAGCAAGTTAAA
HH atbF rev mit PtrpC	GACGAGCTTACTCGTTTCGTCCTCACGGACTCATCAGTAGCATATCGATGCTTGGGTAGAATAGG
atbF-kotest-fw	CTGCCTCAAGTCTCTGAACTATC
atbF-kotest-rev2	CTAATGTTGTGCATTGTTCACCTG
Protospacer atbG 1 fw	GTCCGTGAGGACGAAACGAGTAAGCTCGTCGGGCGCATGCAAAGGACCGAGTTTTAGAGCTAGAAATAGCAAGTTAAA
HH atbG 1 rev	GACGAGCTTACTCGTTTCGTCCTCACGGACTCATCAGGGGCGCCGGTGATGTCTGCTCAAGCG
Protospacer atbG 2 fw	GTCCGTGAGGACGAAACGAGTAAGCTCGTCCGGGTCATAGAGTTGACGTAGTTTTAGAGCTAGAAATAGCAAGTTAAA
HH atbG rev mit PtrpC	GACGAGCTTACTCGTTTCGTCCTCACGGACTCATCAGCGGGTCATCGATGCTTGGGTAGAATAGG
atbG-kotest-fw	GATCGCTCGGAACAGTCGA
atbG-kotest-rev	TTGGCCCTATCGAGGAAGG
Protospacer atbH 1 fw	GTCCGTGAGGACGAAACGAGTAAGCTCGTCCACCCTCAAAAATATCGCCGGTTTTAGAGCTAGAAATAGCAAGTTAAA
HH atbH 1 rev	GACGAGCTTACTCGTTTCGTCCTCACGGACTCATCAGCACCCTCGGTGATGTCTGCTCAAGCG
Protospacer atbH 2 fw	GTCCGTGAGGACGAAACGAGTAAGCTCGTCGGCCTGAACAATGACGAGCTGTTTTAGAGCTAGAAATAGCAAGTTAAA

HH atbH rev mit PtrpC	GACGAGCTTACTCGTTTCGTCCTCACGGACTCATCAGGGCCTGATCGATGCTTGGGTAGAATAGG
atbH-kotest-fw	CCCAAGTAGATGACGCAGAC
atbH-kotest-rev	CCTCCAAACCAACAATCCTTATG
PatbA-pJet-fw	GTTTTTCAGCAAGATTTAATGTCATATCCCACCTATCGATAC
TatbA-pJet-rev	CCACTAGCATTACACTTTTAGCGAGAGTCTGCCTTGTATC
PatbB-pJet-fw	GTTTTTCAGCAAGATTTAATGAGATGGTGGAGAGAGCC
TatbB-pJet-rev	CCACTAGCATTACACTTTTACGATATCGCGTCCTTTCCAAG
PatbC-pJet-fw	GTTTTTCAGCAAGATTTAATGCCGGTGAAGGTTGTGGG
TatbC-pJet-rev	CCACTAGCATTACACTTTTAGGCTTCTTTGAATTCAACCTGCC
PatbD-pJet-fw	GTTTTTCAGCAAGATTTAATTCGTCCTAACGGTGCTGCAA
TatbD-pJet-rev	CCACTAGCATTACACTTTTAATTCACGCTGCACTACATGAAC
PatbE-pJet-fw	GTTTTTCAGCAAGATTTAATGCAAAGAAGGGCCGTAGTGA
TatbE-pJet-rev	CCACTAGCATTACACTTTTAAGCGCATCGCAGACAACATG
PatbG-pJet-fw	GTTTTTCAGCAAGATTTAATGATCGCTCGGAACAGTCGA
TatbG-pJet-rev	CCACTAGCATTACACTTTTAGGAAGCTATGGAACGTTCGG
PatbH-pJet-fw	GTTTTTCAGCAAGATTTAATCCCAAGTAGATGACGCAGAC
TatbH-pJet-rev	CCACTAGCATTACACTTTTACACTATTACAGGCCATGAGGC
PBpatbA-pJet-fw	GTTTTTCAGCAAGATTTAATGAGCTAGTGTCGCGATATGTAA
TBpatbA-pJet-rev	CCACTAGCATTACACTTTTATCTCGACGTCTGCGTCGA
atbC-re-fw	TCATTCGACTGCAGTCTTCATCACGCAGCAAATCAAGACGC

atbC-re-rev	CGTCTTGATTTGCTGCGTGATGAAGACTGCAGTCGAATGACC
TatbA-pJet-pacI-rev	CCACTAGCATTACACTTTTATTAATTAAGCGAGAGTCTGCCTTGTATC
PatbC-TatbA-fw	GATACAAGGCAGACTCTCGCTTAATGCCGGTGAAGGTTGTGGG
PatbC-pacI-rev	CCACTAGCATTACACTTTTATTAATTAAATTGGCGATTTCGAAAGTTTATCGG
P. osolnii tefA-qPCR-fw	GTATTGACCAGCGTACCATCG
P. osolnii tefA-qPCR-rev	GTCAAGAACCCAAGCGTACTTG
P. crustosum tefA-qPCR-fw	GTATTGACCAGCGTACCATCG
P. crustosum tefA-qPCR-rev	TCAAGAACCCAGGCGTACTTG
A. alternata tefA-qPCR-fw	TGCTCGCAAGATCATGAAGGC
A. alternata tefA-qPCR-rev	GATTGAAGGTCGTCATGATCAGT
PatbC-pacI-GA5-mCherry-fw	AAACTTTCGAAATCGCCAATTTAATTAAGGTGCGGGTGCGGGTGCGGGTGCGGGTGCGAGCAAGGGCGAGGAGGATAA
TtrpC-pJet-rev	CCACTAGCATTACACTTTTATGGGGGGAGTTTAGGGAAAGA
PatbC-ATG-mCherry-fw	AAACTTTCGAAATCGCCAATATGAGCAAGGGCGAGGAGGATAA
mCherry-pacI-pJet-rev	CCACTAGCATTACACTTTTATTAATTAACGCACCCGCACCCGCACCCGCACCCGCACCAGCGTAATCTGGAACATCGTATG
PatbC-atbB-fw	AAACTTTCGAAATCGCCAATATGTCATCTTTTCCCGCATTCC
mCherry-atbB-rev	CCCGCACCCGCACCCGCACCCAAGTTCTTGGTAATACCAGGACT
GA5-atbC-fw	CGGGTGCGGGTGCGGGTGCGATGCTCACCTTCCAACACCTTC
PatbC-atbD-fw	AAACTTTCGAAATCGCCAATATGGCATTCTTGGCATCATTCGT

mCherry-atbD-rev	CCCGCACCCGCACCCGCACCAGCGAAAGAGTTGTAATGGTTGAAC
PatbC-atbE-fw	AAACTTTCGAAATCGCCAATATGATACAAGCAAGTGTTCAATCAG
mCherry-atbE-rev	CCCGCACCCGCACCCGCACCGATAGCAAGATCAGGATGAGCT
GA5-atbF-fw	CGGGTGCGGGTGCGGGTGCGATGAAGGAAGGTGTCGTTACGCCAC
atbF-pJet-rev	CCACTAGCATTACACTTTTACTAATGTTGTGCATTGTTCACCTG
PatbC-atbG-fw	AAACTTTCGAAATCGCCAATATGCCGTCAGCTACAAGTCC
atbG-GA5-rev	CCCGCACCCGCACCCGCACCTGACCCGTACTCTGGCGG
PatbC-atbH-fw	AAACTTTCGAAATCGCCAATATGGTATTGCTTACCACCCTCA
atbH-GA5-rev	CCCGCACCCGCACCCGCACCCGAGTACATGTTCTTGTGATAATCC
ITS1	TCCGTAGGTGAACCTGCGG
ITS4	TCCTCCG CTTATTGATATGC
atbE-R116A-fw	CTCGCAAAGCGTGGAGCACACTACTCAAACGCAGCGCCTA
atbE-R116A-rev	GGCGCCTGGGATATTGGGTGTAGGCGCTGCGTTTGAGTAG
atbE-L132A-fw	CAGGCGCCAAAGTCGATGCGGAATATGCACCTCCTCTGGACAA
atbE-L132A-rev	GTCTCGTAAGTGCTTCATTGTTGTCCAGAAGAGGTGCATATTC
atbE-P133A-fw	CAGGCGCCAAAGTCGATGCGGAATATCTCGCACTTCTGGACAA
atbE-P133A-rev	GTCTCGTAAGTGCTTCATTGTTGTCCAGAAGTGCGAGATATTC
atbE-L215A-fw	AGGAAGGTGCATTCGCAGTTGGTGCACTCTCGGCCATCT
atbE-L215A-rev	CGGCAATTGACCTGCTGGAGAGATGGCCGAGAGTGCACC
atbE-L216A-fw	AGGAAGGTGCATTCGCAGTTGGTCTAGCATCGGCCATCT

atbE-L216A-rev	CGGCAATTGACCTGCTGGAGAGATGGCCGATGCTAGACC
atbE-I219A-fw	AGGAAGGTGCATTCGCAGTTGGTCTACTCTCGGCCGCAT
atbE-I219A-rev	CGGCAATTGACCTGCTGGAGATGCGGCCGAGAGTAGACC
atbE-I309A-fw	TTCGCAGTTGGTATGCTTGCGGCAACAGCATCGCTGCCT
atbE-I309A-rev	GTCTGGATGGGCGAGGACATAGGCAGCGATGCTGTTGCC
atbE-T310A-fw	TTCGCAGTTGGTATGCTTGCGATCGCAGCATCGCTGCCT
atbE-T310A-rev	GTCTGGATGGGCGAGGACATAGGCAGCGATGCTGCGATC
atbE-L313A-fw	TTCGCAGTTGGTATGCTTGCGATCACAGCATCGGCACCT
atbE-L313A-rev	GTCTGGATGGGCGAGGACATAGGTGCCGATGCTGTGATC
atbE-P314A-fw	TTCGCAGTTGGTATGCTTGCGATCACAGCATCGCTGGCA
atbE-P314A-rev	GTCTGGATGGGCGAGGACATTGCCAGCGATGCTGTGATC
atbE-V379A-fw	GCTAATCCGCTGGAGGCCCCCAGTCCCCACTGGTGCACC
atbE-V379A-rev	CGTCCCTCTCGGCCTCGTGGGGTGCACCAGTGGGGACTG
atbE-I502A-fw	TGTACACACATTCGAGTGGACAGACTTGCTCGCAACTCGAC
atbE-I502A-rev	GTCGAACACAAAAACCTCCGGTCGAGTTGCGAGCAAGTCTG
PgpdA (Aa) -pJet-fw	GTTTTTCAGCAAGATTTAATGCTTGAGCTCAAGCTGTAGC
mcpA-PgpdA (Aa)-fw	AGCACTACACTCAGTTCATCATGGGTCTTCCTTCAATATTATTAGC
PgpdA (ATG)-mcpA-rev	AATAATATTGAAGGAAGACCCATGATGAACTGAGTGTAGTGCTGC
T109432-pJet-rev	CCACTAGCATTACACTTTTACAACCCTGTCGCTCAGTAAAC
PgpdA-atbC-fw	AGCACTACACTCAGTTCATCATGCTCACCTTCCAAC

	ACCTTC
PgpdA-atbC-rev	AGGTGTTGGAAGGTGAGCATGATGAACTGAGTGTA GTGCTGC
PgpdA-atbE-fw	AGCACTACACTCAGTTCATCATGATACAAGCAAGTG TTCAATCAG
PgpdA -atbE-rev	TGAACACTTGCTTGTATCATGATGAACTGAGTGTAG TGCTGC
