# Supplementary material for: Intermolecular Carbon–Carbon Naphthalene Coupling Involves a Radical Mechanism Driven by Cytochrome P450 in Alternaria alternata
Source: Angew Chem Int Ed Engl. 2026 Sep 18;65(46):e2505284. doi: 10.1002/anie.2505284 (PMC13749262; doi:10.1002/anie.2505284)
Supplement: Supplementary file 3 [file ANIE-65-e2505284-s003.zip › Table S3 Strains used in this study.pdf]

Table S3: Strains used in this study.

Strain	Genotype or description	Source
Alternaria alternata		
ATCC 66981	Parental strain	Christopher Lawrence, Virginia Tech, Blacksburg, VA, USA
SMW35	*⊿pksI*	[1]
SJG64	*⊿veA* in ATCC 66981	This study
SJG29	*⊿pksI/cmrA*	[2]
SOI4	*⊿hogA*	[3]
SJG65	*⊿atbA* in SMW35	This study
SJG66	*⊿atbB* in SMW35	This study
SJG67	*⊿atbC* in SMW35	This study
SJG68	*⊿atbD* in SMW35	This study
SJG69	*⊿atbE* in SMW35	This study
SJG76	*⊿atbA* in ATCC 66981	This study
SJG77	*⊿atbA* in ATCC 66981 complemented with *Alternaria alternata atbA*	This study
SJG101	*⊿atbF* in SJG65	This study
SJG102	*⊿atbB* in SMW35 complemented with *Alternaria alternata atbB*	This study
SJG103	*PatbA::atbA::TatbA* in SJG67, overexpressing *atbA*	This study
SJG104	*PatbA::atbA::TatbA* in SJG68, overexpressing *atbA*	This study
SJG105	*PatbA::atbA::TatbA* in SJG69, overexpressing *atbA*	This study
SJG106	*⊿atbG* in SMW35	This study
SJG107	*⊿atbH* in SMW35	This study
SJG108	*PatbA::atbA::TatbA PatbB::atbB::GA5::mCherry::TtrpC* in SJG66	This study
SJG109	*PatbA::atbA::TatbA*	This study

	PatbC::mCherry::GA5::atbC::TatbC in SJG67	
SJG110	*PatbA::atbA::TatbA* *PatbC::atbD::GA5::mCherry::TtrpC* in SJG68	This study
SJG111	*PatbA::atbA::TatbA* *PatbC::atbE::GA5::mCherry::TtrpC* in SJG69	This study
SJG112	*PatbA::atbA::TatbA* *PatbC::mCherry::GA5::atbF::TatbF* in SJG101	This study
SJG113	*PatbA::atbA::TatbA* *PatbC::atbG::GA5::mCherry::TtrpC* in SJG106	This study
SJG114	*PatbA::atbA::TatbA* *PatbC::atbH::GA5::mCherry::TtrpC* in SJG107	This study
SJG115	*⊿atbA* in ATCC 66981 complemented with *Bipolaris maydis atbA*	This study
SJG121	*atbE*^{R116A} complemented in SJG69	This study
SJG122	*atbE*^{L132A} complemented in SJG69	This study
SJG123	*atbE*^{P133A} complemented in SJG69	This study
SJG124	*atbE*^{L125A} complemented in SJG69	This study
SJG125	*atbE*^{L126A} complemented in SJG69	This study
SJG126	*atbE*^{I219A} complemented in SJG69	This study
SJG127	*atbE*^{I309A} complemented in SJG69	This study
SJG128	*atbE*^{T310A} complemented in SJG69	This study
SJG129	*atbE*^{I313A} complemented in SJG69	This study
SJG130	*atbE*^{P314A} complemented in SJG69	This study
SJG131	*atbE*^{V379A} complemented in SJG69	This study
SJG132	*atbE*^{I502A} complemented in SJG69	This study
SJG28	*⊿pksA* in SMW35	[2]
SJG133	*PgpdA::mcpA::TmcpA* in SJG28	This study
SJG134	*PgpdA::mcpA::TmcpA* in SJG69	This study
SJG135	*PgpdA::atbC::TatbC* and *PgpdA::atbE::TatbE* in SJG28	This study

Bipolaris maydis		
SJG116	*PBmatbA::BmatbA::TBmatbA* in *Bipolaris maydis,* overexpressing *BmatbA*	This study
Alternaria brassicicola		
SJG117	*atb gene cluster* in *A. brassicicola*	This study
SJG118	*atb gene cluster PatbG::atbG::TatbG* in *A. brassicicola*	This study
SJG119	*atb gene cluster PatbH::atbH::TatbH* in *A. brassicicola*	This study
SJG120	*atb gene cluster PatbG::atbG::TatbG PatbH::atbH::TatbH* in *A. brassicicola*	This study
Penicillum spp.		
Penicillum crustosum		This study
Penicillum olsonii		This study
Aspergillus nidulans		
Aspergillus nidulans FGSC A4		Fungal Genetics Stock Centre

[1] M. Wenderoth, F. Garganese, M. Schmidt-Heydt, S. T. Soukup, A. Ippolito, S. M. Sanzani, R. Fischer, *Mol Microbiol* **2019**, *112*, 131-146.

[2] J. Gao, M. Wenderoth, M. Doppler, R. Schuhmacher, D. Marko, R. Fischer, *mBio* **2022**, *13*, e0021922.

[3] O. Igbalajobi, Z. Yu, R. Fischer, *mBio* **2019**, *10*, e00371-00319.
