# Supplementary material for: Intermolecular Carbon–Carbon Naphthalene Coupling Involves a Radical Mechanism Driven by Cytochrome P450 in Alternaria alternata
Source: Angew Chem Int Ed Engl. 2026 Sep 18;65(46):e2505284. doi: 10.1002/anie.2505284 (PMC13749262; doi:10.1002/anie.2505284)
Supplement: Supplementary file 3 [file ANIE-65-e2505284-s003.zip › Table S4 plasmids used in this study.pdf]

Table S4: Plasmids used in this study.

Name	Description	Reference
pFC332	*tef1*(p)*::cas9::tef1*(t); *gpdA*(p)*::gpdA*(t); *hph; ampR*; AMA1	Uffe H. Mortensen, Technical University of Denmark
pFC334	*tef1*(p)*::cas9::tef1*(t); *gpdA*(p)*::sgRNA-AnyA::gpdA*(t); *Afpyr4; ampR*; AMA1	Uffe H. Mortensen, Technical University of Denmark
pAK4	pJET1.2 blunt + *trpC*(p)*::sgRNA-AnyA::trpC*(t)	This work
pJG33	pJET1.2 blunt + *hph*; *ampR*	[1]
pEH4	*tef1*(p)*::cas9::tef1*(t); *gpdA*(p)*::veA-sgRNAcassette1::gpdA*(t)*::trpC*(p)*::veA-sgRNAcassette2::trpC*(t); *hph*; *ampR*	This work
pJG74	*tef1*(p)*::cas9::tef1*(t); *gpdA*(p)*::atbA-sgRNAcassette1::gpdA*(t)*::trpC*(p)*::atbA-sgRNAcassette2::trpC*(t); *hph*; *ampR*	This work
pJG75	*tef1*(p)*::cas9::tef1*(t); *gpdA*(p)*::atbB-sgRNAcassette1::gpdA*(t)*::trpC*(p)*::atbB-sgRNAcassette2::trpC*(t); *hph*; *ampR*	This work
pJG76	*tef1*(p)*::cas9::tef1*(t); *gpdA*(p)*::atbC-sgRNAcassette1::gpdA*(t)*::trpC*(p)*::atbC-sgRNAcassette2::trpC*(t); *hph*; *ampR*	This work
pJG77	*tef1*(p)*::cas9::tef1*(t); *gpdA*(p)*::atbD-sgRNAcassette1::gpdA*(t)*::trpC*(p)*::atbD-sgRNAcassette2::trpC*(t); *hph*; *ampR*	This work
pJG78	*tef1*(p)*::cas9::tef1*(t); *gpdA*(p)*::atbE-sgRNAcassette1::gpdA*(t)*::trpC*(p)*::atbE-sgRNAcassette2::trpC*(t); *hph*; *ampR*	This work
pJG79	*tef1*(p)*::cas9::tef1*(t); *gpdA*(p)*::atbF-sgRNAcassette1::gpdA*(t)*::trpC*(p)*::atbF-sgRNAcassette2::trpC*(t); *hph*; *ampR*	This work
pJG80	*pJET1.2 blunt/atbA(p)::atbA::atbA(t),hph; ampR*	This work

pJG81	*pJET1.2 blunt/atbA-F, hph; ampR*	This work
pMP172	*pJET1.2 blunt/atbA(p)::atbA::atbA(t)::atbC(p)::pacI, hph; ampR*	This work
pJG116	*pJET1.2 blunt/atbB(p)::atbB::atbB(t),hph; ampR*	This work
pJG124	*pJET1.2 blunt/atbA(p)::atbA::atbA(t)::pacI::GA5::mCherry::trpC(t), hph; ampR*	This work
pJG125	*pJET1.2 blunt/atbA(p)::atbA::atbA(t)::atbC(p)::mCherry::GA5::pacI, hph; ampR*	This work
pJG126	*pJET1.2 blunt/atbA(p)::atbA::atbA(t)::atbB(P)::atbB::GA5::mCherry::trpC(t), hph; ampR*	This work
pJG127	*pJET1.2 blunt/atbA(p)::atbA::atbA(t)::atbC(p)::mCherry::GA5::atbC::atbC(t), hph; ampR*	This work
pJG128	*pJET1.2 blunt/atbA(p)::atbA::atbA(t)::atbD(P)::atbD::GA5::mCherry::trpC(t), hph; ampR*	This work
pJG129	*pJET1.2 blunt/atbA(p)::atbA::atbA(t)::atbE(P)::atbE::GA5::mCherry::trpC(t), hph; ampR*	This work
pJG130	*pJET1.2 blunt/atbA(p)::atbA::atbA(t)::atbC(p)::mCherry::GA5::atbF::atbF(t), hph; ampR*	This work
pJG131	*tef1*(p)*::cas9::tef1*(t); *gpdA*(p)*::atbG-sgRNAcassette1::gpdA*(t)*::trpC*(p)*::atbG-sgRNAcassette2::trpC*(t); *hph*; *ampR*	This work
pJG132	*tef1*(p)*::cas9::tef1*(t); *gpdA*(p)*::atbH-sgRNAcassette1::gpdA*(t)*::trpC*(p)*::atbH-sgRNAcassette2::trpC*(t); *hph*; *ampR*	This work
pJG133	*pJET1.2 blunt/atbA(p)::atbA::atbA(t)::atbG(P)::atbG::GA5::mCherry::trpC(t), hph; ampR*	This work
pJG134	*pJET1.2 blunt/atbA(p)::atbA::atbA(t)::atbH(P)::atbH::GA5::mCherry::trpC(t), hph; ampR*	This work
pJG135	*pJET1.2 blunt/atbG(p)::atbG::atbG(t),hph; ampR*	This work
pJG136	*pJET1.2 blunt/atbH(p)::atbH::atbH(t),hph; ampR*	This work
pJG137	*pJET1.2 blunt/BmatbA(p)::BmatbA::BmatbA(t),hph; ampR*	This work

pJG138	*pJET1.2 blunt/atbE(p)::atbE*^{R116A}*::atbE(t),hph; ampR*	This work
pJG139	*pJET1.2 blunt/atbE(p)::atbE*^{L132A}*::atbE(t),hph; ampR*	This work
pJG140	*pJET1.2 blunt/atbE(p)::atbE*^{P133A}*::atbE(t),hph; ampR*	This work
pJG141	*pJET1.2 blunt/atbE(p)::atbE*^{L215A}*::atbE(t),hph; ampR*	This work
pJG142	*pJET1.2 blunt/atbE(p)::atbE*^{L216A}*::atbE(t),hph; ampR*	This work
pJG143	*pJET1.2 blunt/atbE(p)::atbE*^{I219A}*::atbE(t),hph; ampR*	This work
pJG144	*pJET1.2 blunt/atbE(p)::atbE*^{I309A}*::atbE(t),hph; ampR*	This work
pJG145	*pJET1.2 blunt/atbE(p)::atbE*^{T310A}*::atbE(t),hph; ampR*	This work
pJG146	*pJET1.2 blunt/atbE(p)::atbE*^{I313A}*::atbE(t),hph; ampR*	This work
pJG147	*pJET1.2 blunt/atbE(p)::atbE*^{P314A}*::atbE(t),hph; ampR*	This work
pJG148	*pJET1.2 blunt/atbE(p)::atbE*^{V379A}*::atbE(t),hph; ampR*	This work
pJG149	*pJET1.2 blunt/atbE(p)::atbE*^{I502A}*::atbE(t),hph; ampR*	This work
pJG150	*pJET1.2 blunt/gpdA(p)::mcpA::mcpA(t),hph; ampR*	This work
pJG151	*pJET1.2 blunt/gpdA(p)::atbC::atbC(t),hph; ampR*	This work
pJG152	*pJET1.2 blunt/gpdA(p)::atbE::atbE(t),hph; ampR*	This work

[1] J. Gao, M. Wenderoth, M. Doppler, R. Schuhmacher, D. Marko, R. Fischer, *mBio* **2022**, *13*, e0021922.
